# Stereoselective Installation of Five Contiguous Stereogenic Centers in a Double Aldol–Tishchenko Cascade and Evaluation of the Key Transition State through DFT Calculation

**DOI:** 10.1021/acs.orglett.1c02179

**Published:** 2021-08-10

**Authors:** Pamela Mackey, Aneta Turlik, Kaori Ando, Mark E. Light, K. N. Houk, Gerard P. McGlacken

**Affiliations:** † School of Chemistry and Analytical and Biological Chemistry Research Facility, University College Cork, Cork, Ireland; ‡ Department of Chemistry and Biochemistry, University of California, Los Angeles, California 90095-1569, United States; § Department of Chemistry and Biomolecular Science, Faculty of Engineering, Gifu University, Yanagido 1-1, Gifu 501-1193, Japan; ∥ University of Southampton, Chemistry Department, University Road, Southampton SO17 1BJ, United Kingdom

## Abstract

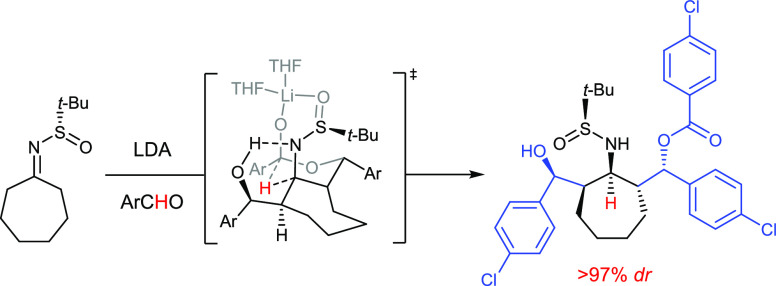

The stereoselective formation of 5 contiguous chiral centers in a single pot reaction is demonstrated using an aldol, aldol–Tishchenko reaction of *N*-*tert*-butyl sulfinimines. One diastereoisomer (from 32 possibilities) predominates, and a series of cyclic and acyclic 3-amino-1,5-diol derivatives are synthesized in good yields (up to 80%) and excellent diastereoselectivities (up to >98:2 dr). Investigations support two reversible aldol steps, and multiple intermediates which are funnelled through a remarkably selective, irreversible, Tishchenko reduction, in a Curtin–Hammett phenomenon. DFT calculations using a disolvated (THF) model reveal the factors controlling stereoselectivity in the final irreversible Tishchenko step.

Tandem asymmetric reactions are a powerful approach to furnish multiple new bonds, and assemble chiral centers in a one-pot process, and can provide a convenient route to build up stereogenic complexity.^1−3^ However, to date, a limited number of one-pot reactions for the construction of multiple contiguous chiral centers have been reported,^1a,4^ and the asymmetric syntheses of five or more stereocenters in one pot via the intermolecular coupling of simple and inexpensive reactants have not been widely achieved.^4^ Additionally, there are a limited number of examples in the literature of the preparation of 5 contiguous stereogenic centers using an enantioselective double aldol–Tishchenko reaction.^5^

Through the use of chiral sulfinylimines, the aldol–Tishchenko reaction has the potential to exploit very simple and cheap starting materials to form 1,3-aminoalcohols (Scheme 1).^6,7^ However, the potential of this and similar processes to build stereopentads asymmetrically is untapped. We now describe a *double* aldol–Tishchenko process that provides a single isomer (from 32 possibilities). Ultimately, enantio- and diastereomerically enhanced 3-amino-1,5-diol derivatives (up to 98% purity) with four and five new chiral centers were accessible. The origins of a single stereodetermining step which installs all 5 stereogenic centers are revealed through mechanistic and detailed DFT studies, and illustrate a remarkable application of the Curtin–Hammett principle. Irrespective of the intriguing methodology and associated selectivity, these highly functionalized entities can provide the basis for a variety of potentially important medicinal scaffolds, and 3-anchor-point ligands, expanding on the well-utilized 1,3 amimoalcohol framework^8^ (Scheme 1).

**Scheme 1:**
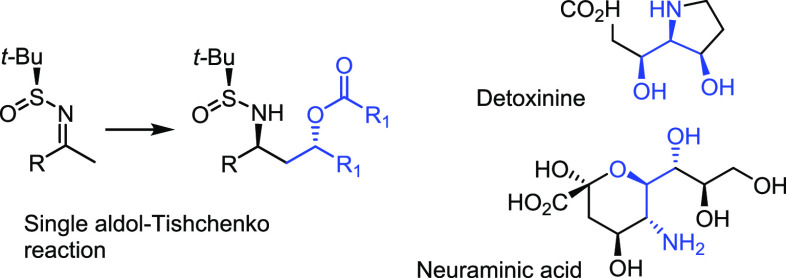


Our initial objective was to utilize symmetrical cyclic sulfinimines in a single-aldol–Tishchenko reaction, which has not yet been reported. However, using the cycloheptanone derived starting material (**1a**), we discovered that propagated double aldol–Tishchenko product **2a** was formed. Indeed, we noticed that a single diastereomer predominated (out of a possible 32), with a *dr* of >90:others.

This result inspired us to further investigate the scope of this methodology (Scheme 2). Aldol acceptors with both electron-donating (**2b**–**f**) and electron-withdrawing substituents (**2g**–**l**) proved successful. For example, the *m*-methoxy **2g**, *p*-chloro **2h**, and *p*-fluoro **2k** analogues were formed with excellent diastereoselectivity (98:2 *dr*). The *p*-*tert*-butyl and *p*-trifluoromethoxy substituted benzaldehydes also gave the intended products with very high levels of diastereoselectivity (**2e** and **2j**). A good yield (62%) and very good diastereoselectivity (95:3:2 *dr*) were also obtained in the case of **2b**. A number of very challenging aldol acceptors were also examined. Notably, the *in situ* intramolecular Tishchenko hydride transfer enables the formation of 3-amino-1,5-diol derivatives containing both an ester and nitrile substituent (**2m**–**o**). These functionalities would otherwise be susceptible to reduction if an external reductant were used (such as in an Ellman-type protocol).^9^ Perhaps surprisingly, product **2p**, which contains the appealing pyridine subunit,^10^ could also be formed in good yield and selectivity. The absolute configuration of the major diastereomer was determined to be (*S*,*R*,*R*,*R*,*S*,*S*) by X-ray crystallographic analysis of products **2j** and **2k**. We were pleased to find that the reaction of cyclopentanone sulfinimine with benzaldehydes generated the 3-amino-1,5-diol derivatives (**3a**–**c**) with very good diastereoselectivity. Use of the cyclohexanone derived substrate proves to be less successful.^11^

**Scheme 2:**
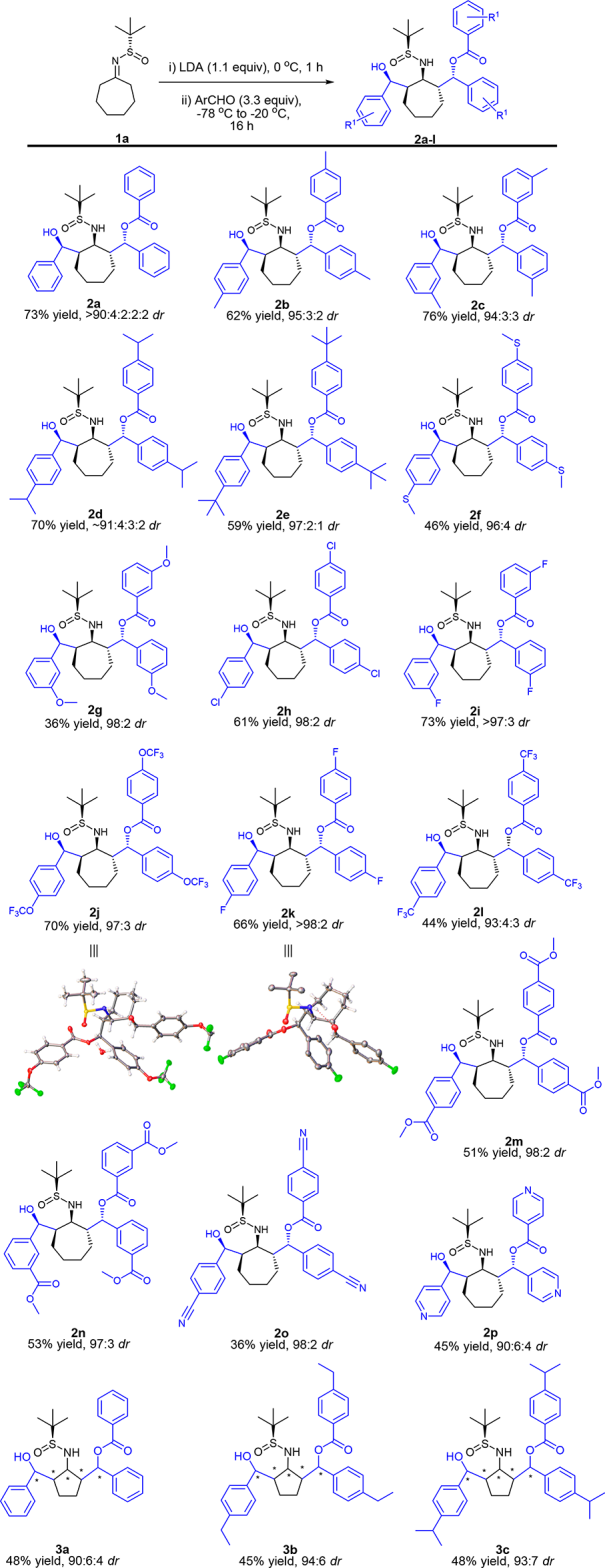


At this stage of our studies, we observed that increasing the molar equivalents of LDA was detrimental to reaction yield. In light of this observation, we hypothesized that a reversible deprotonation step might be a key requirement (known for the single aldol-Tischenko reaction), and that the use of substoichiometric amounts of LDA could actually improve reaction yield. Indeed, we found that the yield of **2a** could be improved by reducing the amount of LDA to 0.8 equiv and warming the reaction mixture to −15 °C for 24 h; these conditions gave an improved yield of 80% (*dr* > 90%, major diastereomer).

Finally, removal of the chiral sulfinyl group could be achieved under mild conditions, revealing the enantioenriched 3-amino-1,5-diol product in high yield and without any loss of diastereomeric purity.^12^

To further test the synthetic utility of this methodology, we applied our reaction conditions to more challenging acyclic substrates using the 2-butanone^13^ derived sulfinimine (**1c**) (Scheme 3). This substrate clearly possesses *two* possible sites for deprotonation: at the kinetically favored methyl carbon and the thermodynamically favored methylene. We were delighted that the reaction of **1c** with benzaldehyde was regioselective for the methylene site and highly diastereoselective (**4a**, >98:2 *dr*, stereochemical orientation undefined). Although a drop in chemical yield was observed for **4a**–**d** compared to previous substrates, high diastereoselectivities were obtained in all cases.

**Scheme 3:**
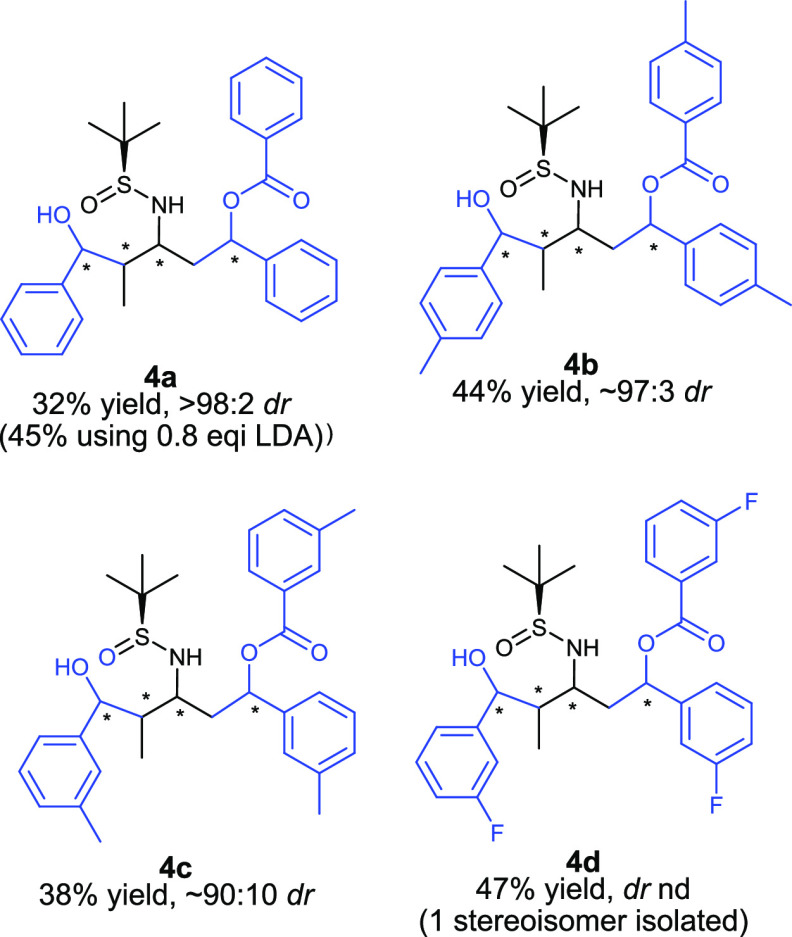


To test for the reversibility of the aldol step, a double aldol product (*prior* to the Tishchenko step) was carefully isolated and treated with 0.8 equiv of LDA at −78 °C. After 1 h, 1.1 equiv of a *different* aldehyde was added and the reaction mixture was warmed to −20 °C (Supporting Information (SI)). If the aldol reaction is truly reversible, then a fully scrambled Tishchenko product would be expected. Indeed, electrospray ionization mass spectrometry (ESI-MS) analysis of the crude reaction mixture showed that a mixture of all the scrambled double aldol–Tishchenko products was present, thus confirming the reversible aldol steps (SI). This experimental result points to reversible aldol and aldol-type steps followed by the key diastereoselective irreversible hydride transfer step.

Furthermore, in one of our double aldol–Tishchenko reactions, attempts to apply our methodology to furfural failed to give the desired product, but rather stalled prior to Tishchenko reduction (**2s**).^14^ Conveniently, this double aldol product provided us with a snapshot of the stereochemistry prior to Tishchenko reaction. The stereochemistry of the major diastereomer was determined to be 2,3-*anti* (by crystallographic analysis), which is “opposite” to the expected double aldol–Tishchenko product, thus providing further evidence that the aldol steps do not contribute to the selectivity of the final products.

Density functional theory (DFT) plays a crucial role in the elucidation of reaction mechanisms,^15^ and advances in DFT provide organic chemists with a powerful tool to predict and rationalize the origins of stereocontrol in asymmetric transformations.^15,16^ Here, density functional theory (DFT) calculations were performed to analyze what factors control the stereoselectivity of the key Tishchenko step using the M06-2X-D3/6-311+G(d,p)//B3LYP-D3/6-31G(d) level of theory. The aldol reaction was first studied^17^ to confirm the reversibility of this step, as determined experimentally, and the description of these results can be found in the SI. Subsequent work focused on the irreversible and stereodetermining intramolecular reduction step of the reaction sequence.

The origins of stereoselectivity for the cycloheptanone derived double aldol–Tishchenko product **2a**, formed in 73% yield and >90:4:2:2:2 *dr*, was examined using DFT methods. In this case, one major diastereomer was observed out of a possible 32. The absolute stereochemistry of the major diastereomer was confirmed by X-ray crystallographic analysis as the (*S*,*R*,*R*,*R*,*S*,*S*)**-**diastereomer. Inclusion of explicit solvent molecules has been shown to be important for calculations of many Li complexes,^16a,17^ and we have previously shown that a disolvated lithium system is an appropriate model for these types of reactions.^6^

Due to the potential formation of 32 diastereomers in the reaction of cycloheptanone sulfinimine **2a** and benzaldehyde, we selected a series of ten representative isomers to study, seven of which are shown in the figures below (see the SI for more details). We were particularly curious about the stereochemical outcome at the C4 and C5 positions, since we had not studied these previously in the reactions that formed two or three chiral centers. Thus, isomers **TS-a**, **TS-b**, **TS-c**, and **TS-d** contain the various possibilities at these positions (Figure 1). In our previous work on a single aldol–Tishchenko reaction to form products with two or three new chiral centers, we showed that it is beneficial for the C1 and C3 stereocenters to be anti to each other, because the formation of these stereoisomers proceeds through a chairlike transition state. We included one isomer, **TS-f** (Figure 2), that proceeds through a twist-boat in order to determine how much higher the TS barrier is for the reactions with cycloheptyl substrates. All other isomers that we calculated contained a C1/C3 anti relationship. We also studied the transition state in which the cycloheptyl ring at C2 is in an equatorial orientation (**TS-e**, Figure 2), and the pseudoenantiomeric isomer in which the *t*-Bu of the sulfinimine and C1 are trans to each other (**TS-f**, Figure 2).

**Figure 1:**
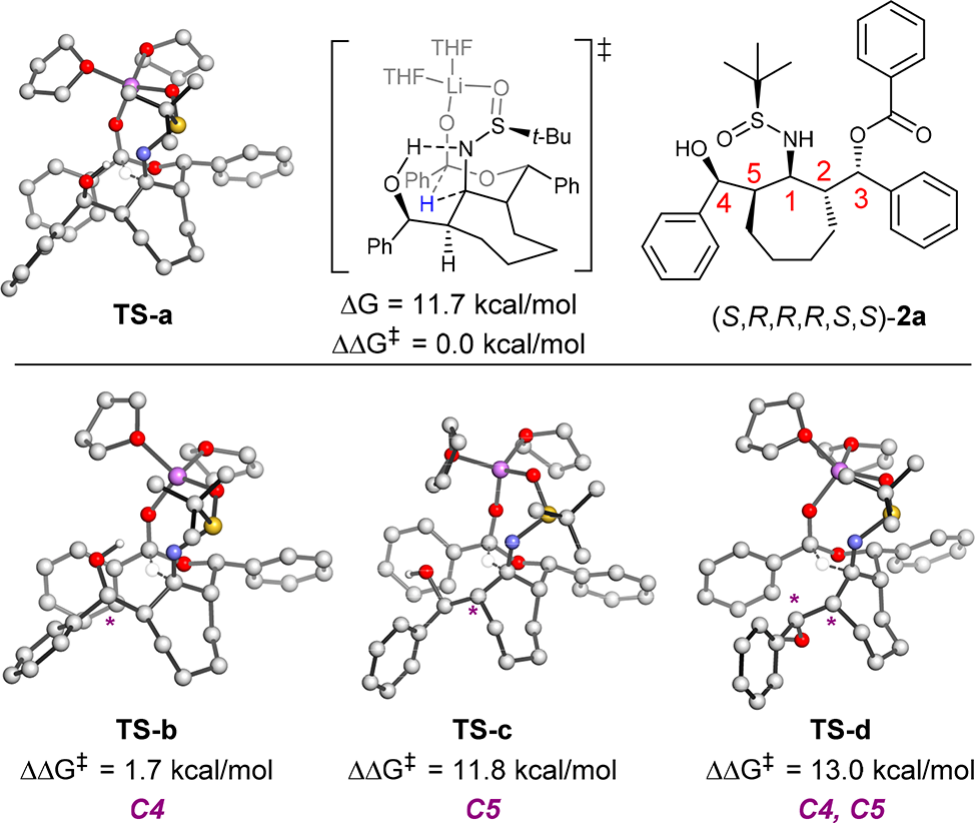


**Figure 2:**
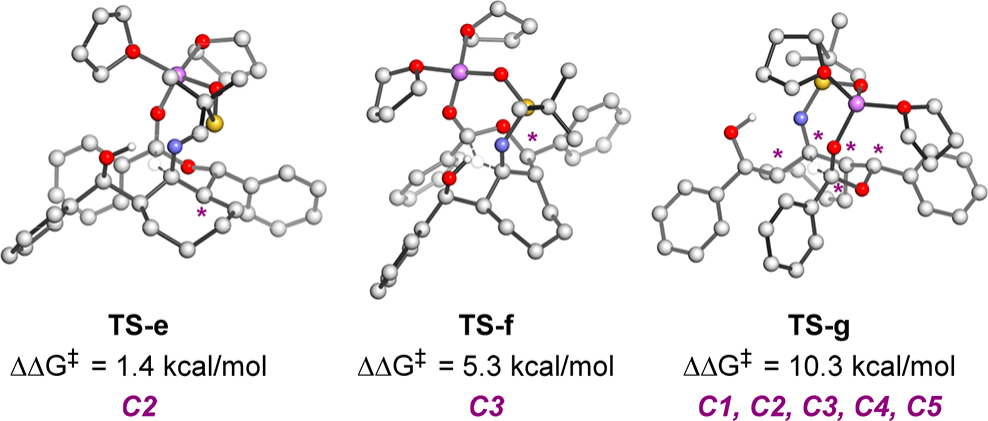


The transition state leading to the major product, **TS-a**, was calculated based on the X-ray crystallographic data obtained for the major diastereomer (Figure 1). The activation energy of the hydride reduction step for **TS-a** was calculated to be 11.7 kcal/mol. The formation of the major diastereomer (*S*,*R*,*R*,*R*,*S*,*S*) proceeds via a six-membered ring in a chair conformation which places the two phenyl substituents in an equatorial position.^17^ The relative stereochemistry at C1 and C3 is *anti*, because this allows for a chairlike transition state in which the Ph group at C3 is equatorial. The stereochemistry at C1 is *syn* to that of the *tert*-butyl group of the sulfinimine. The cycloheptyl ring at C2 is oriented axially in order to avoid unfavorable steric interactions with the *tert*-butyl group of the sulfinimine. The stereochemistry of the alcohol at C4 and the ring at C5 allows for a hydrogen bond with the imine nitrogen, which is distorted in the case of the opposite stereochemistry at C4, and is not possible with the opposite stereochemistry at C5.

Transition states leading to several possible minor diastereomeric products were also calculated. We first examined the transition state leading to the *anti*-(4*R*,5*R*) product (**TS-b**), in which the alcohol is epimeric to the one in **TS-a**. Here, a distorted H-bond also exists, but can only be formed by rotation around the C4–C5 bond that leads to steric interactions between the two Ph rings at C5 and C1. Thus, **TS-b** is 1.7 kcal/mol higher in energy than **TS-a**.

In addition to studying **TS-a** and **TS-b** which differ in the stereochemistry at C4, we were also interested in the stereochemistry at C5. The two possible isomers that are diastereomeric at C4 and C5 are shown in Figure 3 (**TS-c**, **TS-d**). These transition states are significantly higher than **TS-a**, by 11.8 and 13.0 kcal/mol, respectively. These two structures correspond to what would be a pseudoaxial orientation of the ring at C5. However, this pseudoaxial orientation is not possible due to steric constraints, and the H at C5 is forced into an eclipsing interaction with the C–N bond. The C4–C5–C1–N dihedral is 121°–124°, compared to 43° in the case of **TS-a**.

**Figure 3:**
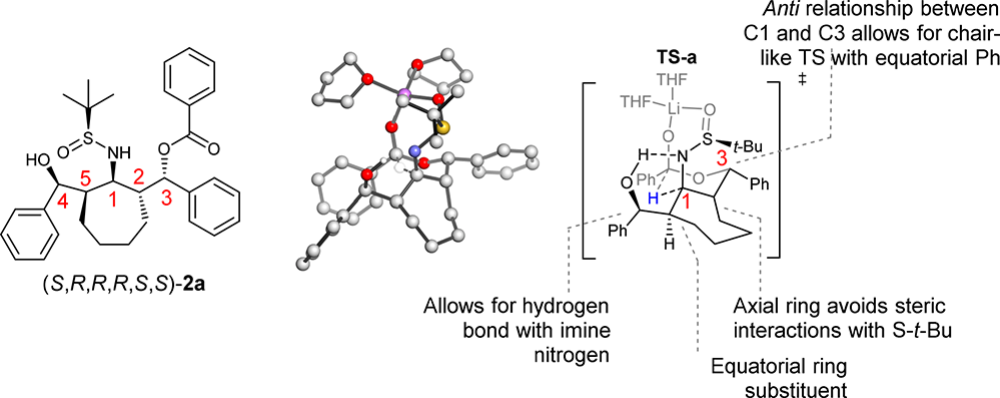


We then considered the structure in which the cycloheptyl ring junction at C2 is in the equatorial orientation (**TS-e**, Figure 2). Not surprisingly, this leads to a steric clash between the *t*-Bu group of the sulfinamide and the cycloheptyl ring, and is disfavored compared to the conformation in which this ring is in the equatorial position by 1.4 kcal/mol.

Next, the transition state leading to the product in which C1 and C3 are *cis* was calculated. In this case, the transition state (**TS-f**) proceeds via a twist-boat conformation in order to avoid an axial orientation of the Ph group at C3. This twist-boat conformation precludes a fully staggered arrangement between the substituents at C2 and C3. A higher activation energy barrier was obtained for this transition state (ΔΔ*G*^‡^ = 5.3 kcal/mol) in comparison to **TS-a**. This is in good agreement with the known energy difference between the twist-boat and chair conformation of cyclohexane (approximately 5 kcal/mol).

In addition, we considered the structures in which the stereochemical relationship between the sulfinamide and the N at C1 is *anti*. One example of this is the pseudoenantiomeric structure, **TS-g**. In this transition state, steric interference between the *t*-Bu group of the sulfinamide and the cycloheptyl ring increases the transition state barrier to 10.3 kcal/mol. In addition to this pseudoenantiomer, the three other diastereomers that differ in the stereochemical outcome at C4 and/or C5 compared to **TS-g** were also studied and showed to also have high transition state barriers (ΔΔ*G*^‡^ between 9.4 and 18.0 kcal/mol; see Supporting Information for details). A summary of the factors affecting stereoselectivity of this reaction is presented in Figure 3.

Based on our mechanistic and computational studies, a plausible reaction mechanism involving a number of reversible steps followed by a highly selective Tishchenko reduction is proposed (see SI for details). An equilibrium effect funnels all aldolate intermediates through the lowest energy transition state (**TS-a**) for the irreversible hydride reduction, leading to the major diastereomer (*S*,*R*,*R*,*R*,*S*,*S*)-**2a**.

In summary, we have developed an efficient one-pot strategy to access 1,3,5-functionalized stereopentads in good yields and excellent diastereoselectivities (>98:2 *dr*). This transformation enabled the synthesis of 5 contiguous stereogenic centers, multiple new chemical bonds, and 3 new functional groups in a single synthetic step. During the key stereodetermining reduction step, DFT studies showed that the cyclic fragment is positioned axially (C2), the relationship between the C1 and C3 stereocenters is *trans*, and the stereochemical orientations at C4 and C5 allow for a hydrogen bond between the OH at C4 and the N at C1.

## Supporting Information Available

General experimental procedures, characterization data, computational data and copies of ^1^H and ^13^C NMR spectra of all key compounds (PDF)

## Accession Codes

CCDC 1877296, 1897008, and 1905742 contain the supplementary crystallographic data for this paper. These data can be obtained free of charge via www.ccdc.cam.ac.uk/data_request/cif, or by emailing data_request@ccdc.cam.ac.uk, or by contacting The Cambridge Crystallographic Data Centre, 12 Union Road, Cambridge CB2 1EZ, UK; fax: +44 1223 336033.
