# Supplementary material for: Stereoselective Installation of Five Contiguous Stereogenic Centers in a Double Aldol–Tishchenko Cascade and Evaluation of the Key Transition State through DFT Calculation
Source: Org Lett. 2021 Aug 10;23(16):6372–6. doi: 10.1021/acs.orglett.1c02179 (PMC8383304; doi:10.1021/acs.orglett.1c02179)
Supplement: Supplementary file 1 — ol1c02179_si_001.pdf [file ol1c02179_si_001.pdf]

Stereoselective Installation of Five Contiguous Stereogenic Centers in a Double Aldol-Tishchenko Cascade and Evaluation of the Key Transition State through DFT Calculation

Pamela Mackey[a], Aneta Turlik[b], Kaori Ando[c], Mark E. Light[d], K. N. Houk[be]* and Gerard P. McGlacken[fa]*

[a,f]School of Chemistry and Analytical and Biological Chemistry Research Facility, University College Cork, Cork, Ireland

[b,e]Department of Chemistry and Biochemistry, University of California, Los Angeles, California 90095-1569, USA

[c]Department of Chemistry and Biomolecular Science, Faculty of Engineering, Gifu University, Yanagido 1-1, Gifu 501-1193, Japan

[d]Mark E. Light, Chemistry Department, University of Southampton, University Road, Southampton SO17 1BJ, United Kingdom

E-mail: g.mcglacken@ucc.ie
houk@chem.ucla.edu

Supporting information

Table of Contents

I. General information………………………………………………………………………….. S3

II. Synthesis and characterisation of (*S*)-*tert*-butyl sulfinimines **1a-d**………………………... S5

III. Synthesis and characterisation of cycloheptanone double aldol-Tishchenko products **2a-p** and double-aldol product **2s**…………………………………………………………………... S8

IV. Synthesis and characterisation of cyclopentanone double aldol-Tishchenko products **3a-c**………………………………………………………………………………………. S25

V. Synthesis and characterisation of 2-butanone double aldol-Tishchenko products **4a-d**…… S28

VI. Regioselective cleavage of *tert*-butyl sulfinyl group………………………………………. S32

VII. High-resolution mass spectra for lithium double-aldolate crossover experiment……….…. S33

VIII. Proposed Mechanism………………………………………………………………………. S36

IX. Tentative assignment of stereochemistry for 2-butanone series…………………………… S37

X. DFT calculations…………………………………………………………………………..... S40

XI. References for supporting information…………………………………………………….. S72

XII. ^{1}H NMR and ^{13}C NMR spectra of novel compounds……………………………………… S73

XIII. Crystallographic data for compounds **2j**, **2k** and **2s**……………………………………….. S98

I. General Information

Solvents were dried and stored over flame dried 4 Å molecular sieves (10-15% w/v) in Young's flask. The concentration of *n*-BuLi in hexanes was determined by titration with diphenylacetic acid. All aldehydes were freshly distilled prior to use and stored under an inert atmosphere. All other reagents were purchased from Sigma-Aldrich, Fluorochem, Alfa Aesar and Acros unless otherwise noted. All non-aqueous reactions were carried out under oxygen-free nitrogen atmosphere using oven dried glassware.

Wet flash column chromatography was carried out using Kieselgel silica gel 60, 0.040-0.063 mm (Merck). Thin layer chromatography (TLC) was carried out on pre-coated silica gel plates (Merck 60 PF254)). Visualisation was achieved by UV and potassium permanganate staining.

Melting points were measured on a Thomas Hoover Capillary Melting Point apparatus.

Infrared (IR) spectra were recorded on a Perkin-Elmer FT-IR Paragon 1000 spectrophotometer.

NMR spectra were run in $CDCl_3$ using TMS as the internal standard at 25 °C. ^1H NMR (600 MHz), ^1H NMR (400 MHz) spectra and ^1H NMR (300 MHz) spectra were recorded on Bruker Avance 600, Bruker Avance 400 and Bruker Avance 300 NMR spectrometers respectively. ^{13}C (150.9 MHz) spectra, ^{13}C (100.6 MHz) spectra and ^{13}C (75.5 MHz) spectra were recorded on Bruker Avance 600, Bruker Avance 400 and Bruker Avance 300 NMR spectrometers respectively in proton decoupled mode. All spectra were recorded at University College Cork. Chemical shifts δ_H and δ_C are expressed as parts per million (ppm), positive shift being downfield from TMS; coupling constants (J) are expressed in hertz (Hz). Splitting patterns in ^1H NMR spectra are designated as s (singlet), *br* s (broad singlet), d (doublet), dd (doublet of doublets), dt (doublet of triplets), t (triplet), q (quartet), quin (quintet), sext (sextet), sept (septet), and m (multiplet). For ^{13}C NMR spectra, the number of attached protons for each signal was determined using the DEPT pulse sequence run in the DEPT-90 and DEPT-135 modes. COSY, HSQC and HMBC experiments were performed to aid the NMR assignment of novel chemical structures.

Low-resolution mass spectra were recorded on a Waters Quattro Micro triple quadrupole instrument in electrospray ionisation (ESI) mode using 50% acetonitrile-water, containing 0.1% formic acid as the mobile phase. Samples were made up in acetonitrile at a concentration of *ca*. 1mg/mL. High-resolution mass spectra were recorded on a Waters LCT Premier TOF LC-MS instrument in electrospray ionisation (ESI) mode using 50% acetonitrile-water, containing 0.1% formic acid as the mobile phase. Samples were made up in acetonitrile at a concentration of *ca*. 1 mg/mL.

Optical rotations were recorded on a DigiPol 781 TDV Polarimeter at 589nm or on an Autopol V Plus Automatic Polarimeter at 589 nm in a 10 cm cell, Concentrations (c) are expressed in g/100ml, $[\alpha]_D^T$ is the specific rotation of a compound and is expressed in units of 10^{-1} deg cm^2 g^{-1}. The specific rotations

were recorded to indicate the direction of enantioselection and optically active samples are numbered with either (+)- or (-)- as prefix.

Single crystal X-ray data was collected at the University of Southampton using a Rigaku AFC12 FRE-HF diffractometer equipped with an Oxford Cryosystems low-temperature device, operating at T = 100 K. The structure was solved with the ShelXT (Sheldrick, 2015) structure solution program using the Intrinsic Phasing solution method and by using Olex2 as the graphical interface. The model was refined with version 2016/6 of ShelXL (Sheldrick, 2015) using Least Squares minimisation. Most hydrogen atom positions were calculated geometrically and refined using the riding model, but some hydrogen atoms were refined freely.

^{1}H NMR spectra, ^{13}C NMR spectra, LRMS and IR analyses were recorded for all previously prepared compounds. For novel compounds, in addition to the previously mentioned analysis, HRMS was also obtained.

In most cases, it was possible to separate the diastereomers, however, the yield reflects the mixture of diastereomers. The major diastereomer was fully characterised in all cases. Diastereoselectivity was determined by analysis of the ^{1}H NMR spectrum of the crude reaction mixture.

II. Synthesis and Characterisation of (*S*)-*tert*-butyl sulfinimines 1a-d

Procedure for cyclic (*S*)-*tert*-butyl sulfinimine, 1a

(*S*,*E*)-2-Methyl-*N*-(cycloheptylidene)propane-2-sulfinamide, 1a

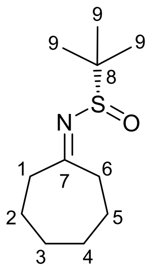

Prepared according to a procedure by Stockman.[1] To a solution of **titanium ethoxide** (6.29 mL, 30 mmol) in anhydrous THF (40 mL) was added **cycloheptanone** (1.30 mL, 11 mmol). The resulting mixture was allowed to stir for 15 min followed by the addition of **(*S*)-*tert*-butanesulfinamide** (1.21 g, 10 mmol). The mixture was stirred at 65 °C in an oil bath and the reaction progress was monitored by TLC analysis. Once the reaction had gone to completion brine (4 mL per mmol of ketone) was added and allowed to stir vigorously for 30 min. The slurry was then filtered through a pad of Celite® and thoroughly washed with EtOAc. The organic layer was dried over anhydrous $MgSO_4$, filtered and concentrated under reduced pressure to afford the crude (*S*)-*tert*-butyl sulfinimine. The crude compound was purified using column chromatography on silica gel (3:1, hexane:EtOAc) to give the title compound **1a** as a yellow oil (1.79 g, 83%).

Spectroscopic characteristics were consistent with previously reported data.[1]

$[\alpha]_D^{22}$ + 158.30 (c 1.0, $CHCl_3$). (lit.[1] $[\alpha]_D^{22}$ + 173.3 (c 0.1, $CHCl_3$) for *S*-enantiomer.) IR v_{max} (NaCl): 1606 (C=N stretch), 1074 (S=O stretch) cm^{-1}. ^{1}H NMR (300 MHz, $CDCl_3$) δ 2.94-3.12 (1H, m, one of H-1/H-6), 2.71-2.89 (1H, m, one of H-1/H-6), 2.53-2.69 (2H, m, one of each H-1 and H-6), 1.47-1.82 (8H, m, H-2, H-3, H-4 and H-5), 1.24 (9H, s, H-9). ^{13}C NMR (75.5 MHz, $CDCl_3$) δ 192.0 (C-7), 56.1 (C-8), 42.3 (C-1 and C-6), 36.5, 29.9 (C-2, C-3, C-4 and C-5), 29.8, 26.4, 25.4, 22.2 (C-9). MS (ESI) *m*/*z*: 216 $(M + H)^+$.

General procedure for the synthesis of (*S*)-*tert*-butyl sulfinimines, 1b and 1c

To a solution of **titanium ethoxide** (2.0 equiv) in THF (4 mL per mmol of ketone) was added **ketone** (1.0 equiv) and **(*S*)-*tert*-butanesulfinamide** (1.0 equiv). The resulting mixture was stirred at reflux in an oil bath and the reaction progress was monitored by TLC analysis. Once the reaction had gone to completion brine (4 mL per mmol of ketone) was added and allowed to stir vigorously for 30 min. The slurry was then filtered through a pad of Celite® and thoroughly washed with Et_2O. The organic layers were combined, dried over anhydrous $MgSO_4$, filtered and concentrated under reduced pressure to

afford the crude (*S*)-*tert*-butyl sulfinimine which was purified using column chromatography on silica gel.

(*S*,*E*)-2-Methyl-*N*-(cyclopentylidene)propane-2-sulfinamide, 1b

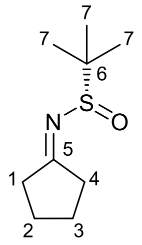

Compound **1b** was prepared using **cyclopentanone** (0.89 mL, 10 mmol) and **(*S*)-*tert*-butanesulfinamide** (1.21 g, 10 mmol). The crude compound was purified using column chromatography on silica gel (3:1, hexane:EtOAc) to give the title compound **1b** as a yellow oil (1.49 g, 80%).

Spectroscopic characteristics were consistent with previously reported data.[2]

$[\alpha]_D^{26}$ + 215.85 (c 1.0, CH_2Cl_2) (lit.[2] $[\alpha]_D^{26}$ - 241.3 (c 1.3, $CHCl_3$) for *R*-enantiomer). IR ν_{max} (NaCl): 1635 (C=N stretch), 1079 (S=O stretch) cm^{-1}. ^1H NMR (300 MHz, $CDCl_3$) δ 2.81-2.99 (1H, dt, *J* = 19.2, 7.8 Hz, one of H-1/H-4), 2.54-2.61 (1H, m, one of H-1/H-4), 2.52 (2H, t, *J* = 7.5 Hz, one of each H-1 and H-4), 1.72-2.02 (4H, m, H-2 and H-3), 1.24 (9H, s, H-7). ^{13}C NMR (75.5 MHz, $CDCl_3$) δ 195.0 (C-5), 56.3 (C-6), 38.9 (C-1 and C-4), 33.9, 25.7 (C-2 and C-3), 23.6, 22.2 (C-7). MS (ESI) *m*/*z*: 188 $(M + H)^+$.

(*S*,*E*)-*N*-(Butan-2-ylidene)-2-methylpropane-2-sulfinamide, 1c

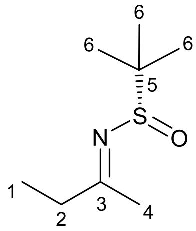

Compound **1c** was prepared using **2-butanone** (0.90 mL, 10 mmol) and **(*S*)-*tert*-butanesulfinamide** (1.21 g, 10 mmol). The crude compound was purified using column chromatography on silica gel (3:1, hexane:EtOAc) to give the title compound **1c** as a pale yellow oil (0.725 g, 41%).

Spectroscopic characteristics were consistent with previously reported data.[3]

$[\alpha]_D^{20}$ + 109.42 (c 1.2, $CHCl_3$) (lit.[3] $[\alpha]_D^{20}$ + 98 (c 1.2, CH_2Cl_2) for *S*-enantiomer). IR ν_{max} (NaCl): 1624 (C=N stretch), 1073 (S=O stretch) cm^{-1}. ^1H NMR (300 MHz, $CDCl_3$) δ 2.43 (2 × 1H, q, *J* = 7.3 Hz, H-2), 2.42, 2.31 (3H, s, H-4), 1.23 (9H, s, H-6), 1.00 (3H, t, *J* = 7.3 Hz, H-1) . ^{13}C NMR (75.5 MHz, $CDCl_3$) δ 186.2 (C-3), 56.4 (C-5), 36.8 (C-2), 22.9 (C-4), 22.3 (C-6), 10.0 (C-1). MS (ESI) *m*/*z*: 176 $(M + H)^+$.

Procedure for cyclic (*S*)-*tert*-butanesulfinyl imine, 1d

(*S*)-*N*-Cyclohexylidene-2-methylpropane-2-sulfinamide, 1d

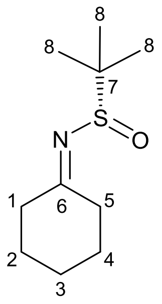

Prepared according to a procedure by Ellman.[4] To a solution of **titanium ethoxide** (8.5 mmol, 1.78 mL) in anhydrous THF (9 mL) was added **cyclohexanone** (5 mmol, 0.52 mL). The resulting mixture was allowed to stir for 15 min followed by the addition of **(*S*)-*tert*-butanesulfinamide** (6 mmol, 0.727 g). The mixture was stirred at 60 ^{o}C in an oil bath and the reaction progress was monitored by TLC analysis. Once the reaction had gone to completion brine (4 mL per mmol of ketone) was added and the solution was allowed to stir vigorously for 30 min. The slurry was then filtered through a pad of Celite® and thoroughly washed with EtOAc. The organic layer was dried over anhydrous $MgSO_4$, filtered and concentrated under reduced pressure to afford the crude (*S*)-*tert*-butyl sulfinimine. The crude compound was purified using column chromatography on silica gel (6:1, Et_2O:EtOAc) to give the title compound **1d** as a colourless oil (0.562 g, 56%).

Spectroscopic characteristics are consistent with previously reported data.[4]

$[\alpha]_D^{23}$ + 74.13 (c 0.325, $CHCl_3$). (lit.[4] $[\alpha]_D^{23}$ - 184.0 (c 1.0, $CHCl_3$) for *R*-enantiomer). IR v_{max} (NaCl): 1614 (C=N stretch), 1072 (S=O stretch) cm^{-1}. ^{1}H NMR (300 MHz, $CDCl_3$) δ 2.81-2.98 (1H, m, one of H-1/H-5), 2.65-2.80 (1H, m, one of H-1/H-5), 2.43 (2H, t, *J* = 6.3 Hz, one of each H-1 and H-5), 1.61-1.88 (6H, m, H-2, H-3 and H-4), 1.23 (9H, s, H-8). ^{13}C NMR (75.5 MHz, $CDCl_3$) δ 188.7 (C-6), 56.0 (C-7), 40.7 (C-1 and C-5), 34.5 (C-2, C-3 and C-4), 28.0, 27.5, 22.2 (C-8). MS (ESI) *m/z*: 202 (M + H)$^+$.

III. Synthesis and characterisation of cycloheptanone double aldol-Tishchenko products 2a-p and double-aldol product 2s

General procedure for the synthesis of cycloheptanone 3-amino-1,5-diol derivatives

To a Schlenk tube under N_2 atmosphere, containing diisopropylamine (1.2 equiv) in anhydrous THF (5 mL per mmol of sulfinimine), was added *n*-BuLi (1.1 equiv) at 0 °C. The mixture was allowed to stir at 0 °C for 20 min to generate a solution of LDA. ***Tert*-butanesulfinimine** (1.0 equiv) was carefully weighed out and pre-dissolved in 1.0 mL of dry THF. The solution of **sulfinimine** was then added slowly to the LDA solution at 0 °C. After the reaction mixture was allowed to stir for 1 h at 0 °C, the solution was cooled to -78 °C and freshly distilled **aldehyde** (3.3 equiv) was added slowly (neat), dropwise. All solid aldehydes were pre-dissolved in dry THF. The reaction mixture was kept at -78 °C for 3 h and was allowed warm to -20 °C over 16 h.

Work-up conditions as per 1 mmol of sulfinimine

The reaction was quenched with sat. aq. NH_4Cl solution (1.5 mL). Sat. aq. NH_4Cl (10 mL) was added and the mixture was extracted with EtOAc (3 × 20 mL). The organic layers were combined, dried over anhydrous $MgSO_4$, filtered and concentrated under reduced pressure to afford the crude product which was purified using column chromatography on silica gel.

(*R*)-((1*R*,2*R*,3*R*)-2-(((*S*)-*Tert*-butylsulfinyl)amino)-3-((*S*)-hydroxy(phenyl)methyl)cycloheptyl)(phenyl)methyl benzoate, 2a

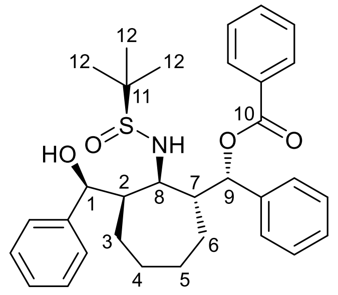

Compound **2a** was prepared using sulfinimine **1a** (0.215 g, 1 mmol) and benzaldehyde (0.34 mL, 3.3 mmol). The crude compound (>90:4:2:2:2 *dr*) was purified using column chromatography on silica gel (2:1, hexane:EtOAc) to give the title compound **2a** as a sticky colourless oil (0.390 g, 73% mixture of diastereomers).

Major diastereomer: $[\alpha]_D^{20}$ + 81.60 (c 0.25, $CHCl_3$). IR ν_{max} (NaCl): 2928 (C-H stretch), 1721 (C=O stretch), 1268 (C-O stretch), 1107 (C-N stretch), 1042 (S=O stretch) cm^{-1}. ^1H NMR (300 MHz, $CDCl_3$) δ 8.12-8.20 (2H, m, Ar-H), 7.57-7.66 (1H, m, Ar-H), 7.46-7.55 (2H, m, Ar-H), 7.15-7.41 (10H, m, Ar-H), 6.26 (1H, d, *J* = 4.3 Hz, H-9), 4.94 (1H, d, *J* = 5.2 Hz, H-1), 4.58 (1H, d, *J* = 5.7 Hz, N-H), 4.23 (1H, d, *J* = 5.4 Hz, O-H), 3.75-3.88 (1H, m, H-8), 2.19-2.33 (1H, m, H-7), 2.00-2.09 (1H, m, H-2),

1.78-1.93 (1H, m, one of H-6), 1.52-1.77 (4H, m, one of each H-3, H-4, H-5 and H-6), 1.33 (9H, s, H-12), 0.96-1.51 (3H, m, one of each H-3, H-4 and H-5). ^{13}C NMR (75.5 MHz, $CDCl_3$) δ 165.7 (C-10), 144.3 (Ar-C), 139.6 (Ar-C), 133.4 (Ar-CH), 130.4 (Ar-CH), 129.9 (2 × Ar-CH), 128.7 (2 × Ar-CH), 128.1 (2 × Ar-CH), 127.9 (2 × Ar-CH), 126.7 (Ar-CH), 126.1 (2 × Ar-CH), 125.8 (2 ×Ar-CH), 76.9 (C-9), 76.4 (C-1), 62.1 (C-8), 56.4 (C-11), 52.8 (C-7), 47.5 (C-2), 29.6 (C-4 and C-5), 27.5, 25.1 (C-6), 23.1 (C-12), 20.9 (C-3). HRMS (ESI) *m*/*z* calcd for $C_{32}H_{39}NO_4SNa$ $[M + Na]^+$: 556.2492, found 556.2494.

Note: Utilising 0.8 equivalents of LDA and warming the reaction mixture to -15 °C over a period of 24 h afforded **2a** in an improved yield of 80%.

(*R*)-((1*R*,2*R*,3*R*)-2-(((*S*)-*Tert*-butylsulfinyl)amino)-3-((*S*)-hydroxy(*p*-tolyl)methyl)cycloheptyl)(*p*-tolyl)methyl 4-methylbenzoate, 2b

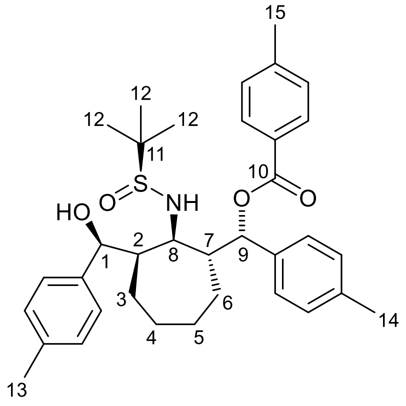

Compound **2b** was prepared using sulfinimine **1a** (0.215 g, 1.0 mmol) and *p*-tolualdehyde (0.39 mL, 3.3 mmol). The crude compound (95:3:2 *dr*) was purified using column chromatography on silica gel (2:1, hexane:EtOAc) to give the title compound **2b** as a sticky yellow oil (0.355 g, 62% mixture of diastereomers).

Major diastereomer: $[\alpha]_D^{20}$ + 93.2 (c 0.25, $CHCl_3$). IR ν_{max} (NaCl): 3138 (O-H stretch), 2925 (C-H stretch), 1719 (C=O stretch), 1270 (C-O stretch), 1178 (C-O stretch), 1102 (C-N stretch), 1043 (S=O stretch) cm^{-1}. ^1H NMR (300 MHz, $CDCl_3$) δ 7.99-8.08 (2H, m, Ar-H), 7.01-8.08 (10H, m, Ar-H), 6.16 (1H, d, , 4.87 (1H, s, H-1), 4.61 (1H, d, *J* = 5.1 Hz, N-H), 4.16 (1H, *br* s, O-H), 3.74-3.85 (1H, m, H-8), 2.43 (3 × 3H, s, H-13, H-14 and H-15), 2.33, 2.31, 2.23-2.38 (1H, m, H-7), 1.92-2.02 (1H, m, H-2), 1.78-1.91 (1H, m, one of H-6), 1.38-1.76 (5H, m, H-3 and one of each H-4, H-5 and H-6), 1.31 (9H, s, H-1), 0.98-1.13 (2H, m, one of each H-4 and H-5). ^{13}C NMR (75.5 MHz, $CDCl_3$) δ 165.8 (C-10), 143.9 (Ar-C), 141.3 (Ar-C), 137.6 (Ar-C), 136.6 (Ar-C), 136.1 (Ar-C), 130.0 (2 × Ar-CH), 129.4 (2 × Ar-CH), 129.3 (2 × Ar-CH), 128.8 (2 × Ar-CH), 127.7 (Ar-C), 126.1 (2 × Ar-CH), 125.6 (2 × Ar-CH), 76.7 (C-9), 76.5 (C-1), 61.8 (C-8), 56.2 (C-11), 52.3 (C-7), 47.3 (C-2), 29.4 (C-4 and C-5), 27.1, 25.4 (C-6), 23.0 (C-12), 21.8 (C-13, C-14 and C-15), 21.2, 20.9 (C-3). HRMS (ESI) *m*/*z* calcd for $C_{35}H_{44}NO_4S$ $[M - H]^-$: 574.2991, found 574.2988.

(*R*)-((1*R*,2*R*,3*R*)-2-(((*S*)-*Tert*-butylsulfinyl)amino)-3-((*S*)-hydroxy(m-tolyl)methyl)cycloheptyl)(m-tolyl)methyl 3-methylbenzoate, 2c

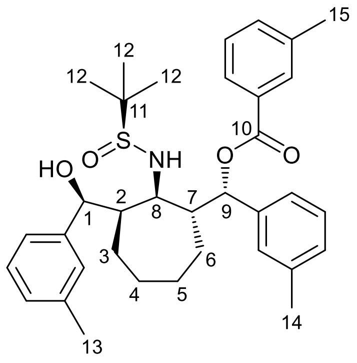

Compound **2c** was prepared using sulfinimine **1a** (0.215 g, 1.0 mmol) and *m*-tolualdehyde (0.39 mL, 3.3 mmol). The crude compound (94:3:3 *dr*) was purified using column chromatography on silica gel (2:1, hexane:EtOAc) to give the title compound **2c** as a white foamy solid (0.435 g, 76% mixture of diastereomers).

Major diastereomer: Mp 81-86 °C. $[\alpha]_D^{20}$ + 85.20 (c 0.25, $CHCl_3$). IR ν_{max} (NaCl): 3327 (O-H stretch), 2926 (C-H stretch), 1718 (C=O stretch), 1275 (C-O stretch), 1105 (C-N stretch), 1042 (S=O stretch) cm^{-1}. ^1H NMR (300 MHz, $CDCl_3$) δ 7.92-7.99 (2H, m, Ar-H), 7.33-7.45 (2H, m, Ar-H), 7.22-7.30 (1H, m, Ar-H), 7.07-7.20 (4H, m, Ar-H), 6.96-7.06 (3H, m, Ar-H), 6.18 (1H, d, *J* = 4.7 Hz, H-9), 4.87 (1H, d, *J* = 5.3 Hz, H-1), 4.59 (1H, d, *J* = 5.3 Hz, N-H), 4.17 (1H, d, *J* = 5.5 Hz, O-H), 3.77-3.85 (1H, m, H-8), 2.24-2.41 (1H, m, H-7), 2.44 (3 × 3H, s, H-13, H-14 and H-15), 1.94-2.03 (1H, m, H-2), 2.32, 2.36, 1.79-1.93 (1H, m, one of H-6), 1.39-1.77 (5H, m, H-3 and one of each H-4, H-5 and H-6), 1.32 (9H, s, H-12), 1.01-1.35 (2H, m, one of each H-4 and H-5). ^{13}C NMR (75.5 MHz, $CDCl_3$) δ 165.9 (C-10), 144.3 (Ar-C), 139.5 (Ar-C), 138.5 (Ar-C), 138.3 (Ar-C), 137.6 (Ar-C), 134.1 (Ar-CH), 130.5 (Ar-CH), 130.3 (Ar-C), 128.8 (Ar-CH), 128.7 (Ar-CH), 128.6 (Ar-CH), 128.0 (Ar-CH), 127.4 (Ar-CH), 127.0 (2 × Ar-CH), 126.4 (Ar-CH), 123.3 (Ar-CH), 122.8 (Ar-CH), 76.9 (C-9), 76.7 (C-1), 61.8 (C-8), 56.3 (C-11), 52.3 (C-7), 47.2 (C-2), 29.4 (C-4 and C-5), 27.1, 25.4 (C-6), 23.1 (C-12), 21.7 (overlapping C-13, C-14 and C-15), 21.5, 20.8 (C-3). HRMS (ESI) *m*/*z* calcd for $C_{35}H_{46}NO_4S$ $[M + H]^+$: 576.3148, found 576.3147.

(*R*)-((1*R*,2*R*,3*R*)-2-(((*S*)-*Tert*-butylsulfinyl)amino)-3-((*S*)-hydroxy(4-isopropylphenyl)methyl)cycloheptyl)(4-isopropylphenyl)methyl 4-isopropylbenzoate, 2d

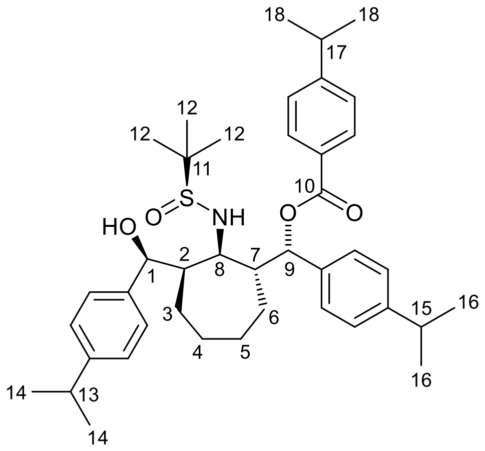

Compound **2d** was prepared using sulfinimine **1a** (0.215 g, 1.0 mmol) and *p-iso*-propyl benzaldehyde (0.50 mL, 3.3 mmol). The crude compound (~91:4:3:2 *dr*) was purified using column chromatography on silica gel (2:1, hexane:EtOAc) to give the title compound **2d** as a pale yellow foamy solid (0.463 g, 70% mixture of diastereomers).

Major diastereomer: Mp 83-87 °C. $[\alpha]_D^{20}$ + 82.40 (c 0.25, $CHCl_3$). IR v_{max} (NaCl): 3226 (O-H stretch), 2960 (C-H stretch), 1719 (C=O stretch), 1270 (C-O stretch), 1104 (C-N stretch), 1052 (S=O stretch) cm^{-1}. ^1H NMR (300 MHz, $CDCl_3$) δ 8.04-8.13 (2H, m, Ar-H), 7.31-7.39 (2H, m, Ar-H), 7.16-7.30 (4H, m, Ar-H), 7.08-7.15 (4H, m, Ar-H), 6.19 (1H, d, *J* = 4.5 Hz, H-9), 4.88 (1H, d, *J* = 1.0 Hz, H-1), 4.61 (1H, d, *J* = 5.3 Hz, N-H), 4.10 (1H, *br* s, O-H), 3.79-3.89 (1H, m, H-8), 3.00 (3 × 1H, sept, *J* = 6.9 Hz (overlapping 2 × 1H, sept and 1H, sept) H-13, H-15 and H-17), 2.88, 2.24-2.39 (1H, m, H-7), 1.94-2.05 (1H, m, H-2), 1.80-1.93 (1H, m, one of H-6), 1.41-1.79 (5H, m, H-3 and one of each H-4, H-5 and H-6), 1.31 (9H, s, H-12), 1.23, 1.30 (3 × 6H, d, *J* = 6.9 Hz (overlapping 2 × 6H, d and 1 × 6H, d) H-14, H-16 and H-18), 1.00-1.36 (2H, m, one of each H-4 and H-5). ^{13}C NMR (75.5 MHz, $CDCl_3$) δ 165.7 (C-10), 154.7 (Ar-C), 148.4 (Ar-C), 147.1 (Ar-C), 141.7 (Ar-C), 136.9 (Ar-C), 130.2 (2 × Ar-CH), 128.1 (Ar-C), 128.1 (2 × Ar-CH), 126.7 (4 × Ar-CH), 126.2 (2 × Ar-CH), 126.1 (2 × Ar-CH), 125.7 (2 × Ar-CH), 76.7 (C-9), 76.6 (C-1), 61.8 (C-8), 56.3 (C-11), 52.2 (C-7), 47.1 (C-2), 34.5 (C-13, C-15 and C-17), 33.9, 33.8, 29.5 (C-4 and C-5), 27.2, 25.6 (C-11), 24.1 (C-14, C-16 and C-18), 24.0, 23.9, 23.1 (C-12), 21.0 (C-3) HRMS (ESI) *m*/*z* calcd for $C_{41}H_{57}NO_4SNa$ $[M + Na]^+$: 682.3901, found 682.3885.

Note: Only partial separation of diastereomers could be achieved.

(*R*)-(4-(*Tert*-butyl)phenyl)((1*R*,2*R*,3*R*)-3-((*S*)-(4-(*tert*-butyl)phenyl)(hydroxy)methyl)-2-(((*S*)-*tert*-butylsulfinyl)amino)cycloheptyl)methyl 4-(*tert*-butyl)benzoate, 2e

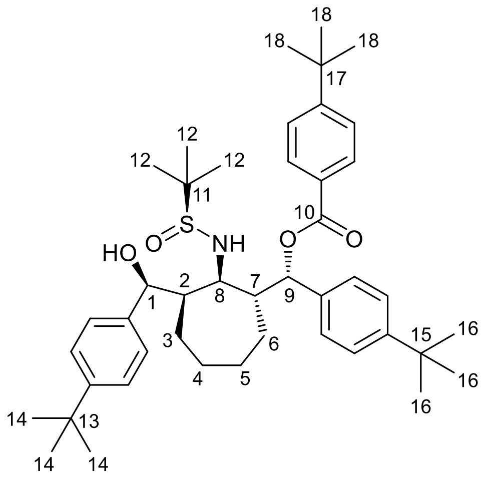

Compound **2e** was prepared using sulfinimine **1a** (0.215 g, 1.0 mmol) and *p*-*tert*-butylbenzaldehyde (0.55 mL, 3.3 mmol). The crude compound (97:2:1 *dr*) was purified using column chromatography on silica gel (2:1, hexane:EtOAc) to give the title compound **2e** as a pale yellow solid (0.414 g, 59% mixture of diastereomers).

Major diastereomer: Mp 109-113 °C. $[\alpha]_D^{20}$ + 62.55 (c 0.275, $CHCl_3$). IR v_{max} (NaCl): 3223 (O-H stretch), 2962 (C-H stretch), 1721 (C=O stretch), 1270 (C-O stretch), 1114 (C-N stretch), 1043 (S=O stretch) cm^{-1}. ^1H NMR (300 MHz, $CDCl_3$) δ 8.07-8.13 (2H, m, Ar-H), 7.47-7.57 (2H, m, Ar-H), 7.19-7.42 (6H, m, Ar-H), 7.06-7.18 (2H, m, Ar-H), 6.20 (1H, d, *J* = 4.4 Hz, H-9), 4.88 (1H, d, *J* = 4.8 Hz, H-1), 4.59 (1H, d, *J* = 5.2 Hz, N-H), 4.12 (1H, d, *J* = 5.3 Hz, O-H), 3.82-3.89 (1H, m, H-8), 2.26-2.40 (1H, m, H-7), 1.97-2.06 (1H, m, H-2), 1.79-1.94 (1H, m, one of H-6), 1.47-1.77 (5H, m, H-3 and one of each H-4, H-5 and H-6), 1.37 (overlapping 4 × 9H, s, H-12, H-14, H-16 and H-18), 1.30, 1.02-1.41 (2H, m, one of each H-4 and H-5). ^{13}C NMR (75.5 MHz, $CDCl_3$) 165.7 (C-10), 156.9 (Ar-C), 150.6 (Ar-C), 149.3 (Ar-C), 141.2 (Ar-C), 136.5 (Ar-C), 129.8 (2 × Ar-CH), 127.6 (Ar-C), 127.0 (2 × Ar-CH), 125.6 (2 × Ar-CH), 125.5 (2 × Ar-CH), 125.4 (2 × Ar-CH), 124.9 (2 × Ar-CH), 76.8 (C-9), 76.6 (C-1), 61.8 (C-8), 56.2 (C-11), 52.0 (C-7), 47.0 (C-2), 35.2 (C-13, C-15 and C-17), 34.6, 34.5, 31.5 (C-14, C-16 and C-18), 31.4, 31.3, 29.5 (C-4 and C-5), 27.1, 25.7 (C-6), 23.0 (C-12), 21.0 (C-3). HRMS (ESI) *m*/*z* calcd for $C_{44}H_{63}NO_4SNa$ $[M + Na]^+$: 724.4370, found 724.4367.

(*R*)-((1*R*,2*R*,3*R*)-2-(((*S*)-*Tert*-butylsulfinyl)amino)-3-((*S*)-hydroxy(4-(methylthio)phenyl)methyl)cycloheptyl)(4-(methylthio)phenyl)methyl 4-(methylthio)benzoate, 2f

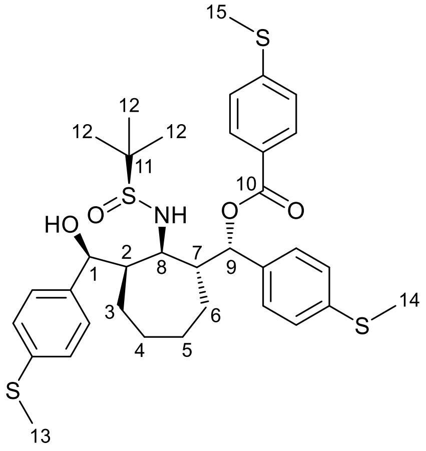

Compound **2f** was prepared using sulfinimine **1a** (0.140 g, 0.65 mmol) and *p*-thiomethoxybenzaldehyde (0.29 mL, 2.15 mmol). The crude compound (96:4 *dr*) was purified using column chromatography on silica gel (1:1, hexane:EtOAc) to give the title compound **2f** as a sticky colourless oil (0.203 g, 46% mixture of diastereomers).

Major diastereomer: $[\alpha]_D^{20}$ + 166.00 (c 0.1, $CHCl_3$). IR v_{max} (NaCl): 3234 (O-H stretch), 2922 (C-H stretch), 1715 (C=O stretch), 1268 (C-O stretch), 1105 (C-N stretch), 1040 (S=O stretch) cm^{-1}. ^1H NMR (300 MHz, $CDCl_3$) δ 8.01-8.11 (2H, m, Ar-H), 7.07-7.41 (10H, m, Ar-H), 6.12 (1H, d, *J* = 4.1 Hz, H-9), 4.90 (1H, d, *J* = 4.4 Hz, H-1), 4.63 (1H, d, *J* = 5.2 Hz, N-H), 4.30 (1H, d, *J* = 5.3 Hz, O-H), 3.76-3.85 (1H, m, H-8), 2.55 (3 × 3H, s (overlapping 2 × 3H, s and 1 × 3H, s) H-13, H-14 and H-15), 2.48, 2.20-2.36 (1H, m, H-7), 1.78-1.98 (2H, m, one of H-6 and H-2), 1.38-1.77 (5H, m, H-3 and one of each H-4, H-5 and H-6), 1.31 (9H, s, H-12), 0.97-1.27 (2H, m, one of each H-4 and H-5). ^{13}C NMR (75.5 MHz, $CDCl_3$) δ 165.4 (C-10), 146.2 (Ar-C), 141.3 (Ar-C), 138.2 (Ar-C), 136.4 (Ar-C), 136.3 (Ar-C), 130.2 (2 × Ar-CH), 126.7 (2 × Ar-CH), 126.6 (2 × Ar-CH), 126.5 (2 × Ar-CH), 126.3 (2 × Ar-CH), 126.2 (Ar-C), 125.2 (2 × Ar-CH), 76.5 (C-9), 76.2 (C-1), 61.7 (C-8), 56.3 (C-11), 52.3 (C-7), 47.3 (C-2), 29.6 (C-4 and C-5), 27.3, 25.5 (C-6), 23.0 (C-12), 20.9 (C-3), 16.1 (C-13, C-14 and C-15), 15.8, 15.0. HRMS (ESI) *m*/*z* calcd for $C_{35}H_{45}NO_4S_4Na$ $[M + Na]^+$: 694.2124, found 694.2121.

(*S*)-((1*R*,2*R*,3*R*)-2-(((*S*)-*Tert*-butylsulfinyl)amino)-3-((*S*)-hydroxy(4-methoxyphenyl)methyl)cycloheptyl)(4-methoxyphenyl)methyl 4-methoxybenzoate, 2g

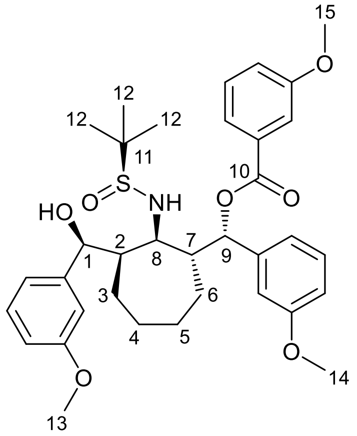

Compound **2g** was prepared using sulfinimine **1a** (0.140 g, 0.65 mmol) and *m*-methoxybenzaldehyde (0.26 mL, 2.15 mmol). The crude compound (98:2 *dr*) was purified using column chromatography on silica gel (1:1, hexane:EtOAc) to give the title compound **2g** as a sticky colourless oil (0.146 g, 36% mixture of diastereomers).

Major diastereomer: $[\alpha]_D^{20}$ + 74.32 (c 0.41, $CHCl_3$). IR v_{max} (NaCl): 3391 (O-H stretch), 2932 (C-H stretch), 1719 (C=O stretch), 1275 (C-O stretch), 1103 (C-N stretch), 1042 (S=O stretch) cm^{-1}. ^{1}H NMR (300 MHz, $CDCl_3$) δ 7.76 (1H, td, *J* = 7.8, 1.3 Hz, Ar-H), 7.65 (1H, dd, *J* = 2.5, 1.5 Hz, Ar-H), 7.36-7.45 (1H, m, Ar-H), 7.24-7.32 (1H, m, Ar-H), 7.12-7.21 (2H, m, Ar-H), 6.86-6.95 (3H, m, Ar-H), 6.76-6.85 (2H, m, Ar-H), 6.70-6.75 (1H, m, Ar-H), 6.21 (1H, d, *J* = 4.0 Hz, H-9), 4.93 (1H, d, *J* = 4.7 Hz, H-1), 4.60 (1H, d, *J* = 5.8 Hz, N-H), 4.33 (1H, d, *J* = 5.5 Hz, O-H), 3.87 (3 × 3H, s, H-13, H-14 and H-15), 3.78, 3.77, 3.74-3.83 (1H, m, H-8), 2.15-2.29 (1H, m, H-7), 2.00-2.09 (1H, m, H-2), 1.79-1.92 (1H, m, one of H-6), 1.45-1.78 (5H, m, H-3 and one of each H-4, H-5 and H-6), 1.33 (9H, s, H-12), 1.03-1.24 (2H, m, one of each H-4 and H-5). ^{13}C NMR (75.5 MHz, $CDCl_3$) δ 165.6 (C-10), 159.8 (2 × Ar-C), 159.6 (Ar-C), 146.1 (Ar-C), 141.3 (Ar-C), 131.5 (Ar-C), 129.8 (Ar-CH), 129.7 (Ar-CH), 129.0 (Ar-CH), 122.2 (Ar-CH), 119.6 (Ar-CH), 118.4 (Ar-CH), 118.1 (Ar-CH), 114.6 (Ar-CH), 112.5 (2 × Ar-CH), 112.2 (Ar-CH), 111.5 (Ar-CH), 76.7 (C-9), 76.1 (C-1), 62.2 (C-8), 56.4 (C-11), 55.6 (overlapping C-13, C-14 and C-15), 55.3, 53.0 (C-7), 47.6 (C-2), 29.6 (C-4 and C-5), 27.8, 25.0 (C-6), 23.1 (C-12), 21.1 (C-3). HRMS (ESI) *m*/*z* calcd for $C_{35}H_{46}NO_7S$ $[M + H]^+$: 624.2990, found 624.2990.

(*R*)-((1*R*,2*R*,3*R*)-2-(((*S*)-*Tert*-butylsulfinyl)amino)-3-((*S*)-(4-chlorophenyl)(hydroxy)methyl)cycloheptyl)(4-chlorophenyl)methyl 4-chlorobenzoate, 2h

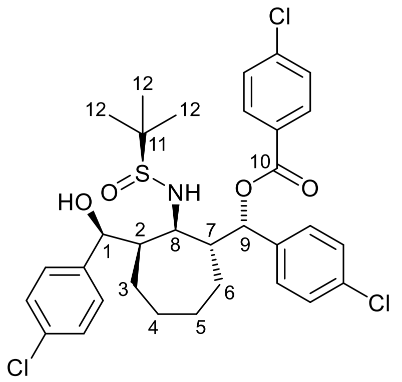

Compound **2h** was prepared using sulfinimine **1a** (0.215 g, 1.0 mmol) and *p*-chlorobenzaldehyde (0.464 g, 3.3 mmol). The crude compound (98:2 *dr*) was purified using column chromatography on silica gel (2:1, hexane:EtOAc) to give the title compound **2h** as a sticky colourless oil (0.391 g, 61% mixture of diastereomers).

Major diastereomer: $[\alpha]_D^{20}$ + 124.00 (c 0.25, $CHCl_3$). IR v_{max} (NaCl): 3228 (O-H stretch), 2928 (C-H stretch), 1722 (C=O stretch), 1268 (C-O stretch), 1092 (C-N stretch), 1041 (S=O stretch) cm^{-1}. ^1H NMR (300 MHz, $CDCl_3$) δ 8.02-8.13 (2H, m, Ar-H), 7.43-7.52 (2H, m, Ar-H), 7.19-7.38 (6H, m, Ar-H), 7.09-7.17 (2H, m, Ar-H), 6.18 (1H, d, *J* = 3.6 Hz, H-9), 4.89 (1H, d, *J* = 5.4 Hz, H-1), 4.62 (1H, d, *J* = 5.6 Hz, N-H), 4.43 (1H, d, *J* = 5.6 Hz, O-H), 3.70-3.81 (1H, m, H-8), 2.14-2.26 (1H, m, H-7), 1.77-1.92 (2H, m, one of H-6 and H-2), 1.36-1.76 (5H, m, H-3 and one of each H-4, H-5 and H-6), 1.32 (9H, s, H-12), 0.82-1.23 (2H, m, one of each H-4 and H-5). ^{13}C NMR (75.5 MHz, $CDCl_3$) δ 164.8 (C-10), 142.7 (Ar-C), 140.1 (Ar-C), 137.9 (Ar-C), 133.9 (Ar-C), 132.5 (Ar-C), 131.3 (2 × Ar-CH), 129.1 (2 × Ar-CH), 129.0 (2 × Ar-CH), 128.4 (Ar-C), 128.3 (2 × Ar-CH), 127.4 (2 × Ar-CH), 127.2 (2 × Ar-CH), 76.4 (C-9), 75.7 (C-1), 61.7 (C-8), 56.6 (C-11), 52.7 (C-7), 47.6 (C-2), 29.7 (C-4 and C-5), 27.7 25.3 (C-6), 23.1 (C-12), 20.9 (C-3). HRMS (ESI) *m*/*z* calcd for $C_{32}H_{36}Cl_3NO_4SNa$ $[M + Na]^+$: 658.1322, found 658.1311.

(*R*)-((1*R*,2*R*,3*R*)-2-(((*S*)-*Tert*-butylsulfinyl)amino)-3-((*S*)-(3-fluorophenyl)(hydroxy)methyl)cycloheptyl)(3-fluorophenyl)methyl 3-fluorobenzoate, 2i

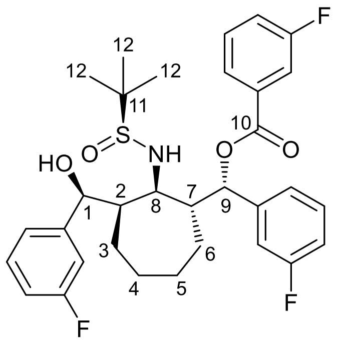

Compound **2i** was prepared using sulfinimine **1a** (0.215 g, 1.0 mmol) and *m*-fluorobenzaldehyde (0.35 mL, 3.3 mmol). The crude compound (>97:3 *dr*) was purified using column chromatography on silica gel (2:1, hexane:EtOAc) to give the title compound **2i** as a white foamy solid (0.431 g, 73% mixture of diastereomers).

Major diastereomer: Mp 77-82 °C. $[\alpha]_D^{20}$ + 76.92 (c 0.26, $CHCl_3$). IR ν_{max} (NaCl): 3139 (O-H stretch), 2931 (C-H stretch), 1727 (C=O stretch), 1270 (C-O stretch), 1042 (S=O stretch) cm^{-1}. ^1H NMR (300 MHz, $CDCl_3$) δ 7.95 (1H, dt, *J* = 7.7, 1.2 Hz, Ar-H), 7.82 (1H, ddd, *J* = 9.2, 2.6, 1.5 Hz, Ar-H), 7.49 (1H, dt, *J* = 8.1, 5.6 Hz, Ar-H), 7.29-7.37 (2H, m, Ar-H), 7.18-7.25 (1H, m, Ar-H), 7.06-7.11 (1H, m, Ar-H), 6.93-7.05 (4H, m, Ar-H), 6.82- 6.92 (1H, m, Ar-H), 6.25 (1H, d, *J* = 3.4 Hz, H-9), 4.96 (1H, d, *J* = 5.6 Hz, H-1), 4.63 (1H, d, *J* = 6.0 Hz, N-H), 4.56 (1H, d, *J* = 5.7 Hz, O-H), 3.70-3.81 (1H, m, H-8), 2.12-2.23 (1H, m, H-7), 1.96-2.06 (1H, m, H-2), 1.39-1.87 (6H, m, H-3 and H-6 and one of each H-4 and H-5), 1.34 (9H, s, H-12), 0.93-1.25 (2H, m, one of each H-4 and H-5) ^{13}C NMR (75.5 MHz, $CDCl_3$) δ 164.5 (C-10), 163.1 (d, $^1J_{C-F}$ = 246.7 Hz, Ar-C), 163.0 (d, $^1J_{C-F}$ = 245 Hz, Ar-C), 162.8 (d, $^1J_{C-F}$ = 250 Hz, Ar-C), 147.2 (d, $^3J_{C-F}$ = 6.9 Hz, Ar-C), 142.2 (d, $^3J_{C-F}$ = 6.9 Hz, Ar-C), 132.0 (d, $^3J_{C-F}$ = 7.4 Hz, Ar-C), 130.5 (d, $^3J_{C-F}$ = 7.8 Hz, Ar-CH), 130.4 (d, $^3J_{C-F}$ = 8.1 Hz, Ar-CH), 129.6 (d, $^3J_{C-F}$ = 8.2 Hz, Ar-CH), 125.7 (d, $^4J_{C-F}$ = 3.0 Hz, Ar-CH), 121.5 (d, $^4J_{C-F}$ = 2.8 Hz, Ar-CH), 121.3 (d, $^4J_{C-F}$ = 2.8 Hz, Ar-CH), 120.7 (d, $^2J_{C-F}$ = 21.2 Hz, Ar-CH), 116.8 (d, $^2J_{C-F}$ = 23.0 Hz, Ar-CH), 114.9 (d, $^2J_{C-F}$ = 21.2 Hz, Ar-CH), 113.5 (d, $^2J_{C-F}$ = 21.2 Hz, Ar-CH), 113.2 (d, $^2J_{C-F}$ = 22.4 Hz, Ar-CH), 112.9 (d, $^2J_{C-F}$ = 22.4 Hz, Ar-CH), 76.4 (C-9), 75.4 (C-1), 62.3 (C-8), 56.6 (C-11), 53.4 (C-7), 47.8 (C-2), 29.7 (C-4 and C-5), 28.2, 24.8 (C-6), 23.1 (C-12), 21.1 (C-3). HRMS (ESI) *m*/*z* calcd for $C_{32}H_{36}F_3NO_4SNa$ $[M + Na]^+$: 610.2209, found 610.2213.

(*R*)-((1*R*,2*R*,3*R*)-2-(((*S*)-*Tert*-butylsulfinyl)amino)-3-((*S*)-hydroxy(4-(trifluoromethoxy)phenyl)methyl)cycloheptyl)(4-(trifluoromethoxy)phenyl)methyl 4-(trifluoromethoxy)benzoate, 2j

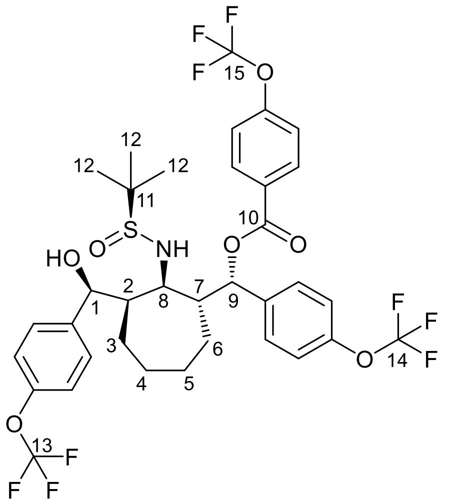

Compound **2j** was prepared using sulfinimine **1a** (0.215 g, 1.0 mmol) and *p*-trifluoromethoxybenzaldehyde (0.47 mL, 3.3 mmol). The crude compound (97:3 *dr*) was purified using column chromatography on silica gel (2:1, hexane:EtOAc) to give the title compound **2j** as a white foamy solid (0.552 g, 70% mixture of diastereomers).

Major diastereomer: Mp 59-64 °C. $[\alpha]_D^{20}$ + 71.60 (c 0.25, $CHCl_3$). IR ν_{max} (NaCl): 3326 (O-H stretch), 2932 (C-H stretch), 1726 (C=O stretch), 1261 (C-O stretch), 1040 (S=O stretch) cm^{-1}. ^1H NMR (300 MHz, $CDCl_3$) δ 8.17-8.24 (2H, m, Ar-H), 7.29-7.39 (4H, m, Ar-H), 7.16-7.28 (4H, m, Ar-H), 7.07-7.15 (2H, m, Ar-H), 6.25 (1H, d, *J* = 3.6 Hz, H-9), 4.96 (1H, d, *J* = 5.2 Hz, H-1), 4.64 (1H, d, *J* = 5.6 Hz, N-H), 4.48 (1H, d, *J* = 5.6 Hz, O-H), 3.74-3.85 (1H, m, H-8), 2.14-2.30 (1H, m, H-7), 1.79-1.97 (2H, m, one of H-6 and H-2), 1.41-1.79 (5H, m, H-3 and one of each H-4, H-5 and H-6), 1.33 (9H, s, H-12), 0.92-1.25 (2H, m, one of each H-4 and H-5). ^{13}C NMR (75.5 MHz, $CDCl_3$) δ 164.5 (C-10), 153.2 (d, $^3J_{C-F}$ = 1.7 Hz, Ar-C), 148.9 (d, $^3J_{C-F}$ = 2.1 Hz, Ar-C), 148.1 (d, $^3J_{C-F}$ = 2.1 Hz, Ar-C), 142.9 (Ar-C), 138.1 (Ar-C), 132.0 (2 × Ar-CH), 128.3 (Ar-C), 127.4 (2 × Ar-CH), 121.2 (2 × Ar-CH), 120.7 (2 × Ar-CH), 120.6 (2 × Ar-CH), 118.6 (q, $^1J_{C-F}$ = 258.8 Hz and overlapping 2 × q, $^1J_{C-F}$ = 257.2, 257.6 Hz, C-13, C-14 and C-15), 118.5, 76.4 (C-9), 75.6 (C-1), 62.0 (C-8), 56.6 (C-11), 53.1 (C-7), 47.7 (C-2), 29.8 (C-4 and C-5), 28.0, 25.2 (C-6), 23.1 (C-12), 21.0 (C-3). HRMS (ESI) *m/z* calcd for $C_{35}H_{36}F_9NO_7SNa$ $[M + Na]^+$: 808.1961, found 808.1962.

Absolute stereochemistry was assigned by crystallographic data.

(*R*)-((1*R*,2*R*,3*R*)-2-(((*S*)-*Tert*-butylsulfinyl)amino)-3-((*S*)-(4-fluorophenyl)(hydroxy)methyl)cycloheptyl)(4-fluorophenyl)methyl 4-fluorobenzoate, 2k

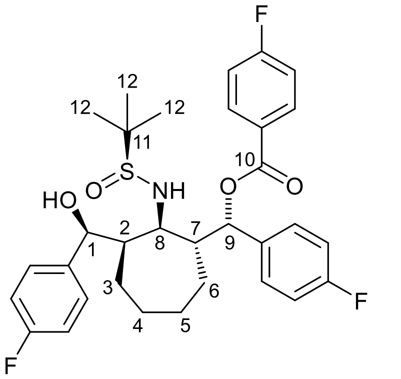

Compound **2k** was prepared using sulfinimine **1a** (0.215 g, 1.0 mmol) and *p*-fluorobenzaldehyde (0.35 mL, 3.3 mmol). The crude compound (>98:2 *dr*) was purified using column chromatography on silica gel (2:1, hexane:EtOAc) to give the title compound **2k** as a white foamy solid (0.386 g, 66% mixture of diastereomers).

Major diastereomer: Mp 71-76 °C. $[\alpha]_D^{20}$ + 90.00 (c 0.25, CHCl$_3$). IR ν_{max} (NaCl): 3222 (O-H stretch), 2931 (C-H stretch), 1723 (C=O stretch), 1268 (C-O stretch), 1090 (C-N stretch), 1040 (S=O stretch) cm^{-1}. ^{1}H NMR (300 MHz, CDCl$_3$) δ 8.01-8.13 (2H, m, Ar-H), 7.16-7.28 (2H, m, Ar-H), 7.04-7.16 (4H, m, Ar-H), 6.81-7.04 (4H, m, Ar-H), 6.12 (1H, d, *J* = 4.3 Hz, H-9), 4.83 (1H, d, *J* = 5.2 Hz, N-H), 4.57 (1H, d, *J* = 5.4 Hz, H-1), 4.27 (1H, d, *J* = 5.5 Hz, O-H), 3.64-3.71 (1H, m, H-8), 2.11-2.22 (1H, m, H-7), 1.71-1.94 (2H, m, H-2 and one of H-6), 1.30-1.69 (5H, m, H-3 and one of each H-4, H-5 and H-6), 1.25 (9H, s, H-12), 0.87-1.28 (2H, m, one of each H-4 and H-5). ^{13}C NMR (75.5 MHz, CDCl$_3$) δ 166.2 (d, $^1J_{C\text{-}F}$ = 254.9 Hz, Ar-C), 164.6 (C-10), 162.4 ($^1J_{C\text{-}F}$ = 246.6 Hz, Ar-C), 161.8 (d, $^1J_{C\text{-}F}$ = 244.9 Hz, Ar-C), 139.9 (d, $^4J_{C\text{-}F}$ = 2.9 Hz, Ar-C), 135.2 (d, $^4J_{C\text{-}F}$ = 2.5 Hz, Ar-C), 132.5 (d, $^3J_{C\text{-}F}$ = 9.4 Hz, 2 × Ar-CH), 127.8 (d, $^3J_{C\text{-}F}$ = 8.0 Hz, 2 × Ar-CH), 127.3 (d, $^3J_{C\text{-}F}$ = 8.0 Hz, 2 × Ar-CH), 126.3 (d, $^4J_{C\text{-}F}$ = 2.8 Hz, Ar-C), 115.9 (d, $^2J_{C\text{-}F}$ = 21.6 Hz, 2 × Ar-CH), 115.8 (d, $^2J_{C\text{-}F}$ = 22.1 Hz, 2 × Ar-CH), 114.9 (d, $^2J_{C\text{-}F}$ = 21.2 Hz, 2 × Ar-CH), 76.4 (C-9), 75.8 (C-1), 61.6 (C-8), 56.5 (C-11), 52.5 (C-7), 47.5 (C-2), 29.6 (C-4 and C-5), 27.4, 25.2 (C-6), 23.1 (C-12), 20.8 (C-8). HRMS (ESI) *m/z* calcd for $C_{32}H_{35}F_3NO_4S$ [M - H]$^-$: 586.2239, found 586.2244.

Absolute stereochemistry was assigned by crystallographic data.

(*S*)-((1*R*,2*R*,3*R*)-2-(((*S*)-*Tert*-butylsulfinyl)amino)-3-((*S*)-hydroxy(4-(trifluoromethyl)phenyl)methyl)cycloheptyl)(4-(trifluoromethyl)phenyl)methyl 4-(trifluoromethyl)benzoate, 2l

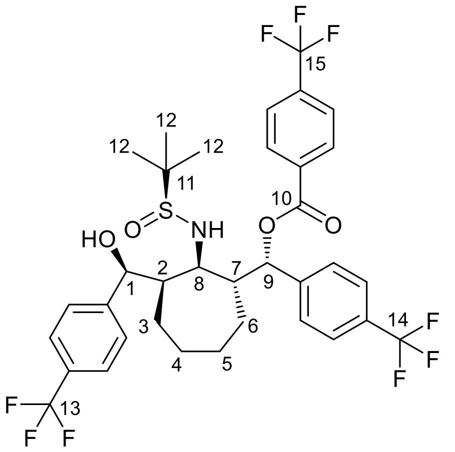

Compound **2l** was prepared using sulfinimine **1a** (0.215 g, 1.0 mmol) and *p*-trifluoromethylbenzaldehyde (0.45 mL, 3.3 mmol). The crude compound (93:4:3 *dr*) was purified using column chromatography on silica gel (4:1, hexane:EtOAc) to give the title compound **2l** as a sticky colourless oil (0.322 g, 44% mixture of diastereomers).

Major diastereomer: $[\alpha]_D^{20}$ + 76.95 (c 0.18, $CHCl_3$). IR ν_{max} (NaCl): 3366 (O-H stretch), 2951 (C-H stretch), 1723 (C=O stretch), 1280 (C-O stretch), 1107 (C-N stretch), 1038 (S=O stretch) cm^{-1}. ^{1}H NMR (300 MHz, $CDCl_3$) δ 8.25-8.33 (2H, m, Ar-H), 7.76-7.85 (2H, m, Ar-H), 7.61-7.69 (2H, m, Ar-H), 7.48-7.56 (2H, m, Ar-H), 7.39-7.47 (2H, m, Ar-H), 7.29-7.37 (2H, m, Ar-H), 6.33 (1H, d, J = 2.7 Hz, H-9), 5.02 (1H, d, J = 5.7 Hz, H-1), 4.67 (1H, d, J = 6.0 Hz, N-H), 4.54 (1H, d, J = 5.8 Hz, O-H), 3.79-3.89 (1H, m, H-8), 2.15-2.29 (1H, m, H-7), 1.92-1.99 (1H, m, H-2), 1.48-1.91 (6H, m, H-3 and H-6 and one of each H-4 and H-5), 1.36 (9H, s, H-12), 0.80-1.22 (2H, m, one of each H-4 and H-5). ^{13}C NMR (75.5 MHz, $CDCl_3$) δ 164.5 (C-10), 148.2 (Ar-C), 143.4 (Ar-C), 135.3 (q, $^2J_{C-F}$ = 32.7 Hz, Ar-C), 133.0 (Ar-C), 130.4 (2 × Ar-CH), 130.3 (q, $^2J_{C-F}$ = 32.2 Hz, Ar-C), 129.1 (q, $^2J_{C-F}$ = 32.9 Hz, Ar-C), 126.2 (2 × Ar-CH), 126.1 (2 × Ar-CH), 125.9 (q, $^3J_{C-F}$ = 3.5 Hz, 4 × Ar-CH), 125.1 (q, $^3J_{C-F}$ = 3.8 Hz, 2 × Ar-CH), 124.3 (q, $^1J_{C-F}$ = 273.1, 272.2, 272.6 Hz, C-13, C-14 and C-15), 124.0, 123.7, 76.7 (C-9), 75.6 (C-1), 62.2 (C-8), 56.8 (C-11), 53.5 (C-7), 47.8 (C-2), 29.9 (C-4 and C-5), 28.4, 25.1 (C-6), 23.1 (C-12), 21.1 (C-3). HRMS (ESI) *m/z* calcd for $C_{35}H_{37}F_9NO_4S$ $[M + H]^+$: 738.2294, found 738.2305.

(*S*)-((1*R*,2*R*,3*R*)-2-(((*S*)-*Tert*-butylsulfinyl)amino)-3-((*S*)-hydroxy(4-(methoxycarbonyl)phenyl)methyl)cycloheptyl)(4-(methoxycarbonyl)phenyl)methyl methyl terephthalate, 2m

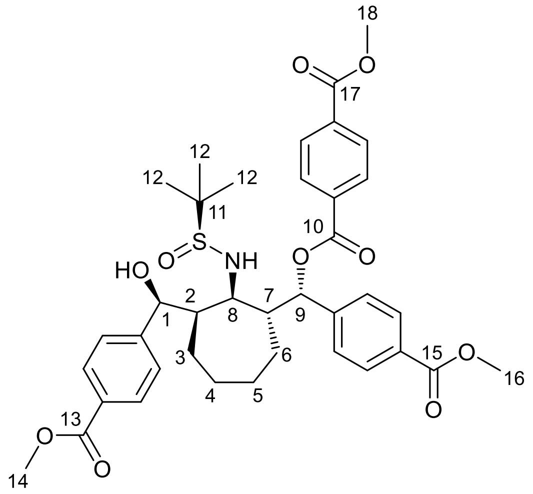

Compound **2m** was prepared using sulfinimine **1a** (0.215 g, 1.0 mmol) and methyl 4-formylbenzoate (0.542 g, 3.3 mmol). The crude compound (98:2 *dr*) was purified using column chromatography on silica gel (1:1, hexane:EtOAc) to give the title compound **2m** as a sticky colourless oil (0.359 g, 51% mixture of diastereomers).

Major diastereomer: $[\alpha]_D^{20}$ + 129.40 (c 0.28, $CHCl_3$). IR ν_{max} (NaCl): 3366 (O-H stretch), 2951 (C-H stretch), 1723 (C=O stretch), 1280 (C-O stretch), 1107 (C-N stretch), 1018 (S=O stretch) cm^{-1}. ^1H NMR (300 MHz, $CDCl_3$) δ 8.14-8.27 (4H, m, Ar-H), 8.01-8.08 (2H, m, Ar-H), 7.91-7.98 (2H, m, Ar-H), 7.35-7.42 (2H, m, Ar-H), 7.29-7.34 (2H, m, Ar-H), 6.33 (1H, d, *J* = 3.2 Hz, H-9), 5.03 (1H, d, *J* = 5.6 Hz, H-1), 4.68 (1H, d, *J* = 6.1 Hz, N-H), 4.57 (1H, d, *J* = 5.8 Hz, O-H), 3.97 (3 × 3H, s, H-14, H-16 and H-18), 3.91, 3.90, 3.77-3.95 (1H, m, H-8), 2.15-2.27 (1H, m, H-7), 1.97-2.08 (1H, m, H-2), 1.40-1.89 (6H, m, H-3 and H-6 and one of each H-4 and H-5), 1.36 (9H, s, H-12), 0.80-1.20 (2H, m, one of each H-4 and H-5). ^{13}C NMR (75.5 MHz, $CDCl_3$) δ 167.1 (C-13, C-15 and C-17), 166.7, 166.3, 164.8 (C-10), 149.5 (Ar-C), 144.6 (Ar-C), 134.6 (Ar-C), 133.6 (Ar-C), 130.1 (2 × Ar-CH), 130.0 (2 × Ar-CH), 129.9 (2 × Ar-CH and Ar-C), 129.5 (2 × Ar-CH), 128.7 (Ar-C), 125.8 (4 × Ar-CH), 76.7 (C-9), 75.7 (C-1), 62.4 (C-8), 56.7 (C-11), 53.6 (C-7), 52.7 (C-14, C-16 and C-18), 52.3, 52.1, 47.8 (C-2), 29.8 (C-4 and C-5), 28.3, 24.7 (C-6), 23.1 (C-12), 21.1 (C-2). HRMS (ESI) *m/z* calcd for $C_{38}H_{45}NO_{10}S$ $[M + Na]^+$: 730.2656, found 730.2656.

(*R*)-((1*R*,2*R*,3*R*)-2-(((*S*)-*Tert*-butylsulfinyl)amino)-3-((*S*)-hydroxy(3-(methoxycarbonyl)phenyl)methyl)cycloheptyl)(3-(methoxycarbonyl)phenyl)methyl methyl isophthalate, 2n

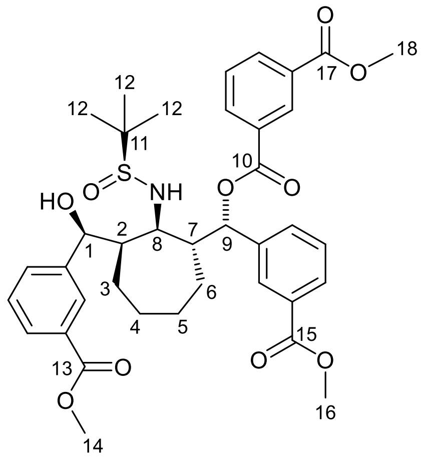

Compound **2n** was prepared using sulfinimine **1a** (0.215 g, 1.0 mmol) and methyl 3-formylbenzoate (0.542 g, 3.3 mmol). The crude compound (97:3 *dr*) was purified using column chromatography on silica gel (1:1, hexane:EtOAc) to give the title compound **2n** as a sticky colourless oil (0.377 g, 53% mixture of diastereomers).

Major diastereomer: $[\alpha]_D^{20}$ + 97.20 (c 0.25, $CHCl_3$). IR ν_{max} (NaCl): 3308 (O-H stretch), 2951 (C-H stretch), 1725 (C=O stretch), 1287 (C-O stretch), 1094 (C-N stretch), 1039 (S=O stretch) cm^{-1}. ^{1}H NMR (300 MHz, $CDCl_3$) δ 8.77-8.80 (1H, m, Ar-H), 8.35 (1H, dt, *J* = 7.8, 1.6 Hz, Ar-H), 8.30 (1H, dt, *J* = 7.8, 1.6 Hz, Ar-H), 7.93-8.06 (3H, m, Ar-H), 7.89 (1H, dt, *J* = 7.7, 1.4 Hz, Ar-H), 7.59-7.66 (1H, m, Ar-H), 7.52-7.57 (1H, m, Ar-H), 7.43-7.51 (2H, m, Ar-H), 7.33-7.40 (1H, m, Ar-H), 6.35 (1H, d, *J* = 3.8 Hz, H-9), 5.03 (1H, d, *J* = 5.9 Hz, H-1), 4.62 (1H, d, *J* = 6.3 Hz, N-H), 4.51 (1H, d, *J* = 5.8 Hz, O-H), 3.98 (3 × 3H, s (overlapping 2 × 3H, s and 1 × 3H, s) H-14, H-16 and H-18), 3.74-3.83 (1H, m, H-8), 3.91, 2.16-2.28 (1H, m, H-7), 1.78-1.91 (1H, m, one of H-6), 2.02-2.13 (1H, m, H-2), 1.41-1.77 (5H, m, H-3 and one of each H-4, H-5 and H-6), 1.38 (9H, s, H-12), 0.90-1.22 (2H, m, one of each H-4 and H-5). ^{13}C NMR (75.5 MHz, $CDCl_3$) δ 167.4 (C-13, C-15 and C-17) 166.8, 166.3, 165.0 (C-10), 144.7 (Ar-C), 140.1 (Ar-C), 134.4 (Ar-CH), 134.1 (Ar-CH), 131.1 (Ar-CH), 131.0 (Ar-C), 130.8 (Ar-C), 130.6 (Ar-CH), 130.4 (Ar-C), 130.4 (Ar-CH), 130.1 (Ar-CH), 129.2 (Ar-CH), 129.1 (Ar-CH), 129.0 (Ar-CH), 128.3 (Ar-CH), 128.1 (Ar-CH), 127.2 (Ar-CH), 126.9 (Ar-CH), 76.4 (C-9), 75.5 (C-1), 62.5 (C-8), 56.7 (C-11), 53.6 (C-7), 52.6 (C-14, C-16 and C-18), 52.4, 52.2, 47.7 (C-2), 29.6 (C-4 and C-5), 28.2, 24.4 (C-6), 23.1 (C-12), 20.9 (C-3). HRMS (ESI) *m/z* calcd for $C_{38}H_{46}NO_{10}S$ $[M + H]^+$: 708.2837, found 708.2841.

(*R*)-((1*R*,2*R*,3*R*)-2-(((*S*)-*Tert*-butylsulfinyl)amino)-3-((*S*)-(4-cyanophenyl)(hydroxy)methyl)cycloheptyl)(4-cyanophenyl)methyl 4-cyanobenzoate, 2o

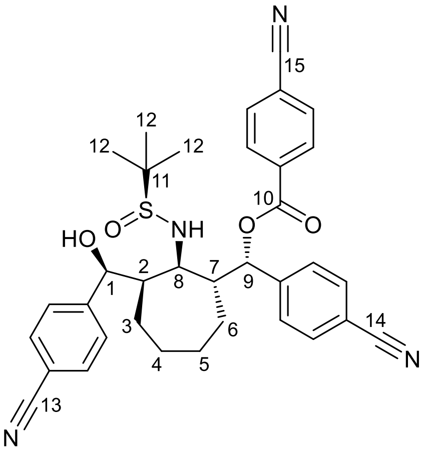

Compound **2o** was prepared using sulfinimine **1a** (0.215 g, 1.0 mmol) and *p*-cyanobenzaldehyde (0.433 g, 3.3 mmol). The crude compound (98:2 *dr*) was purified using column chromatography on silica gel (2:1, hexane:EtOAc) to give the title compound **2o** as a white solid (0.217 g, 36% mixture of diastereomers).

Major diastereomer: Mp 142-146 °C. $[\alpha]_D^{20}$ + 133.60 (c 0.25, $CHCl_3$). IR ν_{max} (NaCl): 3308 (O-H stretch), 2928 (C-H stretch), 2229 (C≡N stretch), 1729 (C=O stretch), 1269 (C-O stretch), 1102 (C-N stretch), 1026 (S=O stretch) cm^{-1}. ^1H NMR (300 MHz, $CDCl_3$) δ 8.20-8.28 (2H, m, Ar-H), 7.80-7.89 (2H, m, Ar-H), 7.63-7.70 (2H, m, Ar-H), 7.56-7.62 (2H, m, Ar-H), 7.35-7.45 (4H, m, Ar-H), 6.33 (1H, d, *J* = 2.8 Hz, H-9), 5.04 (1H, d, *J* = 6.1 Hz, H-1), 4.67 (1H, d, *J* = 6.5 Hz, O-H/N-H), 4.66 (1H, d, *J* = 6.1 Hz, O-H/N-H), 3.67-3.80 (1H, m, H-8), 2.07-2.17 (1H, m, H-7), 1.97-2.05 (1H, m, H-2), 1.45-1.82 (6H, m, H-3 and H-6 and one of each H-4 and H-5), 1.37 (9H, s, H-12), 0.77-1.17 (2H, m, one of each H-4 and H-5). ^{13}C NMR (75.5 MHz, $CDCl_3$) δ 149.6 (Ar-C), 164.0 (C-10), 149.6 (Ar-C), 144.6 (Ar-C), 133.2 (Ar-C), 132.7 (4 × Ar-CH), 132.1 (2 × Ar-CH), 130.4 (2 × Ar-CH), 126.6 (2 × Ar-CH), 126.4 (2 × Ar-CH), 119.0 (C-13, C-14 and C-15), 118.4, 117.8, 117.4 (Ar-C), 112.2 (Ar-C), 110.7 (Ar-C), 76.4 (C-9), 75.0 (C-1), 62.4 (C-8), 57.0 (C-11), 54.0 (C-7), 47.9 (C-2), 29.8 (C-4 and C-5), 28.9, 24.3 (C-6), 23.1 (C-12), 21.1 (C-3). HRMS (ESI) *m*/*z* calcd for $C_{35}H_{37}N_4O_4S$ $[M + H]^+$: 609.2536, found 609.2531.

(*R*)-((1*R*,2*R*,3*R*)-2-(((*S*)-*Tert*-butylsulfinyl)amino)-3-((*S*)-hydroxy(pyridin-4-yl)methyl)cycloheptyl)(pyridin-4-yl)methyl isonicotinate, 2p

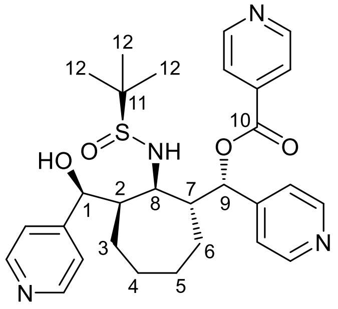

Compound **2p** was prepared using sulfinimine **1a** (0.100 g, 0.46 mmol) and 4-pyridine carboxaldehyde (0.14 mL, 1.52 mmol). The crude compound (90:6:4 *dr*) was purified using column chromatography on silica gel (CH_2Cl_2, 5% MeOH) to give the title compound **2p** as a sticky colourless oil (0.110 g, 45% mixture of diastereomers).

Major diastereomer: $[\alpha]_D^{20}$ + 70.53 (c 0.57, $CHCl_3$). IR ν_{max} (NaCl): 3234 (O-H stretch), 2930 (C-H stretch), 1733 (C=O stretch), 1276 (C-O stretch), 1117 (C-N stretch), 1039 (S=O stretch) cm^{-1}. ^1H NMR (300 MHz, $CDCl_3$) δ 8.76-8.05 (2H, m, Ar-H), 8.33-8.72 (4H, m, Ar-H), 7.93-8.01 (2H, m, Ar-H), 7.11-7.25 (4H, m, Ar-H), 6.31 (1H, d, *J* = 2.2 Hz, H-9), 5.18 (1H, *br* s, O-H), 5.00 (1H, s, H-1), 4.84 (1H, d, *J* = 6.1 Hz, N-H), 3.73-3.84 (1H, m, H-8), 2.10-2.21 (1H, m, H-7), 1.94-2.06 (1H, m, H-2), 1.41-1.84 (6H, m, H-3 and H-6 and one of each H-4 and H-5), 1.37 (9H, s, H-12), 0.76-1.18 (2H, m, one of each H-4 and H-5). ^{13}C NMR (75.5 MHz, $CDCl_3$) δ 164.2 (C-10), 153.4 (Ar-C), 151.1 (2 × Ar-CH), 150.3 (2 × Ar-CH), 149.6 (2 × Ar-CH), 148.3 (Ar-C), 136.7 (Ar-C), 123.0 (2 × Ar-CH), 121.1 (2 × Ar-CH), 120.6 (2 × Ar-CH), 76.0 (C-9), 74.4 (C-1), 62.3 (C-11), 56.9 (C-8), 53.5 (C-7), 47.6 (C-2), 30.0 (C-4 and C-5), 28.9, 24.5 (C-6), 23.1 (C-12), 21.2 (C-3). HRMS (ESI) *m*/*z* calcd for $C_{29}H_{36}N_4O_4SNa$ $[M + Na]^+$: 559.2349, found 559.2344.

(*S*)-*N*-((2*R*,7*R*)-2,7-Bis((*S*)-furan-2-yl(hydroxy)methyl)cycloheptyl)-2-methylpropane-2-sulfinamide, 2s

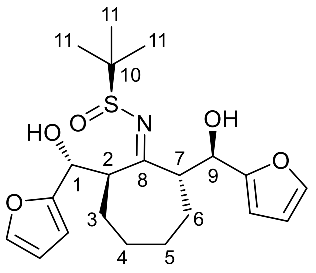

Compound **2s** was prepared using sulfinimine **1a** (0.215 g, 1 mmol) and furfural (0.27 mL, 3.3 mmol). The crude compound (80:20 *dr*) was purified using column chromatography on silica gel (2:1, hexane:EtOAc) to give the title compound **2s** as a sticky brown oil (0.242 g, 59% mixture of diastereomers).

Major diastereomer: $[\alpha]_D^{20}$ + 82.20 (c 0.25, $CHCl_3$). IR v_{max} (NaCl): 3351 (O-H stretch), 2928 (C-H stretch), 1614 (C=N stretch), 1151 (C-O stretch), 1051 (S=O stretch) cm^{-1}. ^1H NMR (300 MHz, $CDCl_3$) δ 7.32-7.47 (2H, m, Ar-H), 6.24-6.43 (4H, m, Ar-H), 5.15 (1H, d, *J* = 10.8 Hz, O-H), 5.08 (1H, dd, *J* = 9.1, 2.1 Hz, H-1), 4.57 (1H, t, *J* = 10.5 Hz, H-9), 4.14 (1H, dt, *J* = 10.4, 6.6 Hz, H-7), 3.53 (1H, d, *J* = 2.3 Hz, O-H), 3.24-3.36 (1H, m, H-2), 1.52-1.89 (4H, m, one of each H-3, H-4, H-5 and H-6), 1.35 (9H, s, H-11), 0.94-1.40 (4H, m, one of each H-3, H-4, H-5 and H-6). ^{13}C NMR (75.5 MHz, $CDCl_3$) δ 192.7 (C-8), 154.4 (2 × Ar-C), 142.7 (Ar-CH), 142.5 (Ar-CH), 110.3 (2 × Ar-CH), 108.5 (Ar-CH), 107.8 (Ar-CH), 70.0 (C-1), 67.7 (C-9), 58.1 (C-10), 53.6 (C-7), 51.9 (C-2), 32.2 (C-3, C-4, C-5 and C-6), 29.7, 28.2, 25.8, 22.6 (C-11). HRMS (ESI) *m*/*z* calcd for $C_{21}H_{29}NO_5SNa$ $[M + Na]^+$: 430.1659, found 430.1658.

Absolute stereochemistry was assigned by crystallographic data.

Note: Assignment of H-1 and H-9 was based on an intramolecular hydrogen-bond between the hydroxyl proton (C_1H-OH) and the sulfoxide moiety which would presumably lead to a more downfield shift for H-1. This hydrogen-bonding interaction was evident in the crystal structure.

IV. Synthesis and characterisation of cyclopentanone double aldol-Tishchenko products 3a-c

Procedure for the synthesis of cyclopentanone 3-amino-1,5-diol derivatives

To a Schlenk tube under N_2 atmosphere, containing diisopropylamine (1.2 equiv) in anhydrous THF (5 mL), was added *n*-BuLi (1.1 equiv) at 0 °C. The mixture was allowed to stir at 0 °C for 20 min to generate a solution of LDA. ***Tert*-butanesulfinimine** (1.0 equiv) was carefully weighed out and the amount added was accurately recorded. The **sulfinimine** was then added slowly (neat), dropwise at 0 °C. After the reaction mixture was allowed to stir for 1 h at 0 °C, the solution was cooled to -78 °C and freshly distilled **aldehyde** (3.3 equiv) was added slowly (neat), dropwise. The reaction mixture was kept at -78 °C for 3 h and was allowed warm to -20 °C over 16 h.

Work-up conditions as per 1 mmol of sulfinimine

The reaction was quenched with sat. aq. NH_4Cl solution (1.5 mL). Sat. aq. NH_4Cl (10 mL) was added and the mixture was extracted with EtOAc (3 × 20 mL). The organic layers were combined, dried over anhydrous $MgSO_4$, filtered and concentrated under reduced pressure to afford the crude product which was purified using column chromatography on silica gel.

(*R*)-((1*R*,2*R*,3*R*)-2-(((*S*)-*Tert*-butylsulfinyl)amino)-3-((*S*)-hydroxy(phenyl)methyl)cyclopentyl)(phenyl)methyl benzoate, 3a

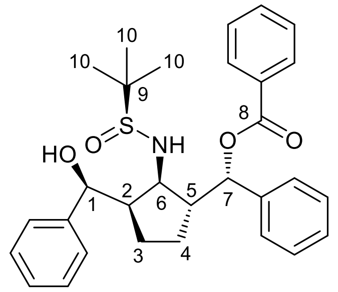

Compound **3a** was prepared using sulfinimine **1b** (0.187 g, 1 mmol) and benzaldehyde (0.34 mL, 3.3 mmol). The crude compound (90:6:4 *dr*) was purified using column chromatography on silica gel (2:1, hexane:EtOAc) to give the title compound **3a** as a sticky pale yellow oil (0.245 g, 48% mixture of diastereomers).

Major diastereomer: $[\alpha]_D^{20}$ + 52.00 (c 0.05, $CHCl_3$). IR ν_{max} (NaCl): 3583 (N-H stretch), 1722 (C=O stretch), 1110 (C-N stretch), 1026 (S=O stretch) cm^{-1}. ^1H NMR (300 MHz, $CDCl_3$) δ 8.06-8.15 (2H, m, Ar-H), 7.57-7.66 (1H, m, Ar-H), 7.44-7.55 (2H, m, Ar-H), 7.18-7.37 (10H, m, Ar-H), 6.26 (1H, d, *J* = 3.2 Hz, H-7), 4.55-4.67 (2H, m, O-H and H-1), 4.14 (1H, d, *J* = 6.5 Hz, N-H), 3.51 (1H, dt, *J* = 9.5, 6.6 Hz, H-6), 2.44 (1H, dq, *J* = 9.2, 3.2 Hz, H-5), 2.30 (1H, quint, *J* = 9.1 Hz, H-2), 1.69-1.90 (1H, m, one of H-4), 1.40-1.60 (1H, m, one of H-4), 1.29 (9H, s, H-10), 1.20-1.36 (2H, m, H-3). ^{13}C NMR (75.5 MHz, $CDCl_3$) δ 165.8 (C-8), 143.3 (Ar-C), 140.2 (Ar-C), 133.4 (Ar-CH), 130.3 (Ar-C), 129.8 (2 × Ar-

CH), 128.7 (2 × Ar-CH), 128.6 (2 × Ar-CH), 128.4 (2 × Ar-CH), 127.7 (2 × Ar-CH), 126.7 (2 × Ar-CH), 125.9 (2 × Ar-CH), 78.6 (C-1), 74.1 (C-7), 64.2 (C-6), 56.2 (C-9), 53.9 (C-5), 52.5 (C-2), 25.8 (C-3), 22.9 (C-10), 21.5 (C-10). HRMS (ESI) *m/z* calcd for $C_{30}H_{36}NO_4S$ [M + H]$^+$: 506.2360, found 506.2364.

(*R*)-((1*R*,2*R*,3*R*)-2-(((*S*)-*Tert*-butylsulfinyl)amino)-3-((*S*)-(4-ethylphenyl)(hydroxy)methyl)cyclopentyl)(4-ethylphenyl)methyl 4-ethylbenzoate, 3b

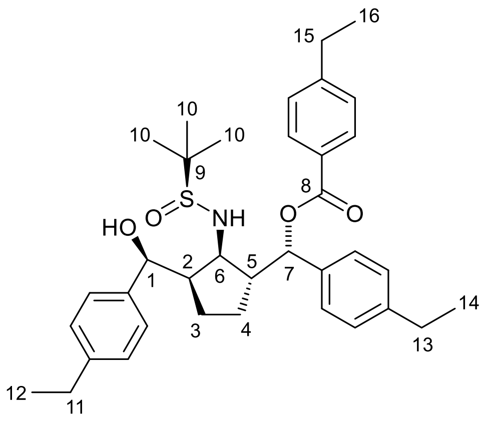

Compound **3b** was prepared using sulfinimine **1b** (0.122 g, 0.65 mmol) and *p*-ethylbenzaldehyde (0.29 mL, 2.15 mmol). The crude compound (94:6 *dr*) was purified using column chromatography on silica gel (1:1, hexane:EtOAc) to give the title compound **3b** as a sticky yellow oil (0.173 g, 45% mixture of diastereomers).

Major diastereomer: $[\alpha]_D^{20}$ + 73.00 (c 0.1, $CHCl_3$). IR v_{max} (NaCl): 3583 (N-H stretch), 2964 (C-H stretch), 1722 (C=O stretch), 1177 (C-O stretch), 1103 (C-N stretch), 1049 (S=O stretch) cm^{-1}. ^{1}H NMR (300 MHz, $CDCl_3$) δ 8.00-8.06 (2H, m Ar-H), 7.17-7.35 (6H, m, Ar-H), 7.07-7.17 (4H, m, Ar-H), 6.20 (1H, d, *J* = 3.3 Hz, H-7), 4.58 (overlapping 1H, *br* s, O-H and 1H, d, *J* = 9.0 Hz, H-1), 4.17 (1H, d, *J* = 6.5 Hz, N-H), 3.50 (1H, dt, *J* = 9.4, 6.6 Hz, H-6), 2.73 (3 × 2H, q, *J* = 7.6 Hz (overlapping 2 × 2H, q and 1 × 2H, q) H-11, H-13 and H-15), 2.60, 2.44 (1H, dq, *J* = 9.1, 3.5 Hz, H-5), 2.30 (1H, quint, *J* = 9.0 Hz, H-2), 1.69-1.88 (1H, m, one of H-4), 1.45-1.62 (1H, m, one of H-4), 1.28 (9H, s, H-10), 1.28 (3 × 3H, t, *J* = 7.6 Hz, H-12, H-14 and H-16), 1.20, 1.19, 1.15-1.33 (2H, m, H-3). ^{13}C NMR (75.5 MHz, $CDCl_3$) δ 165.9 (C-8), 150.2 (Ar-C), 143.6 (2 × Ar-C), 140.6 (Ar-C), 137.5 (Ar-C), 130.0 (2 × Ar-CH), 128.2 (2 × Ar-CH), 128.0 (2 × Ar-CH), 127.9 (overlapping 2 × Ar-CH and Ar-C), 126.7 (2 × Ar-CH), 126.0 (2 × Ar-CH), 78.5 (C-1), 74.1 (C-7), 64.3 (C-6), 56.1 (C-9), 53.9 (C-5), 52.5 (C-2), 29.1 (overlapping C-11, C-13 and C-15), 28.6, 26.0 (C-3), 22.9 (C-10), 21.7 (C-4), 15.6 (overlapping C-12, C-14 and C-16), 15.4. HRMS (ESI) *m/z* calcd for $C_{36}H_{47}NO_4SNa$ [M + Na]$^+$: 612.3118, found 612.3120.

(*R*)-((1*R*,2*R*,3*R*)-2-(((*S*)-*Tert*-butylsulfinyl)amino)-3-((*S*)-hydroxy(4-isopropylphenyl)methyl)cyclopentyl)(4-isopropylphenyl)methyl 4-isopropylbenzoate, 3c

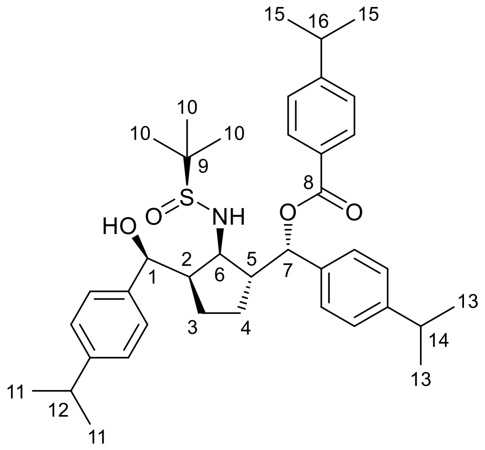

Compound **3c** was prepared using sulfinimine **1b** (0.176 g, 0.94 mmol) and *p*-*iso*-propyl benzaldehyde (0.47 mL, 3.1 mmol). The crude compound (93:7 *dr*) was purified using column chromatography on silica gel (2:1, hexane:EtOAc) to give the title compound **3c** as a sticky yellow oil (0.285 g, 48% mixture of diastereomers).

Major diastereomer: $[\alpha]_D^{20}$ + 38.40 (c 0.25, $CHCl_3$). IR v_{max} (NaCl): 3583 (N-H stretch), 2960 (C-H stretch), 1721 (C=O stretch), 1107 (C-N stretch), 1053 (S=O stretch) cm^{-1}. ^1H NMR (300 MHz, $CDCl_3$) δ 7.98-8.07 (2H, m, Ar-H), 7.09-7.39 (10H, m, Ar-H), 6.20 (1H, d, *J* = 3.2 Hz, H-7), 4.58 (1H, d, *J* = 9.2 Hz, H-1), 4.54 (1H, *br* s, O-H), 4.10 (1H, d, *J* = 5.9 Hz, N-H), 3.36-3.70 (1H, m, H-6), 2.99 (3 × 1H, sept, *J* = 6.9 Hz (overlapping 2 × 1H, sept and 1 × 1H, sept) H-12, H-14 and H-16), 2.86, 2.45 (1H, dq, *J* = 9.0, 3.4 Hz, H-5), 2.31 (1H, quint, *J* = 8.7 Hz, H-2), 1.69-1.89 (1H, m, one of H-4), 1.46-1.64 (1H, m, one of H-4), 1.27 (9H, s, H-10), 1.29 (3 × 6H, d, *J* = 6.9 Hz, H-11, H-13 and H-15), 1.22, 1.20, 1.16-1.32 (2H, m, H-3). ^{13}C NMR (75.5 MHz, $CDCl_3$) δ 165.9 (C-8), 154.7 (Ar-C), 148.2 (2 × Ar-C), 140.7 (Ar-C), 137.6 (Ar-C), 130.0 (2 × Ar-CH), 128.0 (Ar-C), 126.8 (2 × Ar-CH), 126.7 (2 × Ar-CH), 126.6 (2 × Ar-CH), 126.4 (2 × Ar-CH), 126.1 (2 × Ar-CH), 78.5 (C-1), 74.2 (C-7), 64.3 (C-6), 56.1 (C-9), 53.8 (C-5), 52.5 (C-2), 34.4 (overlapping C-12, C-14 and C-16), 33.9, 26.1 (C-3), 24.1 (overlapping C-11, C-13 and C-15), 23.9, 22.9 (C-10), 21.8 (C-4). HRMS (ESI) *m*/*z* calcd for $C_{39}H_{53}NO_4SNa$ $[M + Na]^+$: 654.3588, found 654.3584.

V. Synthesis and characterisation of 2-butanone double aldol-Tishchenko products 4a-d

Procedure for the synthesis of 2-butanone 3-amino-1,5-diol derivatives

To a Schlenk tube under N_2 atmosphere, containing diisopropylamine (1.2 equiv) in anhydrous THF (5 mL per mmol of sulfinyl imine), was added *n*-BuLi (1.1 equiv) at 0 °C. The mixture was allowed to stir at 0 °C for 20 min to generate a solution of LDA. ***Tert*-butanesulfinimine** (1.0 equiv) was carefully weighed out and the amount added was accurately recorded. The **sulfinimine** was then added slowly (neat), dropwise at -78 °C. After the reaction mixture was allowed to stir for 1 h at -78 °C, freshly distilled **aldehyde** (3.3 equiv) was added slowly, (neat) dropwise. The reaction mixture was kept at -78 °C for 3 h and was allowed warm to -20 °C over 16 h.

Work-up conditions as per 1 mmol of sulfinimine

The reaction was quenched with sat. aq. NH_4Cl solution (1.5 mL). Sat. aq. NH_4Cl (10 mL) was added and the mixture was extracted with EtOAc (3 × 20 mL). The organic layers were combined, dried over anhydrous $MgSO_4$, filtered and concentrated under reduced pressure to afford the crude product which was purified using column chromatography on silica gel.

Note: Absolute stereochemistry was not confirmed for the 2-butanone series.

3-(((*S*)-*Tert*-butylsulfinyl)amino)-5-hydroxy-4-methyl-1,5-diphenylpentyl benzoate, 4a

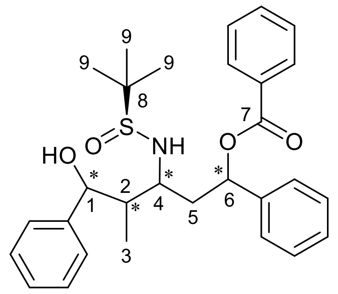

Compound **4a** was prepared using sulfinimine **1c** (0.084 g, 0.48 mmol) and benzaldehyde (0.16 mL, 1.58 mmol). The crude compound (>98:2 *dr*) was purified using column chromatography on silica gel (1:1, hexane:EtOAc) to give the title compound **4a** as a sticky yellow oil (0.076 g, 32% mixture of diastereomers).

Major diastereomer: $[\alpha]_D^{20}$ + 60.40 (c 0.25, $CHCl_3$). IR v_{max} (NaCl): 3307 (O-H stretch), 2927 (C-H stretch), 1720 (C=O stretch), 1270 (C-O stretch), 1108 (C-N stretch), 1027 (S=O stretch) cm^{-1}. ^{1}H NMR (300 MHz, $CDCl_3$) δ 8.00-8.08 (2H, m, Ar-H), 7.52-7.63 (1H, m, Ar-H), 7.16-7.51 (12H, m, Ar-H), 6.09 (1H, dd, *J* = 10.3, 3.6 Hz, H-6), 5.05 (1H, dd, *J* = 4.9, 2.2 Hz, H-1), 4.30 (1H, d, *J* = 6.8 Hz, N-H), 4.28 (1H, d, *J* = 5.1 Hz, O-H), 3.75-3.86 (1H, m, H-4), 2.20 (1H, ddd, *J* = 14.4, 10.2, 4.1 Hz, one of H-

5), 1.99-2.12 (2H, m, H-2 and one of H-5), 1.31 (9H, s, H-9), 0.89 (3H, d, *J* = 7.1 Hz, H-3). ^{13}C NMR (75.5 MHz, $CDCl_3$) δ 165.8 (C-7), 144.1 (Ar-C), 140.8 (Ar-C), 133.2 (Ar-CH), 130.3 (Ar-CH), 129.8 (2 × Ar-CH), 128.8 (2 × Ar-CH), 128.6 (2 × Ar-CH), 128.3 (2 × Ar-CH), 128.2 (2 × Ar-CH), 126.9 (Ar-CH), 126.3 (2 × Ar-CH), 125.9 (2 × Ar-CH), 75.9 (C-1), 73.5 (C-6), 58.1 (C-4), 56.6 (C-8), 44.4 (C-2), 43,4 (C-5), 23.0 (C-9), 6.1 (C-3). HRMS (ESI) *m*/*z* calcd for $C_{29}H_{35}NO_4SNa$ $[M + Na]^+$: 516.2179, found 516.2175.

Note: Utilising 0.8 equivalents of LDA and warming the reaction mixture to -20 °C over a period of 16 h afforded **3a** in an improved yield of 45%.

3-(((*S*)-*Tert*-butylsulfinyl)amino)-5-hydroxy-4-methyl-1,5-di-*p*-tolylpentyl 4-methylbenzoate, 4b

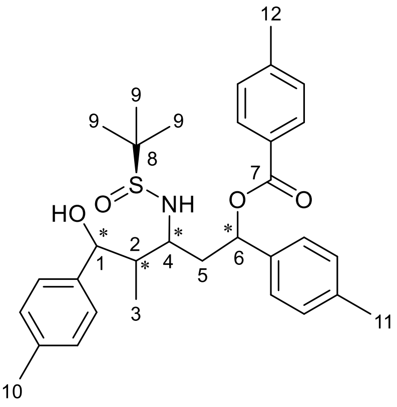

Compound **4b** was prepared using sulfinimine **1c** (0.166 g, 0.95 mmol) and *p*-tolualdehyde (0.51 mL, 4.3 mmol). The crude compound (~97:3 *dr*) was purified using column chromatography on silica gel (2:1, hexane:EtOAc) to give the title compound **4b** as a sticky colourless oil (0.224 g, 44% mixture of diastereomers).

Major diastereomer: $[\alpha]_D^{20}$ + 78.00 (c 0.1, $CHCl_3$). IR ν_{max} (NaCl): 3339 (O-H stretch), 2922 (C-H stretch), 1718 (C=O stretch), 1271 (C-O stretch), 1102 (C-N stretch), 1041 (S=O stretch) cm^{-1}. ^{1}H NMR (300 MHz, $CDCl_3$) δ 7.86-8.00 (2H, m, Ar-H), 7.02-7.37 (10H, m, Ar-H), 6.02 (1H, dd, *J* = 10.0, 3.5 Hz, H-6), 5.00 (1H, *br* s, H-1), 4.29 (1H, d, *J* = 4.6 Hz, N-H), 4.18 (1H, *br* s, O-H), 3.67-3.85 (1H, *br* m, H-4), 2.41 (3 × 3H, s (overlapping 2 × 3H, s and 1 × 3H, s) H-10, H-11 and H-12), 2.32, 2.19 (1H, ddd, *J* = 14.3, 9.9, 3.3 Hz, one of H-5), 1.95-2.09 (2H, m, H-2 and one of H-5), 1.29 (9H, s, H-9), 0.88 (3H, d, *J* = 6.9 Hz, H-3). ^{13}C NMR (75.5 MHz, $CDCl_3$) δ 165.9 (C-7), 143.8 (Ar-C), 141.2 (Ar-C), 138.0 (Ar-C), 137.9 (Ar-C), 136.4 (Ar-C), 129.8 (2 × Ar-CH), 129.5 (2 × Ar-CH), 129.2 (2 × Ar-CH), 128.9 (2 × Ar-CH), 127.7 (Ar-C), 126.3 (2 × Ar-CH), 125.8 (2 × Ar-CH), 75.9 (C-1), 73.3 (C-6), 58.0 (C-4), 56.5 (C-8), 44.3 (C-2), 43.2 (C-5), 23.0 (C-9), 21.8 (C-10, C-11 and C-12), 21.3, 21.2, 6.2 (C-3). HRMS (ESI) *m*/*z* calcd for $C_{32}H_{41}NO_4SNa$ $[M + Na]^+$: 558.2630, found 558.2627.

Note: 4.5 equivalents of *p*-tolualdehyde was utilised in an attempt to improve the yield.

3-(((*S*)-*Tert*-butylsulfinyl)amino)-5-hydroxy-4-methyl-1,5-di-*m*-tolylpentyl 3-methylbenzoate, 4c

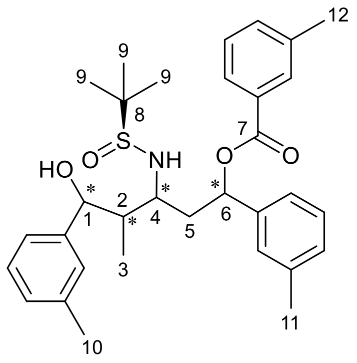

Compound **4c** was prepared using sulfinimine **1c** (0.114 g, 0.65 mmol) and *m*-tolualdehyde (0.25 mL, 2.15 mmol). The crude compound (~90:10 *dr*) was purified using column chromatography on silica gel (2:1, hexane:EtOAc) to give the title compound **4c** as a sticky pale yellow oil (0.131 g, 38% mixture of diastereomers).

Major diastereomer: $[\alpha]_D^{20}$ + 59.20 (c 0.25, $CHCl_3$). IR ν_{max} (NaCl): 3305 (O-H stretch), 2961 (C-H stretch), 1719 (C=O stretch), 1275 (C-O stretch), 1105 (C-N stretch), 1041 (S=O stretch) cm^{-1}. ^1H NMR (300 MHz, $CDCl_3$) δ 7.82-7.92 (2H, m, Ar-H), 6.96-7.45 (10H, m, Ar-H), 6.04 (1H, dd, *J* = 10.2, 3.5 Hz, H-6), 5.03 (1H, *br* s, H-1), 4.28 (overlapping 1H, d, *J* = 6.5 Hz, N-H and 1H, *br* s, O-H), 3.74-3.92 (1H, m, H-4), 2.42 (3 × 3H, s (overlapping 2 × 3H, s and 1 × 3H, s) H-10, H-11 and H-12), 2.33, 2.20 (1H, ddd, *J* = 14.5, 10.3, 4.2 Hz, one of H-5), 1.97-2.11 (2H, m, H-2 and one of H-5), 1.32 (9H, s, H-9), 0.88 (3H, d, *J* = 7.1 Hz, H-3). ^{13}C NMR (75.5 MHz, $CDCl_3$) δ 166.0 (C-7), 144.2 (Ar-C), 140.8 (Ar-C), 138.5 (Ar-C), 138.4 (Ar-C), 137.8 (Ar-C), 134.1 (Ar-CH), 130.3 (Ar-C), 130.3 (Ar-CH), 129.0 (Ar-CH), 128.7 (Ar-CH), 128.5 (Ar-CH), 128.1 (Ar-CH), 127.6 (Ar-CH), 127.1 (Ar-CH), 126.9 (Ar-CH), 126.5 (Ar-CH), 123.4 (Ar-CH), 122.9 (Ar-CH), 76.1 (C-1), 73.5 (C-6), 58.2 (C-4), 56.6 (C-8), 44.3 (C-2), 43.4 (C-5), 23.0 (C-9), 21.6 (overlapping C-10, C-11 and C-12), 21.5, 5.9 (C-3). HRMS (ESI) *m*/*z* calcd for $C_{32}H_{42}NO_4S$ $[M + H]^+$: 536.2829, found 536.2831.

Note: Separation of diastereomers could not be achieved on this occasion.

3-(((*S*)-*Tert*-butylsulfinyl)amino)-1,5-bis(3-fluorophenyl)-5-hydroxy-4-methylpentyl 3-fluorobenzoate, 4d

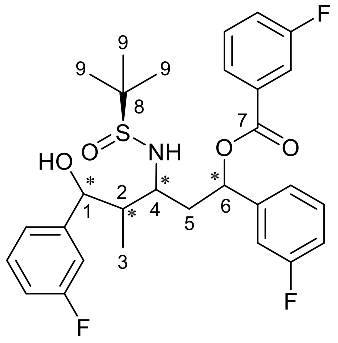

Compound **4d** was prepared using sulfinimine **1c** (0.092 g, 0.52 mmol) and *m*-fluorobenzaldehyde (0.18 mL, 1.72 mmol). The crude compound was purified using column chromatography on silica gel (1:1, hexane:EtOAc) to give the title compound **4d** as a sticky colourless oil (0.135 g, 47% of pure diastereomer).

Major diastereomer: $[\alpha]_D^{20}$ + 68.44 (c 0.225, $CHCl_3$). IR v_{max} (NaCl): 3338 (O-H stretch), 2965 (C-H stretch), 1725 (C=O stretch), 1269 (C-O stretch), 1093 (C-N stretch), 1039 (S=O stretch) cm^{-1}. ^1H NMR (400 MHz, $CDCl_3$) δ 7.86 (1H, dt, *J* = 7.8, 1.1 Hz, Ar-H), 7.73 (1H, ddd, *J* = 9.2, 2.6, 1.5 Hz, Ar-H), 7.46 (1H, dt, *J* = 8.1, 5.5 Hz, Ar-H), 7.20-7.38 (3H, m, Ar-H), 7.07-7.18 (4H, m, Ar-H), 6.99 (1H, dddd, *J* = 9.3, 5.4, 2.6, 0.9 Hz, Ar-H), 6.90 (1H, dddd, *J* = 9.1, 3.7, 2.6, 0.9 Hz, Ar-H), 6.07 (1H, dd, *J* = 10.5, 3.2 Hz, H-6), 5.08 (1H, dd, *J* = 5.2, 1.4 Hz, H-1), 4.54 (1H, d, *J* = 5.3 Hz, N-H), 4.26 (1H, d, *J* = 6.7 Hz, O-H), 3.75-3.89 (1H, m, H-4), 2.21 (1H, ddd, *J* = 14.5, 10.6, 4.5 Hz, one of H-5), 1.96-2.08 (2H, m, H-2 and one of H-5), 1.33 (9H, s, H-9), 0.85 (3H, d, *J* = 7.1 Hz, H-3). ^{13}C NMR (100.6 MHz, $CDCl_3$) δ 164.6 (d, $^4J_{C-F}$ = 2.9 Hz, C-7), 163.1 (d, $^1J_{C-F}$ = 246.4 Hz, Ar-C), 163.0 (d, $^1J_{C-F}$ = 245.1 Hz, Ar-C), 162.7 (d, $^1J_{C-F}$ = 247.0 Hz, Ar-C), 146.9 (d, $^3J_{C-F}$ = 6.7 Hz, Ar-C), 143.0 (d, $^3J_{C-F}$ = 6.9 Hz, Ar-C), 132.1 (d, $^3J_{C-F}$ = 7.4 Hz, Ar-C), 130.6 (d, $^3J_{C-F}$ = 8.2 Hz, Ar-CH), 130.4 (d, $^3J_{C-F}$ = 8.0 Hz, Ar-CH), 129.6 (d, $^3J_{C-F}$ = 8.2 Hz, Ar-CH), 125.5 (d, $^4J_{C-F}$ = 3.0 Hz, Ar-CH), 121.9 (d, $^4J_{C-F}$ = 2.9 Hz, Ar-CH), 121.3 (d, $^4J_{C-F}$ = 2.6 Hz, Ar-CH), 120.6 (d, $^2J_{C-F}$ = 21.2 Hz, Ar-CH), 116.7 (d, $^2J_{C-F}$ = 22.9 Hz, Ar-CH), 115.4 (d, $^2J_{C-F}$ = 21.0 Hz, Ar-CH), 113.7 (d, $^2J_{C-F}$ = 21.1 Hz, Ar-CH), 113.2 (d, $^2J_{C-F}$ = 22.1 Hz, Ar-CH), 113.0 (d, $^2J_{C-F}$ = 22.3 Hz, Ar-CH), 75.3 (C-1), 73.1 (C-6), 58.0 (C-4), 56.8 (C-8), 44.4 (C-2), 43.5 (C-5), 23.0 (C-9), 5.8 (C-3). HRMS (ESI) *m*/*z* calcd for $C_{29}H_{32}F_3NO_4SNa$ $[M + Na]^+$: 570.1896, found 570.1889.

Note: Diastereoselectivity could not be determined from the ^1H NMR spectrum of the crude reaction mixture due to overlapping signals.

VI. Regioselective cleavage of *tert*-butyl sulfinyl group

Procedure for cleavage of *tert*-butyl sulfinyl auxiliary

(*R*)-((1*R*,2*R*,3*R*)-2-Amino-3-((*S*)-(3-fluorophenyl)(hydroxy)methyl)cycloheptyl)(3-fluorophenyl)methyl 3-fluorobenzoate, 2q

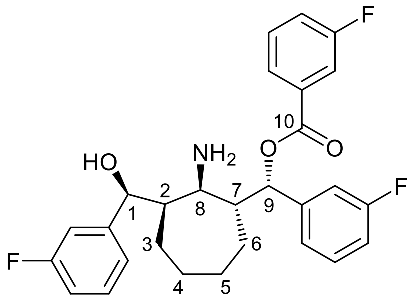

To a solution of 3-amino-1,5-diol derivative **2i** (0.048 g, 0.082 mmol) in EtOH (0.33 mL) was added diisopropyl ether (0.34 mL). The resulting solution was cooled to 0 °C and acetyl chloride (0.019 g, 0.018 mL, 0.25 mmol) was added dropwise. The mixture was stirred at 0 °C overnight. The reaction mixture was concentrated under reduced pressure. The residue was dissolved in 0.3 mL of H_2O and subsequently washed with CH_2Cl_2. $NaHCO_3$ (3 mL) was added to the aqueous layer and extracted twice with CH_2Cl_2 (0.5 mL). The organic layers were combined, dried over anhydrous $MgSO_4$, filtered and concentrated under reduced pressure to afford the crude product which was purified using column chromatography on silica gel (CH_2Cl_2, 5% MeOH) to give the title compound **2q** as a sticky colourless oil (0.032 g, 80% of pure diastereomer).

Major diastereomer: $[\alpha]_D^{20}$ + 113.00 (c 0.1, $CHCl_3$). IR v_{max} (NaCl): 3370 (O-H stretch), 3076 (N-H stretch), 2929 (C-H stretch), 1729 (C=O stretch), 1446 (C-F stretch), 1180 (C-O stretch), 1108 (C-N stretch), 1034 (S=O stretch) cm^{-1}. ^1H NMR (600 MHz, $CDCl_3$) δ 7.89-7.93 (1H, m, Ar-H), 7.75-7.81 (1H, m, Ar-H), 7.50 (1H, dt, *J* = 8.3, 5.5 Hz, Ar-H), 7.31-7.41 (1H, m, Ar-H), 7.21-7.20 (2H, m, Ar-H), 7.14-.7.20 (1H, m, Ar-H), 7.07-7.13 (1H, m, Ar-H), 6.98-7.06 (3H, m, Ar-H), 6.89 (1H, td, *J* = 8.1, 2.3 Hz, Ar-H), 6.23 (1H, d, *J* = 4.2 Hz, H-9), 4.87 (1H, s, H-1), 2.34-3.77 (4H, m, overlapping 2H, *br* s, NH_2, 1H, *br* s, O-H and 1H, m, H-8), 1.90-2.03 (1H, m, H-7), 1.74-1.88 (3H, m, H-2 and one of each H-4/H-5 and H-6), 1.60-1.72 (1H, m, one of H-4/H-5), 1.42-1.59 (2H, m, one of each H-3 and H-6), 1.30-1.40 (1H, m, one of H-3), 1.12-1.23 (1H, m, one of H-4/H-5), 0.91-1.04 (1H, m, one of H-4/H-5). ^{13}C NMR (150.9 MHz, $CDCl_3$) δ 164.8 (d, $^4J_{C-F}$ = 2.8 Hz, C-10), 163.1 (d, $^1J_{C-F}$ = 247.2 Hz, Ar-C), 163.0 (d, $^1J_{C-F}$ = 244.9 Hz, Ar-C), 162.8 (d, $^1J_{C-F}$ = 247.8 Hz, Ar-C), 146.6 (d, $^3J_{C-F}$ = 7.2 Hz, Ar-C), 141.9 (d, $^3J_{C-F}$ = 7.2 Hz, Ar-C), 131.9 (d, $^3J_{C-F}$ = 7.2 Hz, Ar-C), 130.6 (d, $^3J_{C-F}$ = 7.5 Hz, Ar-CH), 130.5 (d, $^3J_{C-F}$ = 8.3 Hz, Ar-CH), 129.5 (d, $^3J_{C-F}$ = 8.3 Hz, Ar-CH), 125.6 (d, $^4J_{C-F}$ = 3.2 Hz, Ar-CH), 122.0 (d, $^4J_{C-F}$ = 3.2 Hz, Ar-CH), 121.3 (d, $^4J_{C-F}$= 2.9 Hz, Ar-CH), 120.8 (d, $^2J_{C-F}$ = 21.0 Hz, Ar-CH), 116.7 (d, $^2J_{C-F}$ = 23.1 Hz, Ar-CH), 115.0 (d, $^2J_{C-F}$ = 21.0 Hz, Ar-CH), 113.4 (d, $^2J_{C-F}$ = 22.1 Hz, 2 × Ar-CH), 113.0

(d, $^{2}J_{C\text{-}F}$ = 22.1 Hz, Ar-CH), 77.7 (C-1), 77.2 (C-9), 57.3 (overlapping C-7 and C-8), 47.4 (C-2), 29.6 (C-4 and C-5), 28.7, 25.7 (C-6), 19.8 (C-3). HRMS (ESI) *m/z* calcd for $C_{28}H_{28}F_3NO_3$ $[M + H]^+$: 484.2094, found 484.2099.

VII. High-resolution mass spectra for lithium double-aldolate crossover experiment

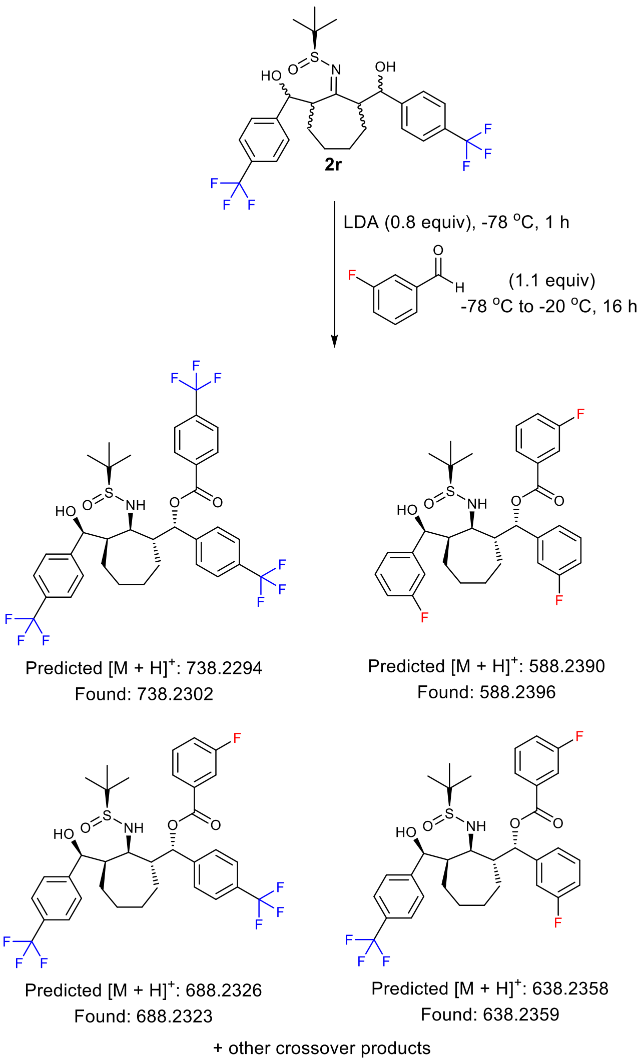

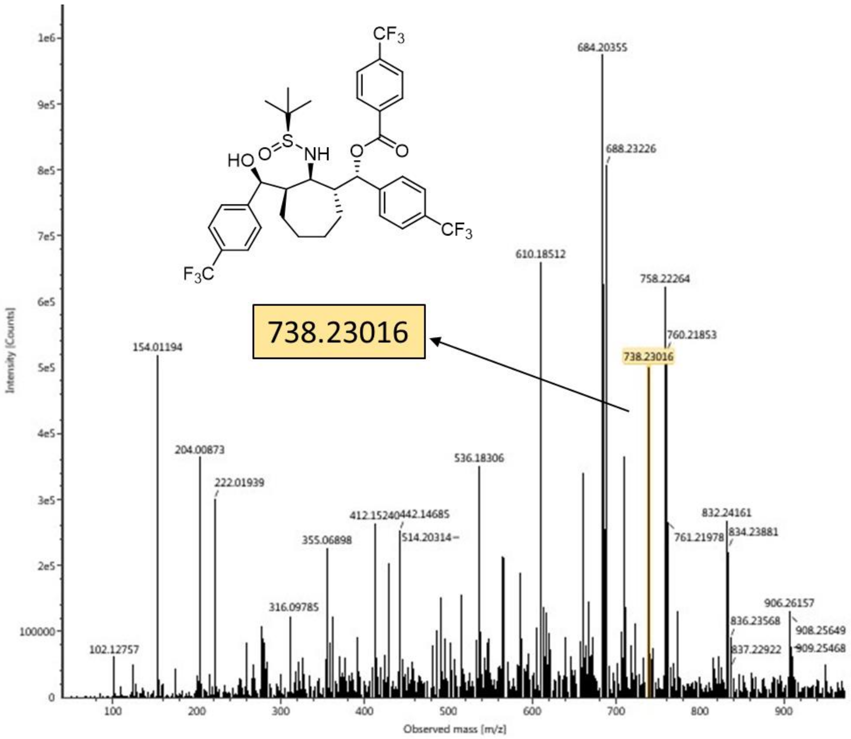

738.23016
HO
NH
O
CF3
F3C
684.20355
688.23226
610.18512
758.22264
760.21853
738.23016
154.01194
204.00873
222.01939
536.18306
412.15240
442.14685
514.20314
355.06898
832.24161
834.23881
761.21978
316.09785
906.26157
836.23568
908.25649
837.22922
909.25468
102.12757
Intensity [Counts]
Observed mass [m/z]

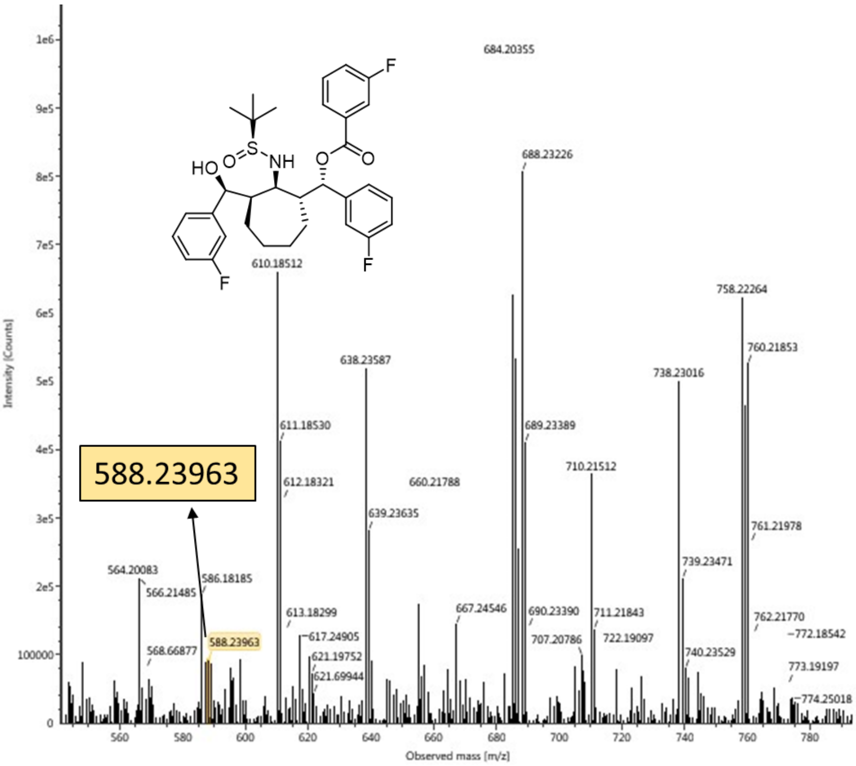

588.23963
HO
NH
O
F
684.20355
688.23226
610.18512
758.22264
760.21853
638.23587
738.23016
611.18530
689.23389
710.21512
612.18321
660.21788
639.23635
761.21978
739.23471
564.20083
566.21485
586.18185
613.18299
667.24546
690.23390
711.21843
762.21770
772.18542
588.23963
617.24905
707.20786
722.19097
568.66877
621.19752
740.23529
773.19197
621.69944
774.25018
Intensity [Counts]
Observed mass [m/z]

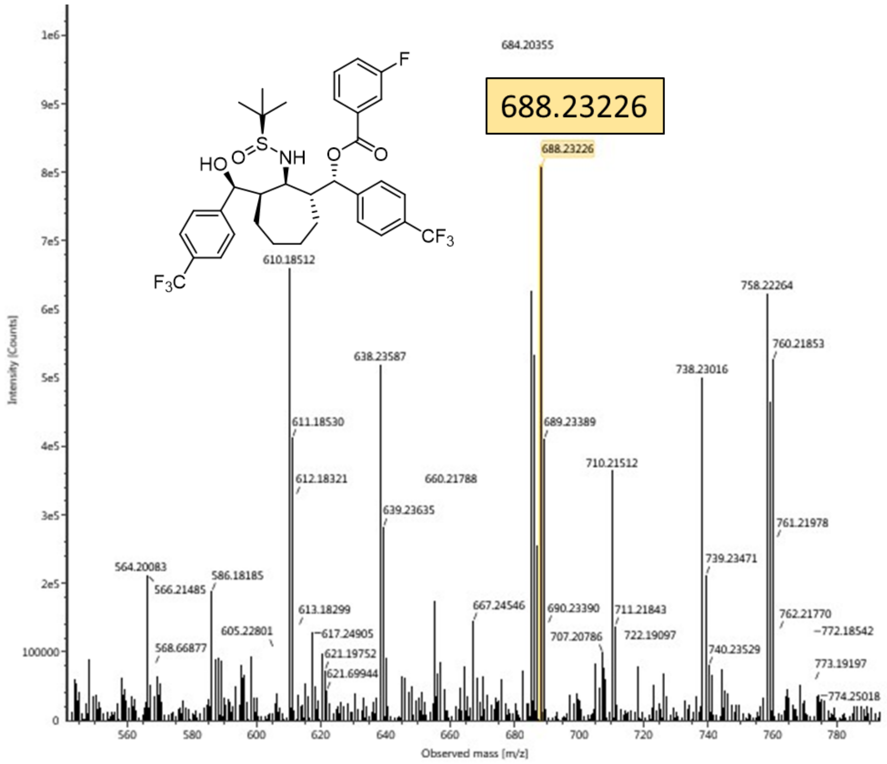
688.23226
Intensity [Counts]
Observed mass [m/z]
684.20355
610.18512
638.23587
758.22264
760.21853
738.23016
689.23389
710.21512
660.21788
639.23635
761.21978
739.23471
564.20083
586.18185
566.21485
667.24546
690.23390
711.21843
762.21770
613.18299
605.22801
617.24905
707.20786
722.19097
772.18542
568.66877
621.19752
740.23529
773.19197
621.69944
774.25018
HO
NH
CF3
F3C
F

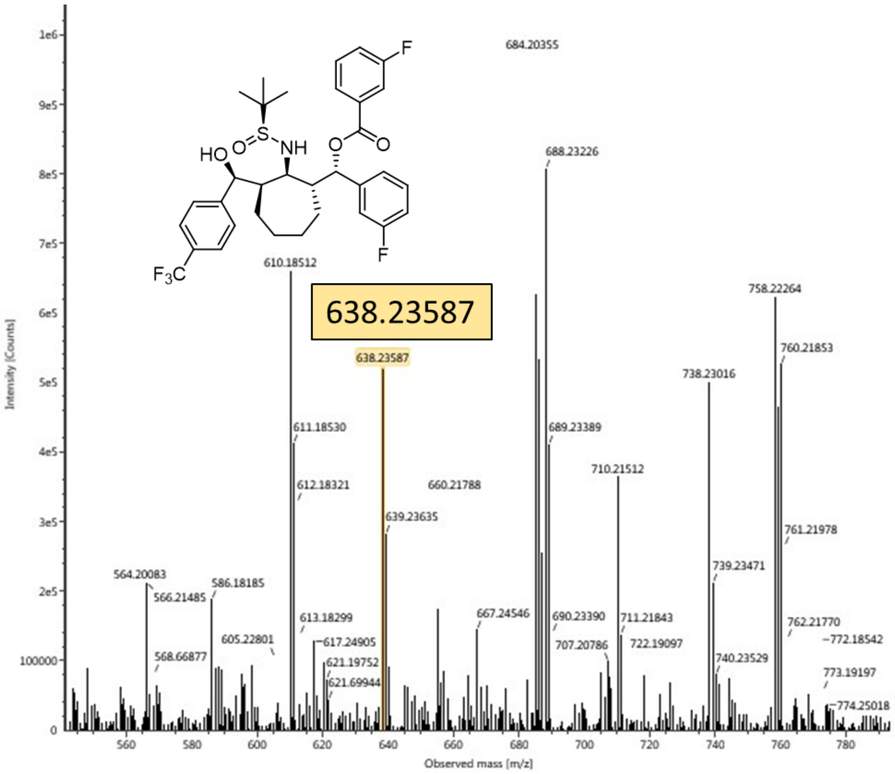
638.23587
Intensity [Counts]
Observed mass [m/z]
684.20355
688.23226
610.18512
758.22264
760.21853
738.23016
611.18530
689.23389
710.21512
612.18321
660.21788
639.23635
761.21978
739.23471
564.20083
586.18185
566.21485
667.24546
690.23390
711.21843
762.21770
613.18299
605.22801
617.24905
707.20786
722.19097
772.18542
568.66877
621.19752
740.23529
773.19197
621.69944
774.25018
HO
NH
F3C
F

VIII. Proposed mechanism

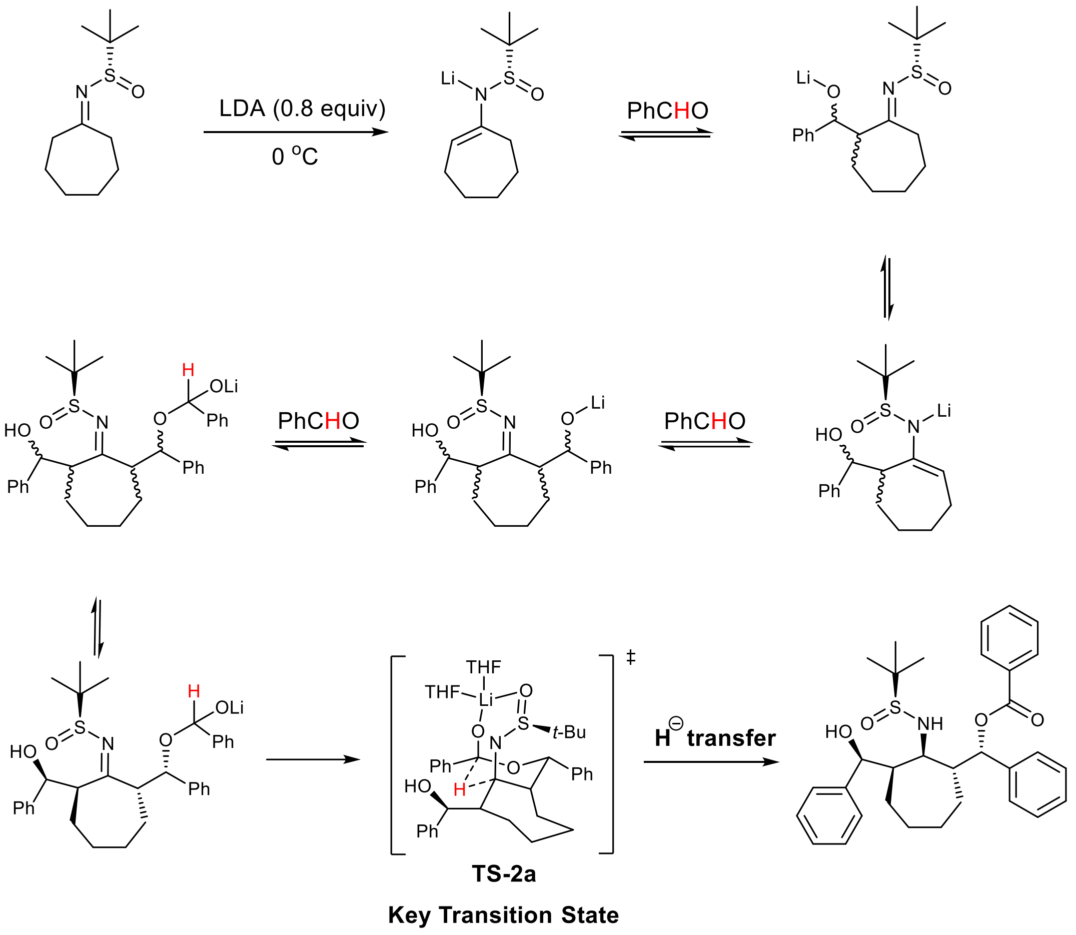

IX. Tentative assignment of stereochemistry for 2-butanone series

It is postulated that the stereochemistry between C-1 and C-3 of double aldol-Tishchenko product **4a** is analogous to the stereochemistry of the acetophenone derived aldol-Tishchenko product (Fig. SI-1). Analysis of the coupling constants for the protons on C-1, C-2 and C-3 and the relationship to the dihedral angle, enables an assumption to be made about the structure of the aldol-Tishchenko product in solution. Given that a similar coupling constant pattern is observed for the double aldol-Tishchenko product **4a** it is possible that it assumes a similar structure where C-1 and C-3 have an *anti*-relationship.

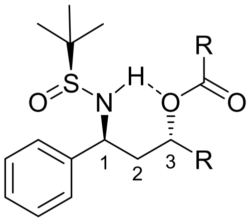

≡

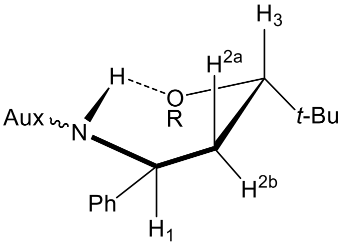

One of H-2: 1H, ddd, J = 14.0, 10.7, 3.0 Hz
One of H-2: 1H, ddd, J = 14.4, 11.0, 1.5 Hz

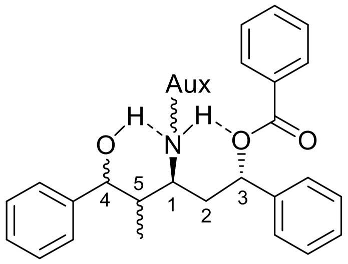

4a
One of H-2: 1H, ddd, J = 14.4, 10.2, 4.1 Hz
One of H-2: 1H, m

Fig. SI-1. Tentative assignment of aldol-Tishchenko stereochemistry for 2-butanone derived 3-amino-1,5-diol derivative

The relative stereochemistry at C-4 and C-5 could be tentatively assigned based on analysis of the vicinal coupling constants using the Karplus equation (H-C-C-H, ^3J). The use of vicinal coupling constants (H-C-C-H, ^3J) for conformational analysis of acyclic systems is much more difficult in comparison to cyclic systems due to the larger number of possible conformations. However, the assignment of relative configuration of aldol products is often made using the 'Stiles-House' ^{1}H NMR method.[5] A prerequisite of this empirical rule is that an intramolecular hydrogen-bond exists between the carbonyl oxygen and the hydroxyl proton resulting in the formation of an intramolecularly hydrogen-bonded six-membered structure. This predictable conformation leads to a corresponding 'somewhat' predictable $^3J_{H\text{-}H}$ coupling constant (Fig. SI- 2).

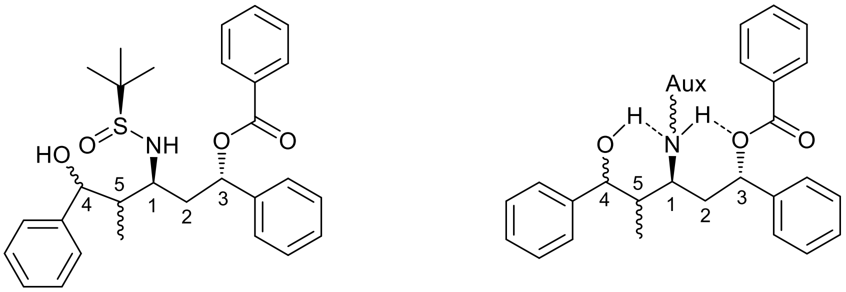

$^{3}J_{H4\text{-}H5}$ = 2.2 Hz   Hydrogen-bonding interaction

Fig. SI-2. $^{3}J_{H\text{-}H}$ coupling constant between H-4 and H-5 and predicted hydrogen-bonding interaction

Analysis of the Newman projections for both the *syn* and *anti*-diastereomers enables us to make a prediction about the relative stereochemistry of the 2-butanone double aldol-Tishchenko product **4a**. There are three possible staggered conformations for each diastereomer (60º, 180º, 300º). Analysis of one each of two possible *syn* and *anti*-diastereomers are shown in Fig. SI-3 and Fig. SI-4. For the *syn*-diastereomer, both hydrogen-bonded conformers have a gauche interaction between H-4 and H-5 which signifies a small *J* value (Fig. SI-3). In comparison, the non-hydrogen bonded conformer has an *anti* relationship between H-4 and H-5 and thus a larger *J* value would be expected. Since the two hydrogen-bond conformers have similar predicted *J* values, a smaller *J* value is expected for the *syn*-diastereomer. This correlates with the experimental coupling constant obtained for **4a** which shows a small *J* value between H-4 and H-5 (*J* = 2.2 Hz).

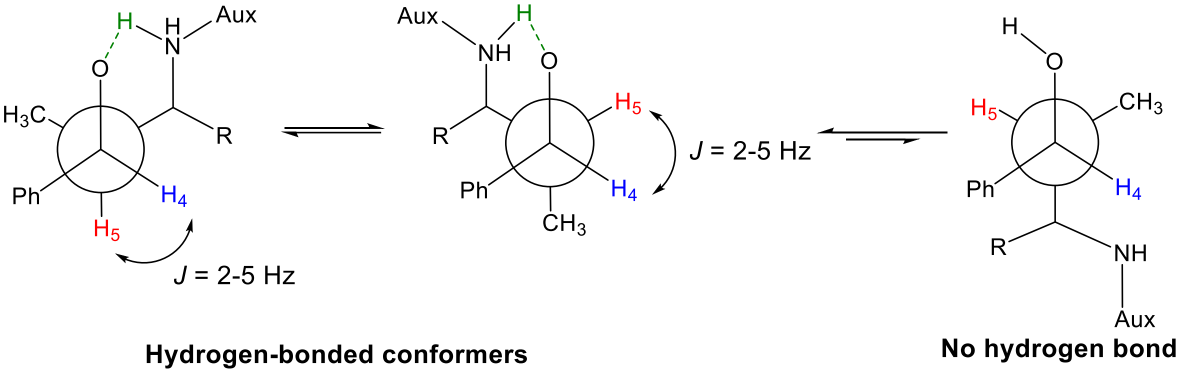

Fig. SI-3. Staggered conformations for *syn*-diastereomer

For the *anti*-diastereomer, one of the hydrogen-bonded conformers has an *anti* relationship while the other hydrogen-bonded conformer has a gauche interaction between H-4 and H-5. A gauche interaction is also present for the non-hydrogen conformer (Fig. SI-4).

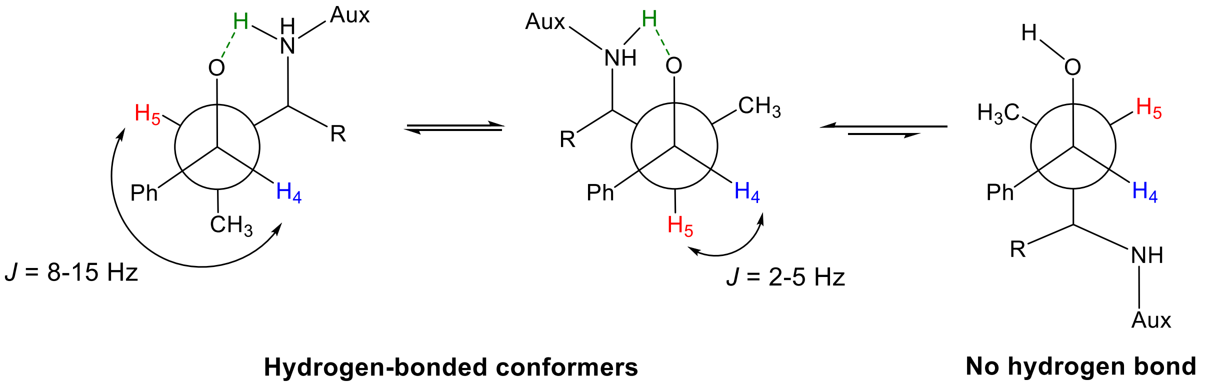

Fig. SI-4. Staggered conformations for *anti*-diastereomer

Using the above conformational analysis, it can be hypothesised that H-4 and H-5 are *syn* to each other. The stereochemical assignment of *syn*-configuration for the 2-butanone double aldol-Tishchenko products is consistent with the aldol stereochemical relationship observed for the cycloheptanone double aldol-Tishchenko products. Thus, the absolute stereochemistry of the major diastereomer for the 2-butanone derived double aldol-Tishchenko series could be either (*S*,*S*,*R*,*S*,*S*) or (*S*,*S*,*S*,*S*,*R*) (Fig. SI-5).

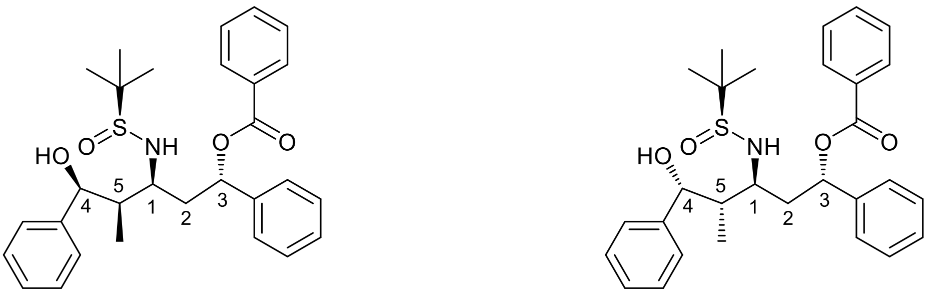

(*S*,*S*,*R*,*S*,*S*)-4a **(*S*,*S*,*S*,*S*,*R*)-4a**

Fig. SI-5. Assignment of relative stereochemistry for the 2-butanone double aldol-Tishchenko series

X. DFT calculations

Density functional theory (DFT) calculations were performed with Gaussian 16.[6] Spartan'16 and CREST were used for conformational searches.[7,8] Molecular geometry optimizations and frequency calculations were performed using the B3LYP functional,[9] augmented with Grimme's D3 empirical dispersion term,[10] and the 6-31G(d) basis set. Frequency calculations confirmed the optimized structures as minima (zero imaginary frequencies) or transition state structures (one imaginary frequency) on the potential energy surface. Intrinsic reaction coordinate (IRC) calculations were performed in order to connect the transition states to the reactants and the products. Single point energies were calculated using M06-2X-D3/6-311+G(d,p),[11] and a quasi-harmonic correction was applied using the GoodVibes program.[12] 3D renderings of optimized structures were generated using PyMol 2.3.2.[13] GaussView 6.0.16 was used to generate initial structures.[14]

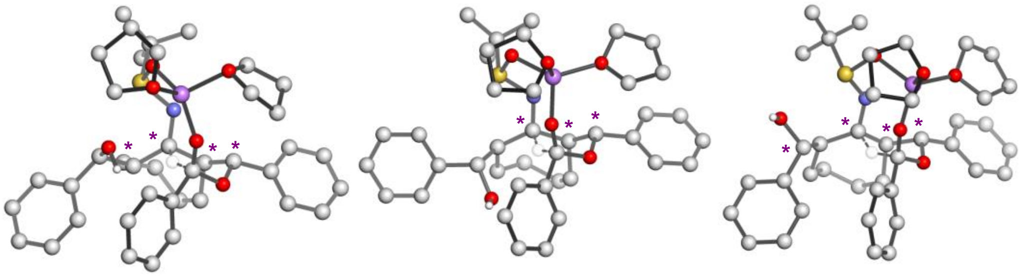

TS-h
$\Delta\Delta G^{\ddagger}$ = 9.4 kcal/mol
(*R,S,S,S,S,S*)
C2, C3, C4, C5

TS-i
$\Delta\Delta G^{\ddagger}$ = 11.3 kcal/mol
(*R,S,S,R,S,S*)
C3, C4, C5

TS-j
$\Delta\Delta G^{\ddagger}$ = 18.0 kcal/mol
(*R,S,S,R,S,R*)
C1, C3, C4, C5

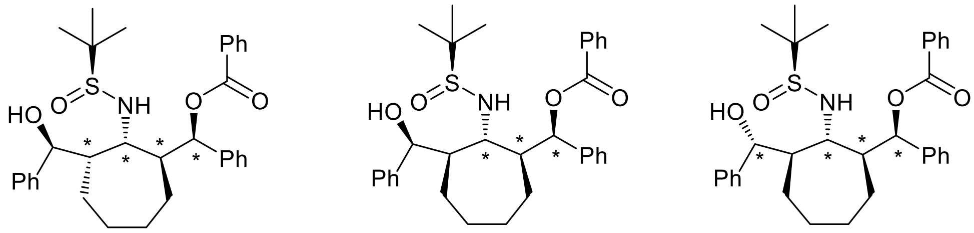

Fig. SI-6. Transition states leading towards the formation of three minor diastereomers of the cycloheptanone series, which differ from the major diastereomer by the stereochemistry at C1, C2, and C3, as well as C3 or C4.

Summary of Energies

Structure	E_SPC (au)	qh-H_SPC (au)	T.qh-S (au)	qh-G(T)_SPC (au)	qh-G(T)_SPC (kcal/mol)	Δqh-G(T)_SPC (kcal/mol)
**Figure 1**						
**TS-a**	-2468.32	-2467.42	0.12274	-2467.54	-1548406.3	0.0

**TS-b**	-2468.32	-2467.42	0.12218	-2467.54	-1548404.6	1.7
**TS-c**	-2468.30	-2467.40	0.12373	-2467.53	-1548394.5	11.8
**TS-d**	-2468.30	-2467.40	0.12298	-2467.52	-1548393.3	13.0
**Figure 2**						
**TS-e**	-2468.32	-2467.42	0.12423	-2467.54	-1548404.9	1.4
**TS-f**	-2468.31	-2467.41	0.12304	-2467.54	-1548401.0	5.3
**TS-g**	-2468.31	-2467.40	0.12308	-2467.53	-1548396.0	10.3
**Figure SI-6**						
**TS-h**	-2468.31	-2467.41	0.12374	-2467.53	-1548396.9	9.4
**TS-i**	-2468.30	-2467.40	0.12409	-2467.53	-1548395.0	11.3
**TS-j**	-2468.29	-2467.39	0.12414	-2467.52	-1548388.3	18.0

For the deprotonation and aldol reactions shown in the sections below and in Fig. SI-7 and Fig. SI-8, DFT calculations were carried out at the B3LYP/6-31G* level of theory. Gibbs free energies are the values at 298.15 K obtained from the frequency calculations.

Deprotonation

The transition structure **TS1a**, in which proton-a is removed, is 8.3 kcal/mol more stable than **TS1b**, in which proton-b is removed (Fig. SI-7). The distances of C-H and H-N are 1.31 and 1.50 Å in **TS1a** and 1.38 and 1.44 Å in **TS1b**, respectively. **Int1a'** is 7.4 kcal/mol more stable than **Int1b'**. In enolate **Int1a'**, the chiral sulfinamide nitrogen and oxygen chelate to the Li^{+} and the lone pairs of the sulfur and the nitrogen atoms face in opposite directions. **TS1a** also has a similar conformation of the sulfinamide moiety and the lone pairs of the sulfur and the nitrogen atoms are *anti* to each other. On the other hand, lone pair repulsion is present in both **TS1b** and enolate **Int1b'**. These results show that deprotonation is facile and leads to a low-energy intermediate.

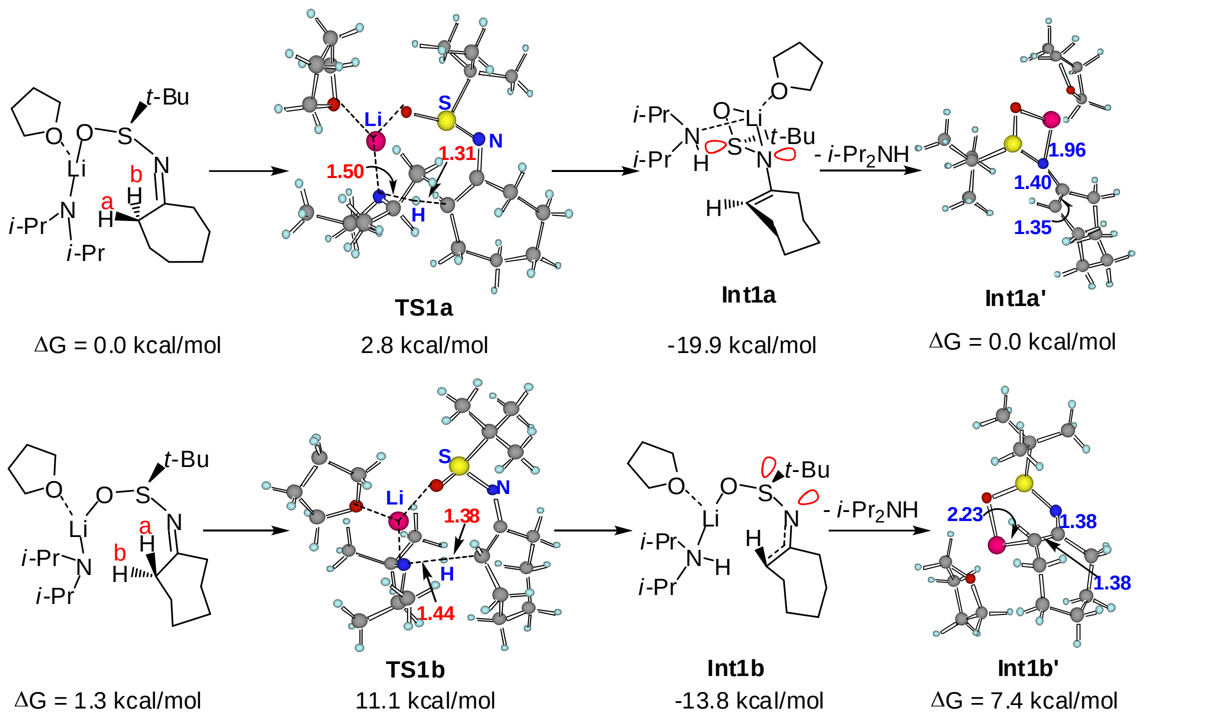

Fig. SI-7. Transition structures for the deprotonation reaction of the cycloheptanone sulfinimine 1a by LDA in THF.

Aldol reaction

In order to calculate the energetics of the initial aldol step, the transition structures for the reaction of both enolates **Int1a'** and **Int1b'** with PhCHO were located (Fig. SI-8). The relative energies of the transition structures **TS2-1** and **TS2-2** from **Int1a'** are lower than those of **TS2-3** and **TS2-4** from **Int1b'** by more than 10 kcal/mol. The activation free energy of **TS2-2** is only 4.9 kcal/mol and lower than that of **TS2-1** by 1.4 kcal/mol, and the *anti*-product **Int2-2** is more stable than the *syn*-product **Int2-1** by 2.0 kcal/mol. If the reaction stops at the aldol step, the product of choice should be the *anti*-product **Int2-2**. This is in agreement with the experimental results from **1a** and furfural (Scheme 8). Due to the low barrier for the aldol reaction and the similar energies between starting materials **Int1** and products **Int2**, the aldol reactions are reversible, which is consistent with experimental results.

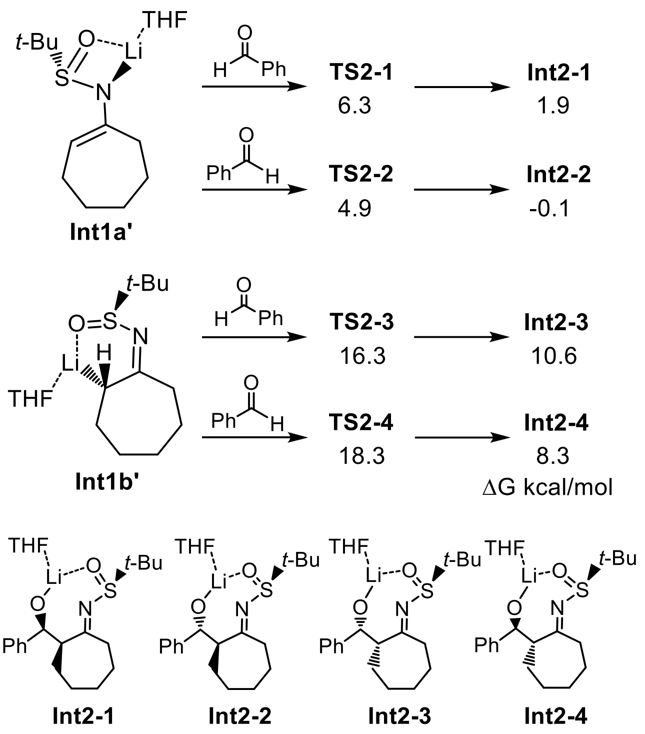

Fig. SI-8. The computed relative Gibbs free-energies for the aldol reaction of **Int1** and PhCHO.

Coordinates (Fig. 1, Fig. 2, Fig. SI-6)

TS-a

C	-0.713881	-3.709388	2.211431
C	0.018136	-3.241038	0.937156
C	-2.147687	-3.193047	2.407676
H	-0.747414	-4.806911	2.205097
C	-2.310721	-1.667710	2.427966
H	-2.539137	-3.608994	3.346022
H	-2.784170	-3.596592	1.606297
H	-3.354759	-1.427145	2.656170
C	-1.933244	-1.018058	1.079587
H	-1.709401	-1.223771	3.231274
H	-2.134354	-1.739026	0.279529
C	-0.431749	-0.674463	1.026819
C	0.600002	-1.821376	1.038093
H	-0.637401	-3.332764	0.063535
H	0.856152	-3.923051	0.756269
H	1.139575	-1.763385	1.992046
Li	1.628826	2.049158	-0.444857
N	-0.150646	0.530881	1.623126
S	1.341314	0.825856	2.300730
C	0.822123	2.139094	3.541532
O	2.246208	1.590117	1.299184
C	2.131509	2.564693	4.220871
C	-0.133969	1.474498	4.537824

C	0.173809	3.322986	2.824254
H	1.913611	3.316862	4.988078
H	2.823334	2.999912	3.493880
H	2.627351	1.717809	4.710406
H	0.010744	4.132230	3.547003
H	-0.793808	3.053730	2.397057
H	0.816748	3.697336	2.022571
H	-0.367761	2.185110	5.339906
H	0.316915	0.587078	4.999003
H	-1.070967	1.178473	4.059006
O	-2.732947	1.211204	1.769230
C	-2.792082	0.237777	0.738978
C	-4.233325	-0.139283	0.464254
H	-2.376639	0.651713	-0.197447
C	-5.252219	0.155273	1.375164
C	-4.553123	-0.816515	-0.720184
C	-5.866052	-1.201640	-0.985012
H	-3.771041	-1.036358	-1.441337
H	-6.096735	-1.722752	-1.911087
C	-6.880533	-0.910579	-0.068478
H	-7.905771	-1.207847	-0.274305
C	-6.568998	-0.229620	1.109319
H	-5.001356	0.695714	2.281377
H	-7.353278	0.005981	1.824700
H	-1.763031	1.281069	1.935218
H	2.101460	-0.533144	0.133987
C	1.678735	-1.523669	-0.062769
C	2.817219	-2.514840	-0.074900
C	2.987504	-3.443593	-1.106262
C	3.732174	-2.499249	0.987116
C	4.055107	-4.343610	-1.074105
H	2.288064	-3.446293	-1.934993
C	4.795775	-3.399844	1.020416
H	3.612032	-1.770002	1.785681
H	4.179063	-5.058597	-1.883419
C	4.960329	-4.326770	-0.012165
H	5.791077	-5.027013	0.009896
H	5.499749	-3.373464	1.848021
O	1.046646	-1.495166	-1.341323
C	0.209840	-0.365475	-1.509846
H	-0.428426	-0.461335	-0.329940
C	-0.910738	-0.701473	-2.463945
O	0.715506	0.800515	-1.501457
C	-1.594487	0.344198	-3.093132
C	-1.319460	-2.022571	-2.687918
C	-2.672665	0.077750	-3.935553
H	-1.263231	1.358809	-2.911873
H	-3.199447	0.898027	-4.415788

C	-3.076393	-1.241009	-4.159823
H	-3.919137	-1.450297	-4.813119
C	-2.394607	-2.289599	-3.536200
H	-0.785075	-2.832084	-2.204013
H	-2.703101	-3.317477	-3.707537
H	-0.104955	-3.431849	3.085050
O	0.599711	3.744287	-0.590694
O	3.321293	2.335104	-1.479366
C	3.442451	1.585057	-2.704384
C	4.538088	2.084747	-0.748263
C	-0.838997	3.582913	-0.476436
C	0.874251	4.536073	-1.759338
C	4.106678	0.257192	-2.302863
H	4.067135	2.156783	-3.406907
H	2.437390	1.467425	-3.110264
H	3.345260	-0.508481	-2.139273
H	4.785831	-0.103465	-3.081307
C	4.845623	0.595145	-0.977527
H	4.347336	2.342755	0.293833
H	5.332193	2.729422	-1.153568
H	4.450722	-0.004348	-0.154171
H	5.922572	0.410780	-1.036990
H	0.787817	5.605363	-1.512255
C	-0.197855	4.104788	-2.757054
H	1.900505	4.317018	-2.061242
H	-1.203276	4.228436	0.329302
H	-1.020791	2.543212	-0.200597
C	-1.434728	3.975077	-1.846516
H	-0.337933	4.818755	-3.574493
H	0.076846	3.133087	-3.180648
H	-1.956913	4.935530	-1.777218
H	-2.155520	3.235141	-2.204705
TS-b			
C	-1.328007	-4.042782	1.485149
C	-0.464995	-3.356594	0.410519
C	-2.737620	-3.468529	1.676736
H	-1.425823	-5.105263	1.225707
C	-2.805217	-1.980068	2.038217
H	-3.248386	-4.048763	2.457232
H	-3.314326	-3.623944	0.753158
H	-3.853569	-1.729222	2.241319
C	-2.285796	-1.043276	0.925348
H	-2.251736	-1.777611	2.965909
H	-2.505747	-1.499034	-0.046740
C	-0.760636	-0.840561	1.023990
C	0.189753	-2.043441	0.868264
H	-1.050346	-3.193476	-0.501871
H	0.342003	-4.042740	0.130901

H	0.646292	-2.223570	1.852118
Li	1.902674	1.733299	0.171188
N	-0.445161	0.223781	1.816187
S	0.960015	0.307617	2.697855
C	0.411521	1.682671	3.860624
O	2.120308	0.948157	1.887449
C	1.654150	2.019031	4.694659
C	-0.710539	1.109183	4.731592
C	-0.056351	2.898026	3.058376
H	1.401190	2.798886	5.422650
H	2.463068	2.385550	4.056555
H	2.017720	1.145641	5.249764
H	-0.299989	3.710311	3.755062
H	-0.951174	2.667409	2.475061
H	0.726024	3.250234	2.380219
H	-1.010649	1.858239	5.474536
H	-0.384168	0.212444	5.272580
H	-1.582355	0.851371	4.124440
H	-3.117843	0.620010	2.032210
C	-3.035482	0.330888	0.973016
C	-4.426178	0.193643	0.388363
O	-2.351626	1.351147	0.249939
C	-4.583135	0.032147	-0.995174
C	-5.563024	0.198313	1.201369
C	-6.836457	0.032525	0.648982
H	-5.452666	0.338209	2.274638
H	-7.710770	0.037497	1.295082
C	-6.983418	-0.130473	-0.728626
H	-7.972795	-0.256182	-1.161285
C	-5.851078	-0.126291	-1.549375
H	-3.706831	0.043175	-1.632434
H	-5.953120	-0.246540	-2.625135
H	1.826610	-0.718682	0.355610
C	1.372348	-1.615701	-0.070649
C	2.462838	-2.650976	-0.194053
C	2.656428	-3.399317	-1.359405
C	3.325681	-2.845798	0.893293
C	3.700395	-4.323833	-1.437678
H	1.998183	-3.236019	-2.205777
C	4.364823	-3.772442	0.817359
H	3.189739	-2.252117	1.794809
H	3.845778	-4.894107	-2.351543
C	4.556859	-4.514019	-0.351754
H	5.370625	-5.231384	-0.415910
H	5.030661	-3.908119	1.665581
O	0.853253	-1.294024	-1.357750
C	0.092052	-0.088940	-1.330988
H	-0.647901	-0.388358	-0.283465

C	-0.932452	-0.107543	-2.436999
O	0.677174	1.002951	-1.025062
C	-1.489708	1.103888	-2.860367
C	-1.388386	-1.307186	-2.998097
C	-2.476163	1.119109	-3.844787
H	-1.156881	2.020908	-2.392360
H	-2.907030	2.064915	-4.162159
C	-2.924443	-0.078679	-4.408402
H	-3.698551	-0.067268	-5.171334
C	-2.378707	-1.291727	-3.980803
H	-0.956817	-2.245170	-2.666954
H	-2.725150	-2.227293	-4.412116
H	-0.787659	-4.014344	2.443320
H	-1.468460	1.388452	0.676492
O	1.509644	3.680383	0.072488
O	3.691482	1.565712	-0.691566
C	0.276970	4.053809	-0.570262
C	2.519531	4.568597	-0.439285
C	3.644860	1.443433	-2.128164
C	4.611758	0.574254	-0.157496
C	3.933836	-0.028704	-2.398343
H	4.406210	2.101171	-2.574922
H	2.649449	1.760335	-2.445497
C	4.993877	-0.350696	-1.329684
H	4.087340	0.056314	0.648470
H	5.477564	1.095482	0.266927
H	3.023506	-0.610435	-2.232451
H	4.286049	-0.211567	-3.418313
H	4.985935	-1.403881	-1.037688
H	5.996242	-0.106344	-1.699930
H	2.529817	5.483311	0.169521
C	2.120059	4.871512	-1.904261
H	3.476254	4.054200	-0.335808
H	-0.117298	4.963405	-0.092887
H	-0.427652	3.233424	-0.435448
C	0.682695	4.300543	-2.025071
H	2.150443	5.947639	-2.101083
H	2.799026	4.387182	-2.611810
H	-0.003794	4.975382	-2.544951
H	0.693900	3.343530	-2.553315
TS-c			
C	-1.597748	3.304586	-2.021466
C	-0.816058	2.921229	-0.758377
C	-2.802403	2.411358	-2.317828
H	-1.948683	4.340295	-1.919743
C	-2.487378	0.919403	-2.477424
H	-3.284934	2.758103	-3.241773
H	-3.548800	2.541775	-1.525681

H	-3.391077	0.421820	-2.847541
C	-2.019295	0.126596	-1.233920
H	-1.728132	0.788793	-3.260260
H	-2.058251	-0.926096	-1.529784
C	-0.499221	0.317404	-0.916149
C	0.148247	1.733290	-0.931562
H	-1.506547	2.731522	0.071191
H	-0.219134	3.786027	-0.451768
H	0.648227	1.844667	-1.902015
Li	1.916254	-2.157839	0.852136
N	0.153643	-0.769762	-1.412154
S	1.805593	-0.805423	-1.679290
C	1.823463	-1.220617	-3.515861
O	2.325218	-2.108746	-1.026130
C	3.290993	-1.511533	-3.850843
C	1.320589	0.026385	-4.248270
C	0.937904	-2.434179	-3.789431
H	3.387744	-1.727806	-4.921902
H	3.652977	-2.374842	-3.285726
H	3.934315	-0.652782	-3.621565
H	0.988234	-2.690464	-4.855254
H	-0.101258	-2.224404	-3.524859
H	1.277622	-3.296984	-3.210166
H	1.371601	-0.135572	-5.331625
H	1.930412	0.908128	-4.013734
H	0.281058	0.242408	-3.984984
H	-2.737401	1.090486	0.589513
C	-2.955305	0.194681	-0.005307
C	-4.438130	0.182928	-0.333650
O	-2.631589	-0.980119	0.749741
C	-5.268428	1.247037	0.030109
C	-5.005681	-0.930609	-0.968735
C	-6.367835	-0.962766	-1.259737
H	-4.370191	-1.776044	-1.216898
H	-6.794506	-1.830432	-1.756469
C	-7.187405	0.114638	-0.907634
H	-8.250351	0.087989	-1.132477
C	-6.635712	1.218048	-0.256885
H	-4.841903	2.104238	0.547339
H	-7.267297	2.053799	0.032975
H	1.970776	0.980968	-0.004653
C	1.273812	1.803136	0.158011
C	2.079616	3.079047	0.132654
C	2.018232	4.021270	1.163904
C	2.930981	3.314483	-0.955675
C	2.789116	5.184515	1.102182
H	1.372372	3.829139	2.013727
C	3.698418	4.476288	-1.018726

H	2.998178	2.574156	-1.750438
H	2.733951	5.909010	1.910755
C	3.627938	5.417859	0.011625
H	4.227208	6.323330	-0.034255
H	4.356191	4.644452	-1.867610
O	0.669406	1.621125	1.431921
C	0.215153	0.278280	1.572968
H	-0.505551	0.209725	0.469491
C	-0.796809	0.213184	2.690367
O	1.075379	-0.651710	1.453074
C	-0.957063	-0.991595	3.384831
C	-1.619234	1.302875	3.000178
C	-1.951658	-1.114700	4.355281
H	-0.309266	-1.825680	3.137170
H	-2.076276	-2.056194	4.883942
C	-2.790947	-0.033646	4.643681
H	-3.571618	-0.132866	5.393213
C	-2.615188	1.178449	3.970069
H	-1.474084	2.240727	2.474554
H	-3.254687	2.026744	4.200248
H	-0.910800	3.300144	-2.881160
H	-2.969767	-0.865391	1.651907
O	0.791587	-3.719599	1.285307
O	3.688998	-2.035421	1.766767
C	1.234600	-4.989121	0.781595
C	-0.551822	-3.536851	0.775153
C	3.689800	-1.145748	2.901713
C	4.770416	-1.597265	0.924348
C	4.062532	0.244494	2.347248
H	4.431736	-1.505850	3.629194
H	2.693057	-1.185331	3.342360
C	4.709607	-0.062841	0.968636
H	4.597726	-2.020950	-0.065631
H	5.719077	-1.974943	1.335372
H	3.167542	0.858123	2.228721
H	4.751041	0.766720	3.018820
H	4.078643	0.308551	0.156419
H	5.700052	0.388289	0.854051
H	-1.252294	-4.035319	1.460302
C	-0.561385	-4.179845	-0.626310
H	-0.758381	-2.468803	0.763604
H	0.786995	-5.794623	1.384359
H	2.321920	-5.026499	0.893662
C	0.740836	-5.023293	-0.670029
H	-1.456402	-4.793068	-0.772075
H	-0.544048	-3.398178	-1.387633
H	0.582847	-6.045148	-1.029150
H	1.479106	-4.532615	-1.308585

TS-d

C	-1.933280	3.647894	-1.481339
C	-0.944397	3.085923	-0.448251
C	-3.255464	2.884980	-1.610254
H	-2.163123	4.687472	-1.210819
C	-3.123328	1.414271	-2.025821
H	-3.877745	3.391116	-2.361856
H	-3.797277	2.945564	-0.661709
H	-4.125929	1.029501	-2.250400
C	-2.483882	0.424720	-1.026201
H	-2.552208	1.346997	-2.963437
H	-2.629877	-0.563287	-1.474180
C	-0.925721	0.509915	-0.999164
C	-0.158806	1.854599	-0.922320
H	-1.463608	2.858028	0.484252
H	-0.211813	3.869838	-0.226660
H	0.207540	2.059400	-1.939480
Li	2.221470	-1.538925	-0.281750
N	-0.437429	-0.520967	-1.745640
S	0.901176	-0.368302	-2.720672
C	0.549248	-1.850660	-3.826405
O	2.226499	-0.762297	-2.004580
C	1.763268	-1.955362	-4.757695
C	-0.729462	-1.532841	-4.607324
C	0.396618	-3.110735	-2.974587
H	1.616727	-2.791828	-5.451573
H	2.677928	-2.131901	-4.185005
H	1.895973	-1.043527	-5.352824
H	0.208219	-3.968546	-3.633036
H	-0.442373	-3.006654	-2.281772
H	1.305032	-3.309487	-2.399399
H	-0.931121	-2.343414	-5.318556
H	-0.631972	-0.601723	-5.179128
H	-1.584322	-1.437696	-3.932738
H	-2.681812	-0.522143	0.867106
C	-3.199127	0.289622	0.338089
C	-4.651895	-0.138489	0.162036
O	-3.087376	1.487016	1.103038
C	-5.706693	0.754075	0.373765
C	-4.946046	-1.450911	-0.231438
C	-6.265177	-1.859543	-0.423080
H	-4.133020	-2.158183	-0.385070
H	-6.476017	-2.881744	-0.727176
C	-7.313632	-0.958897	-0.216698
H	-8.342984	-1.275550	-0.363066
C	-7.029427	0.346973	0.184857
H	-5.480258	1.765180	0.695002
H	-7.839007	1.052928	0.352907

H	1.695807	0.810465	-0.496371
C	1.130038	1.633169	-0.057203
C	2.056432	2.823193	-0.011786
C	2.197483	3.623632	1.126151
C	2.822815	3.114522	-1.148601
C	3.094831	4.693752	1.129549
H	1.614225	3.390100	2.010200
C	3.714832	4.186321	-1.148004
H	2.729278	2.483020	-2.029409
H	3.200396	5.304089	2.022861
C	3.855744	4.979224	-0.005663
H	4.555608	5.810617	-0.000487
H	4.307348	4.396132	-2.034695
O	0.748821	1.258418	1.265641
C	0.173915	-0.042909	1.287602
H	-0.700397	0.150095	0.321830
C	-0.678458	-0.195515	2.526760
O	0.881422	-1.037621	0.910724
C	-1.104395	-1.477656	2.893395
C	-1.091910	0.906371	3.287193
C	-1.938847	-1.659136	3.996276
H	-0.781362	-2.322146	2.295061
H	-2.270056	-2.658847	4.264938
C	-2.350045	-0.558923	4.752422
H	-3.000107	-0.698044	5.612025
C	-1.918267	0.723099	4.398639
H	-0.767202	1.898151	2.995452
H	-2.225449	1.582864	4.988929
H	-1.439037	3.693933	-2.464672
H	-3.135509	1.242759	2.040783
O	2.258700	-3.528138	-0.119440
O	3.964007	-1.039735	0.541997
C	1.099153	-4.091048	0.516265
C	3.401186	-4.194894	0.445414
C	3.940817	-0.902996	1.976612
C	4.698245	0.074542	-0.035010
C	4.008155	0.600095	2.222014
H	4.806087	-1.429976	2.407939
H	3.015431	-1.364461	2.327838
C	4.967888	1.067092	1.112651
H	4.072527	0.490013	-0.827736
H	5.621119	-0.311414	-0.483277
H	3.013746	1.031157	2.082869
H	4.362452	0.850173	3.226982
H	4.782937	2.100652	0.809515
H	6.008170	0.990250	1.449627
H	3.600530	-5.108973	-0.130993
C	3.024466	-4.524944	1.910984

H	4.246420	-3.512531	0.344577
H	0.879302	-5.072253	0.068320
H	0.271170	-3.405228	0.334133
C	1.507594	-4.210140	1.986703
H	3.235297	-5.574306	2.139580
H	3.591586	-3.912954	2.618030
H	0.938810	-4.975929	2.523045
H	1.339293	-3.246821	2.474980

TS-e

C	-0.120516	-1.664245	-3.738835
C	-1.187760	-1.893519	-2.655044
C	1.278107	-2.244732	-3.439926
H	-0.503432	-2.093600	-4.673425
C	2.202629	-1.306207	-2.643957
H	1.775632	-2.458415	-4.394520
H	1.185376	-3.213637	-2.927187
H	3.221795	-1.710655	-2.656936
C	1.826132	-1.063898	-1.171081
H	2.251138	-0.336943	-3.152926
H	1.925606	-2.011448	-0.625311
C	0.360560	-0.604522	-1.006519
C	-0.689905	-1.709474	-1.203785
H	-1.572686	-2.917056	-2.729881
H	-2.040235	-1.236385	-2.852884
H	-0.193825	-2.642546	-0.911176
Li	-1.435218	1.988895	1.102027
N	0.182252	0.713229	-1.353574
S	-1.288750	1.304365	-1.864054
C	-0.659404	2.821683	-2.788292
O	-2.088850	1.918794	-0.686588
C	-1.930511	3.508878	-3.306824
C	0.225822	2.347319	-3.943062
C	0.095872	3.737673	-1.827067
H	-1.653434	4.404385	-3.875519
H	-2.577169	3.809360	-2.477358
H	-2.501770	2.850516	-3.972224
H	0.341102	4.675633	-2.341152
H	1.028276	3.285148	-1.484273
H	-0.517173	3.966598	-0.952753
H	0.526056	3.213188	-4.545968
H	-0.307837	1.651852	-4.601632
H	1.129421	1.854880	-3.575168
O	2.810144	1.201412	-1.158762
C	2.794249	-0.049884	-0.481793
C	4.201609	-0.597756	-0.371957
H	2.414429	0.087200	0.544906
C	5.221868	-0.170441	-1.227318
C	4.484777	-1.572058	0.593918

C	5.763131	-2.119144	0.695727
H	3.701475	-1.896495	1.273857
H	5.967868	-2.871708	1.453441
C	6.778399	-1.693215	-0.164964
H	7.776932	-2.115484	-0.084500
C	6.503533	-0.716007	-1.123492
H	5.000029	0.596702	-1.961219
H	7.289892	-0.374558	-1.792440
H	1.853242	1.359523	-1.352795
H	-2.261056	-0.535735	-0.219035
C	-1.839549	-1.544552	-0.158631
C	-2.945661	-2.555760	-0.333157
C	-2.813893	-3.859419	0.159942
C	-4.106625	-2.207472	-1.032643
C	-3.824504	-4.797744	-0.047790
H	-1.924985	-4.124922	0.723221
C	-5.116852	-3.146755	-1.245696
H	-4.217147	-1.195565	-1.414979
H	-3.713786	-5.805335	0.344674
C	-4.977800	-4.445471	-0.753692
H	-5.764944	-5.177490	-0.913772
H	-6.013461	-2.861470	-1.789648
O	-1.265754	-1.739312	1.141891
C	-0.325751	-0.760762	1.532155
H	0.349272	-0.670565	0.363405
C	0.743147	-1.392068	2.393235
O	-0.713493	0.421037	1.764560
C	1.462479	-0.581645	3.278342
C	1.072310	-2.748807	2.278787
C	2.498389	-1.119434	4.041239
H	1.190435	0.465116	3.356264
H	3.054692	-0.482270	4.723521
C	2.821633	-2.474060	3.928778
H	3.630776	-2.892869	4.521066
C	2.103460	-3.287919	3.047997
H	0.514685	-3.372869	1.589125
H	2.350494	-4.342365	2.956788
H	-0.011801	-0.588721	-3.920913
O	-0.061239	3.332956	1.538262
O	-3.090765	2.290238	2.168229
C	-3.240166	1.358627	3.258339
C	-4.330502	2.234192	1.436811
C	-0.122017	4.699180	1.955110
C	1.328471	2.948580	1.520755
C	-3.976667	0.150701	2.656880
H	-3.826515	1.839232	4.055773
H	-2.239527	1.124928	3.623618
H	-3.253782	-0.595883	2.320441

H	-4.642240	-0.320262	3.386721
C	-4.746336	0.751591	1.447292
H	-4.134949	2.631087	0.440137
H	-5.071947	2.864161	1.950397
H	-4.439639	0.264828	0.518893
H	-5.831071	0.640582	1.540642
H	1.595337	2.531644	2.499975
C	2.128020	4.236218	1.215932
H	1.442233	2.174813	0.763899
H	-0.026410	4.760209	3.050625
H	-1.100998	5.089843	1.665234
C	1.065932	5.364419	1.251266
H	2.905640	4.395001	1.969320
H	2.622155	4.170603	0.243338
H	1.410930	6.260867	1.775289
H	0.779776	5.654685	0.236213
TS-f			
C	0.428522	-2.370715	-3.665437
C	-0.205858	-2.672212	-2.296433
C	1.924484	-2.031821	-3.617824
H	-0.124210	-1.552338	-4.149146
H	0.297158	-3.249109	-4.310630
C	2.297390	-0.763340	-2.844118
H	2.296564	-1.935387	-4.646855
H	2.462544	-2.885061	-3.178039
H	3.382159	-0.623593	-2.913857
C	1.913376	-0.785738	-1.349379
H	1.848731	0.120153	-3.309955
H	2.076594	-1.795702	-0.954025
C	0.430103	-0.422092	-1.104420
C	-0.672895	-1.435687	-1.508064
H	0.486051	-3.273418	-1.690203
H	-1.094307	-3.299279	-2.446555
H	-1.373444	-0.903120	-2.156875
Li	-0.981995	2.244813	1.499432
N	0.220904	0.925023	-1.302357
S	-1.321844	1.551012	-1.376111
C	-1.164932	2.590982	-2.935619
O	-1.465252	2.631014	-0.280852
C	-2.482567	3.370237	-3.035449
C	-1.004512	1.617690	-4.106471
C	0.027320	3.539413	-2.808537
H	-2.481230	3.977693	-3.948579
H	-2.607569	4.032871	-2.174762
H	-3.346358	2.695253	-3.082254
H	0.048775	4.215703	-3.672143
H	0.972312	2.990879	-2.781036
H	-0.058966	4.141974	-1.899306

H	-0.974724	2.177403	-5.049009
H	-1.843805	0.912893	-4.162879
H	-0.076915	1.045764	-4.018428
O	2.831860	1.479955	-1.094805
C	2.827394	0.178520	-0.530694
C	4.249408	-0.338389	-0.423549
H	2.407930	0.226623	0.486733
C	4.516850	-1.516331	0.289126
C	5.312285	0.338553	-1.030252
C	6.616154	-0.152867	-0.927558
H	5.103643	1.252631	-1.574431
H	7.432102	0.385056	-1.404054
C	6.874360	-1.326139	-0.218038
H	7.889301	-1.706906	-0.138022
C	5.817523	-2.007567	0.391649
H	3.702481	-2.052790	0.770300
H	6.006712	-2.921507	0.949429
H	1.866963	1.662496	-1.223150
O	-1.558502	-0.896725	0.751537
C	-1.476471	-1.921074	-0.259438
H	-0.940017	-2.782397	0.155317
C	-2.891173	-2.338048	-0.581151
C	-3.295271	-3.666736	-0.419942
C	-3.821328	-1.393065	-1.037592
C	-4.606357	-4.051731	-0.711863
H	-2.580097	-4.404155	-0.062507
C	-5.131397	-1.773683	-1.320318
H	-3.509374	-0.361446	-1.163083
H	-4.906295	-5.088451	-0.584341
C	-5.527666	-3.104963	-1.159739
H	-6.549413	-3.400696	-1.382364
H	-5.845337	-1.030976	-1.666649
O	-0.215584	0.569187	1.841947
C	-0.332393	-0.597988	1.385618
C	0.253426	-1.739100	2.199872
H	0.421851	-0.648878	0.222925
C	-0.547924	-2.791756	2.657909
C	1.598684	-1.687906	2.581327
C	-0.000970	-3.792037	3.464775
H	-1.600881	-2.815536	2.393892
H	2.212199	-0.854544	2.257826
H	-0.633094	-4.602101	3.819117
C	1.348550	-3.751078	3.817963
H	1.774562	-4.533939	4.439581
C	2.147228	-2.691739	3.376999
H	3.196238	-2.642923	3.656697
O	0.383293	3.520149	2.109783
O	-2.664199	2.313048	2.565597

C	1.103229	3.088689	3.281832
C	1.335211	3.842691	1.056260
C	-3.779215	2.862459	1.842046
C	-3.132130	1.087057	3.165987
C	2.727129	3.449729	1.577949
H	1.248784	4.910228	0.824990
H	1.042433	3.265602	0.177583
C	2.401909	2.498272	2.742275
H	0.472036	2.366793	3.806675
H	1.287249	3.953395	3.936508
H	3.267274	4.329842	1.946327
H	3.318182	2.972739	0.792719
H	3.194757	2.447087	3.494993
H	2.199305	1.490843	2.369702
C	-4.426176	1.646791	1.176445
H	-3.381873	3.594329	1.137014
H	-4.463114	3.357492	2.548682
H	-5.481806	1.808462	0.936818
H	-3.887733	1.429228	0.250949
C	-4.203158	0.512643	2.208008
H	-3.556120	1.319073	4.152881
H	-2.259436	0.444762	3.290415
H	-5.122698	0.274312	2.752243
H	-3.848721	-0.395677	1.718225

TS-g

C	-0.624062	0.197693	-1.029918
C	0.436803	-0.732541	-1.683316
C	1.481881	-1.201814	-0.620509
C	-0.013238	-1.028071	1.198834
O	0.811783	-1.897385	0.425776
O	0.544517	-0.094967	1.878953
Li	1.958166	1.117863	1.707458
H	-0.608752	-0.443241	0.223743
O	2.203391	1.757098	-0.128847
C	-1.121156	-1.845853	1.829555
C	-1.825277	-1.312365	2.914867
C	-1.490227	-3.099313	1.323890
C	-2.879276	-2.020792	3.490647
H	-1.538285	-0.338977	3.296630
C	-2.536573	-3.814058	1.909049
H	-0.946381	-3.511413	0.481539
C	-3.235848	-3.276368	2.992389
H	-3.423950	-1.592979	4.327956
H	-2.809498	-4.789355	1.514422
H	-4.057414	-3.828471	3.440821
N	-0.431944	1.517281	-0.806577
S	0.881510	2.474303	-0.518478
C	1.303136	3.256955	-2.206733

C	2.002969	2.257784	-3.122275
H	1.305904	1.511733	-3.511924
H	2.436981	2.784711	-3.981300
H	2.810907	1.750323	-2.586308
C	0.001144	3.789958	-2.806445
H	-0.503277	4.483321	-2.122911
H	-0.693373	2.977720	-3.037210
H	0.222633	4.334044	-3.733124
C	2.256081	4.400547	-1.829248
H	2.529471	4.961628	-2.730937
H	1.785898	5.101106	-1.128705
H	3.172891	4.014879	-1.372199
C	-2.119063	-0.110377	-1.281411
C	-2.554222	0.220154	-2.724490
H	-3.490979	-0.315419	-2.920141
H	-2.789326	1.289193	-2.771691
C	-3.029350	0.609396	-0.232338
H	-2.610660	0.374891	0.760071
C	2.577416	-2.068859	-1.192548
C	3.550210	-1.476128	-2.008763
C	2.665703	-3.437803	-0.918703
C	4.593237	-2.234449	-2.539065
H	3.494980	-0.409988	-2.213565
C	3.712574	-4.197546	-1.445386
H	1.921672	-3.895460	-0.276097
C	4.679314	-3.600378	-2.256399
H	5.343628	-1.757997	-3.164605
H	3.773346	-5.258712	-1.217943
H	5.494834	-4.192439	-2.662935
H	1.946216	-0.301461	-0.217403
H	1.003774	-0.122628	-2.389813
O	3.618929	0.268919	2.262639
C	4.620891	-0.089185	1.276142
C	3.437538	-0.832832	3.177997
C	4.906104	-1.593233	1.461274
H	4.204804	0.148586	0.293926
H	5.510537	0.530313	1.438437
C	3.737276	-2.071935	2.341741
H	4.135038	-0.723553	4.021378
H	2.408038	-0.782177	3.539597
H	5.860477	-1.739772	1.979890
H	4.953107	-2.120476	0.505219
H	3.992019	-2.944268	2.951625
H	2.863598	-2.312926	1.730511
O	1.354884	2.628352	2.848216
C	1.552837	4.010548	2.494691
C	0.051616	2.535891	3.455228
C	0.144617	4.601096	2.228769

H	2.053890	4.522394	3.327216
H	2.212515	4.026325	1.623989
C	-0.822619	3.448826	2.596730
H	-0.229173	1.484174	3.431742
H	0.111653	2.889160	4.496125
H	0.025809	4.901749	1.185012
H	-0.026536	5.482112	2.855633
H	-1.150322	2.910491	1.702376
H	-1.713388	3.797516	3.127576
H	-2.259250	-1.180898	-1.110579
C	-4.441530	0.059444	-0.280950
C	-5.487648	0.783099	-0.861521
C	-4.703309	-1.212065	0.245036
C	-6.772864	0.239075	-0.922035
H	-5.283972	1.774209	-1.251465
C	-5.985311	-1.756114	0.183520
H	-3.900871	-1.774792	0.711946
C	-7.026559	-1.031875	-0.402942
H	-7.578896	0.812122	-1.374224
H	-6.170789	-2.742411	0.602118
H	-8.028041	-1.452428	-0.449126
O	-3.059147	2.014534	-0.421544
H	-2.107729	2.244072	-0.522893
C	-0.188444	-1.918275	-2.439391
H	0.609968	-2.624521	-2.687897
H	-0.853041	-2.462131	-1.761763
C	-0.926669	-1.534520	-3.748747
H	-0.222441	-1.629614	-4.586039
H	-1.721440	-2.268230	-3.937415
C	-1.518393	-0.116029	-3.803127
H	-1.975028	0.034945	-4.790087
H	-0.699945	0.613096	-3.746029
TS-h			
C	0.138324	-0.751464	1.048477
C	-0.892180	-1.920561	1.068266
C	-1.938314	-1.642033	-0.052661
C	-0.400898	-0.624753	-1.511819
O	-1.288507	-1.716218	-1.319776
O	-0.890580	0.545316	-1.595252
Li	-0.923474	2.249213	-0.910540
H	0.201905	-0.665431	-0.331033
O	-0.264254	2.822532	0.783530
C	0.700195	-1.037125	-2.465645
C	1.386369	-0.037984	-3.170813
C	1.083307	-2.375847	-2.623891
C	2.460030	-0.371969	-3.998792
H	1.057623	0.991310	-3.063078
C	2.143987	-2.708421	-3.467329

H	0.535622	-3.147173	-2.094622
C	2.843529	-1.706805	-4.147356
H	2.992254	0.410534	-4.532861
H	2.430077	-3.750037	-3.588633
H	3.678288	-1.966331	-4.792827
N	-0.466996	0.357076	1.537469
S	0.349313	1.797783	1.763848
C	-0.425678	2.286483	3.407803
C	-1.947147	2.320349	3.274232
H	-2.337310	1.327493	3.036370
H	-2.390987	2.660126	4.218495
H	-2.242856	3.011956	2.480371
C	0.034783	1.261552	4.447192
H	1.129350	1.209883	4.504959
H	-0.347479	0.264968	4.208012
H	-0.337630	1.547650	5.438456
C	0.132734	3.682563	3.712097
H	-0.262450	4.036840	4.672004
H	1.227505	3.672245	3.781580
H	-0.157162	4.394110	2.933255
C	1.624995	-1.152012	1.227373
C	1.929027	-1.669825	2.651959
H	2.912443	-2.155945	2.620518
H	2.026617	-0.812779	3.331467
C	2.681491	-0.078143	0.832034
H	2.797434	0.618860	1.664536
C	-3.123477	-2.575776	-0.026324
C	-4.050585	-2.462978	1.019290
C	-3.324257	-3.551844	-1.007379
C	-5.153985	-3.312575	1.087385
H	-3.905721	-1.699102	1.780810
C	-4.430193	-4.402454	-0.940483
H	-2.617410	-3.627732	-1.826283
C	-5.346628	-4.288262	0.105753
H	-5.865693	-3.210007	1.902529
H	-4.576200	-5.154894	-1.711398
H	-6.207403	-4.949902	0.154640
H	-2.292597	-0.617612	0.100793
H	-1.444834	-1.815705	2.007193
O	-2.881493	2.639118	-1.118590
C	-3.579953	1.997885	-0.017864
C	-3.397382	2.146341	-2.376718
C	-4.634067	1.075091	-0.647014
H	-2.837681	1.448497	0.568480
H	-4.014951	2.778758	0.615379
C	-4.068105	0.813473	-2.052715
H	-4.114353	2.878401	-2.776090
H	-2.552244	2.052928	-3.063191

H	-5.602637	1.585065	-0.712432
H	-4.774640	0.154755	-0.071897
H	-4.840935	0.544767	-2.779673
H	-3.313759	0.023695	-2.027362
O	-0.034672	3.583831	-2.106921
C	-0.620132	4.895603	-2.065957
C	1.358821	3.743237	-1.754378
C	0.005825	5.556153	-0.834605
H	-0.368028	5.437008	-2.991331
H	-1.702664	4.764018	-2.005645
C	1.434119	4.958159	-0.800429
H	1.686580	2.811625	-1.291515
H	1.932382	3.916855	-2.676043
H	-0.542136	5.248258	0.059242
H	-0.002427	6.648887	-0.898791
H	1.693567	4.639574	0.210645
H	2.185572	5.676730	-1.143972
H	1.778419	-1.990337	0.541124
C	4.032080	-0.719807	0.550578
C	5.116890	-0.517059	1.408988
C	4.211027	-1.526532	-0.581881
C	6.355337	-1.108415	1.147729
H	4.991733	0.110729	2.288331
C	5.446905	-2.111009	-0.851097
H	3.383335	-1.704973	-1.262741
C	6.524269	-1.905745	0.015089
H	7.187870	-0.940625	1.826109
H	5.566324	-2.727300	-1.738506
H	7.488032	-2.363000	-0.192779
O	2.260269	0.748720	-0.253884
H	2.227928	0.221598	-1.068941
C	-0.289603	-3.328913	0.994853
H	-1.096049	-4.038279	0.779734
H	0.385120	-3.390312	0.136023
C	0.438173	-3.773278	2.296153
H	-0.235363	-4.421567	2.870963
H	1.304717	-4.394934	2.034390
C	0.891413	-2.639803	3.233926
H	1.308130	-3.085446	4.146600
H	0.012783	-2.069782	3.559399
TS-i			
C	-0.359339	-0.754942	-0.993431
C	0.845309	-1.709854	-1.165729
C	1.981888	-1.196300	-0.231521
C	0.436571	-0.614057	1.482190
O	1.590213	-1.380724	1.128262
O	0.553053	0.659958	1.539327
Li	1.071370	2.174988	0.645884

H	-0.284879	-0.870848	0.452874
O	-0.297137	2.910734	-0.498265
C	-0.288794	-1.306094	2.617170
C	-1.222608	-0.567676	3.354931
C	-0.116726	-2.666455	2.894708
C	-1.992491	-1.186542	4.342181
H	-1.339777	0.487840	3.134063
C	-0.874594	-3.281253	3.891532
H	0.598159	-3.236042	2.312528
C	-1.821506	-2.547994	4.611967
H	-2.722341	-0.607017	4.901932
H	-0.735809	-4.339281	4.098412
H	-2.420518	-3.032328	5.378588
N	0.022764	0.500606	-1.300713
S	-1.032498	1.803790	-1.283169
C	-0.836348	2.369378	-3.073932
C	0.646624	2.618617	-3.349004
H	1.223654	1.697363	-3.231739
H	0.771367	2.991150	-4.373324
H	1.040153	3.366705	-2.654144
C	-1.409524	1.271600	-3.973137
H	-2.462328	1.066921	-3.742946
H	-0.841574	0.341735	-3.870498
H	-1.355190	1.591296	-5.020838
C	-1.649397	3.664608	-3.181715
H	-1.579662	4.059474	-4.202682
H	-2.709807	3.493776	-2.959054
H	-1.267244	4.421244	-2.490157
C	-1.852989	-1.187919	-1.211851
C	-2.165467	-2.559023	-1.868662
H	-2.297067	-3.321356	-1.096199
H	-3.145235	-2.444153	-2.346559
C	-2.709630	-1.016898	0.076120
H	-2.335258	-0.134130	0.614453
C	3.308841	-1.880295	-0.464021
C	3.961076	-1.704518	-1.693209
C	3.927997	-2.656648	0.521267
C	5.204840	-2.286748	-1.932180
H	3.493261	-1.095763	-2.463943
C	5.175495	-3.239966	0.283507
H	3.432384	-2.783453	1.477069
C	5.819021	-3.057506	-0.941079
H	5.697471	-2.134853	-2.889200
H	5.645368	-3.837089	1.061022
H	6.790411	-3.509469	-1.123171
H	2.105107	-0.127575	-0.431147
H	1.211414	-1.567702	-2.190034
O	2.985033	1.983145	0.212589

C	3.840254	2.662789	-0.708514
C	3.828024	1.395856	1.227332
C	5.033988	1.718357	-0.874064
H	3.266376	2.834199	-1.622573
H	4.147538	3.634164	-0.288672
C	5.170255	1.071843	0.528025
H	3.956551	2.122041	2.041174
H	3.296323	0.522709	1.607965
H	5.940211	2.240214	-1.196336
H	4.798354	0.951190	-1.617037
H	6.009487	1.497685	1.086918
H	5.330273	-0.006132	0.449038
O	1.102035	3.615301	2.092968
C	0.516600	4.859270	1.662039
C	0.359979	3.209600	3.257194
C	-0.989065	4.775886	2.008160
H	1.008871	5.685189	2.195344
H	0.709235	4.952512	0.591935
C	-1.096057	3.503520	2.887243
H	0.575093	2.153848	3.423534
H	0.688049	3.804951	4.123772
H	-1.587999	4.674699	1.101457
H	-1.316955	5.671582	2.545869
H	-1.485138	2.671701	2.293227
H	-1.735657	3.638606	3.765159
H	-2.230366	-0.444462	-1.917315
C	-4.180114	-0.786209	-0.253514
C	-4.597555	0.459559	-0.742567
C	-5.124831	-1.804530	-0.098692
C	-5.932932	0.678867	-1.078584
H	-3.867780	1.257239	-0.857689
C	-6.463231	-1.585538	-0.433625
H	-4.798627	-2.763194	0.290722
C	-6.871706	-0.345258	-0.926063
H	-6.242955	1.651253	-1.453387
H	-7.187658	-2.386514	-0.308254
H	-7.913520	-0.174887	-1.184901
O	-2.516492	-2.182203	0.869049
H	-2.621574	-1.940591	1.802376
C	0.519027	-3.189458	-0.938304
H	1.419052	-3.714957	-0.600074
H	-0.211034	-3.277391	-0.126559
C	-0.009017	-3.858935	-2.215813
H	0.819521	-4.001603	-2.923327
H	-0.383707	-4.860727	-1.965869
C	-1.134454	-3.055554	-2.891126
H	-1.627858	-3.687334	-3.640119
H	-0.717779	-2.203447	-3.446467

TS-j

C	-0.445961	-0.396192	-1.267741
C	0.412602	-1.694024	-1.340608
C	1.507792	-1.554826	-0.239035
C	-0.056591	-0.590124	1.290181
O	0.877959	-1.646383	1.040667
O	0.404688	0.592243	1.464778
Li	1.718196	1.768551	0.961168
H	-0.654021	-0.578057	0.155827
O	1.119699	2.896551	-0.445056
C	-1.097607	-1.087053	2.274330
C	-1.801924	-0.146333	3.035606
C	-1.407795	-2.446074	2.409908
C	-2.812338	-0.553359	3.906085
H	-1.550580	0.902357	2.924328
C	-2.418398	-2.853460	3.282673
H	-0.850067	-3.175675	1.834445
C	-3.127688	-1.908905	4.029260
H	-3.358487	0.187179	4.484665
H	-2.651379	-3.910749	3.380779
H	-3.917445	-2.227401	4.704699
N	0.372778	0.658341	-1.410143
S	-0.041557	2.276462	-1.256488
C	0.376700	2.884461	-3.001860
C	1.843299	2.559849	-3.287371
H	2.011576	1.479269	-3.264706
H	2.113999	2.940671	-4.280229
H	2.492045	3.027547	-2.541589
C	-0.559221	2.202774	-4.000849
H	-1.610257	2.344579	-3.730262
H	-0.351723	1.129354	-4.056689
H	-0.396878	2.628307	-4.999149
C	0.136804	4.398266	-2.958894
H	0.379149	4.837390	-3.934703
H	-0.911842	4.630114	-2.736565
H	0.767931	4.871163	-2.200598
C	-1.930997	-0.328477	-1.773605
C	-2.635398	-1.632812	-2.203858
H	-2.944290	-2.235114	-1.343579
H	-3.569313	-1.318597	-2.682054
C	-2.840297	0.594803	-0.880066
H	-2.286309	0.839521	0.036834
C	2.596948	-2.597660	-0.331625
C	3.442006	-2.601830	-1.450778
C	2.814676	-3.539869	0.679435
C	4.485200	-3.520475	-1.555225
H	3.286832	-1.868492	-2.239136
C	3.860899	-4.460708	0.576884

H	2.168725	-3.532696	1.550165
C	4.700553	-4.454504	-0.537749
H	5.134082	-3.504005	-2.427162
H	4.019847	-5.183192	1.373519
H	5.515724	-5.169056	-0.614735
H	1.964270	-0.567470	-0.349628
H	0.927171	-1.680489	-2.309243
O	3.477161	0.953240	0.792358
C	4.589550	1.430865	0.030367
C	3.971232	-0.020803	1.735132
C	5.433510	0.178645	-0.211498
H	4.187200	1.894381	-0.873651
H	5.143031	2.187792	0.608933
C	5.244609	-0.628028	1.096610
H	4.192749	0.485044	2.684401
H	3.165392	-0.739603	1.892953
H	6.481505	0.410982	-0.424206
H	5.026832	-0.381456	-1.058003
H	6.103804	-0.507501	1.764248
H	5.123259	-1.694276	0.891522
O	1.864034	3.089594	2.508731
C	1.802309	4.456284	2.056665
C	0.770279	2.933207	3.429466
C	0.301458	4.821624	1.992878
H	2.342741	5.090039	2.774748
H	2.298498	4.498547	1.086116
C	-0.394656	3.659300	2.749263
H	0.610928	1.862935	3.563106
H	1.035316	3.398785	4.391709
H	-0.034167	4.866316	0.955245
H	0.111775	5.790947	2.465335
H	-0.865336	2.975401	2.037390
H	-1.151060	4.001532	3.462766
H	-1.839461	0.236163	-2.704739
C	-4.185061	0.000053	-0.474140
C	-5.348479	0.309902	-1.189630
C	-4.276910	-0.877750	0.612295
C	-6.577232	-0.242401	-0.823075
H	-5.281224	0.987523	-2.034756
C	-5.502883	-1.432823	0.978087
H	-3.388271	-1.133448	1.174190
C	-6.659567	-1.116135	0.262745
H	-7.470828	0.010183	-1.388569
H	-5.546456	-2.106852	1.829313
H	-7.616498	-1.544638	0.549449
O	-3.039506	1.788055	-1.656205
H	-3.454188	2.447446	-1.077107
C	-0.339990	-3.021662	-1.175351

H	0.354515	-3.774723	-0.786993
H	-1.107851	-2.906641	-0.404740
C	-0.966267	-3.544596	-2.471162
H	-0.177282	-3.907555	-3.143694
H	-1.594054	-4.413280	-2.230665
C	-1.815221	-2.483294	-3.187461
H	-2.482696	-2.975424	-3.905209
H	-1.167980	-1.824050	-3.782975

Coordinates and Energies (Fig. SI-7, Fig. SI-8)

G is the Gibbs free energy at 298.15 K (B3LYP/6-31G*).

**TS1a**, G = -1491.248020 Hartree 1 imaginary frequencies -1002.32					**TS1b**, G = -1491.234649 Hartree 1 imaginary frequencies -1360.18				
6	0	–5.169180	–1.186260	–0.461987	6	0	–4.753940	–1.909896	–0.290543
6	0	–4.582590	–0.006796	–1.251535	6	0	–4.531758	–0.814432	0.758595
6	0	–4.470802	–1.526836	0.864243	6	0	–3.484446	–2.421197	–0.980472
1	0	–6.230380	–0.979292	–0.266076	1	0	–5.270139	–2.755584	0.185055
6	0	–3.001947	–1.972232	0.710449	6	0	–2.687253	–1.349275	–1.748347
1	0	–5.041186	–2.334456	1.344563	1	0	–3.776720	–3.214878	–1.682639
1	0	–4.521584	–0.666412	1.547519	1	0	–2.827912	–2.894357	–0.237670
1	0	–2.719781	–2.562018	1.591227	1	0	–2.021776	–1.870066	–2.446820
6	0	–1.964612	–0.851339	0.559793	6	0	–1.808989	–0.414328	–0.902727
1	0	–2.935132	–2.672768	–0.134641	1	0	–3.388532	–0.784764	–2.387400
1	0	–0.790883	–1.405132	0.359983	1	0	–1.051358	0.071904	–1.522384
1	0	–1.744361	–0.382760	1.528957	1	0	–0.890105	–1.172316	–0.205991
6	0	–2.176419	0.148818	–0.459608	6	0	–2.460455	0.575698	–0.070880
6	0	–3.091071	–0.152109	–1.633950	6	0	–3.960985	0.509393	0.208041
1	0	–5.162367	0.112131	–2.176537	1	0	–5.499698	–0.583097	1.223321
1	0	–2.905763	–1.165783	–2.011764	1	0	–4.485336	0.743591	–0.732216
1	0	–2.849440	0.552234	–2.435179	1	0	–4.187775	1.320564	0.905532
7	0	–1.620210	1.350839	–0.543470	7	0	–1.910600	1.615247	0.528035
16	0	–0.722661	1.895048	0.788290	16	0	–0.308222	2.057044	0.623171
6	0	–0.555112	3.680451	0.226344	6	0	–0.480849	3.823539	–0.054857
8	0	0.731514	1.354249	0.715710	8	0	0.701552	1.409746	–0.372191
6	0	0.298213	4.366116	1.302501	6	0	0.934409	4.412400	–0.012461
6	0	–1.969549	4.270296	0.186540	6	0	–1.422974	4.586998	0.881119
6	0	0.125095	3.728208	–1.142415	6	0	–1.015160	3.756575	–1.485125
1	0	0.417187	5.426876	1.051747	1	0	0.903207	5.457050	–0.344677
1	0	1.292727	3.915380	1.367684	1	0	1.611646	3.863011	–0.672122
1	0	–0.172702	4.305732	2.290896	1	0	1.347026	4.398139	1.003856
1	0	0.271014	4.774009	–1.440961	1	0	–1.057168	4.767737	–1.909560
1	0	–0.485585	3.226843	–1.897004	1	0	–2.023705	3.333593	–1.507604
1	0	1.104386	3.240678	–1.106104	1	0	–0.362537	3.146228	–2.117034
1	0	–1.910521	5.335869	–0.067445	1	0	–1.488382	5.632550	0.553825
1	0	–2.468832	4.187294	1.159766	1	0	–1.054008	4.582342	1.913682
1	0	–2.583064	3.765310	–0.563379	1	0	–2.424571	4.152204	0.873276
1	0	–4.710826	0.927211	–0.687275	1	0	–3.892275	–1.189459	1.567827
1	0	–5.148334	–2.080841	–1.103126	1	0	–5.444003	–1.523948	–1.056824
7	0	0.542370	–2.067517	0.188219	7	0	0.261180	–1.910208	0.252179
3	0	1.442579	–0.322592	0.328300	3	0	1.377722	–0.321694	–0.141759
6	0	0.309422	–2.681226	–1.129915	6	0	0.366368	–2.998225	–0.741825
6	0	0.720924	–3.009870	1.306043	6	0	0.036030	–2.366922	1.634486
6	0	1.207598	–3.868167	–1.528209	6	0	1.304084	–4.171194	–0.393615
6	0	0.434601	–1.594185	–2.210089	6	0	0.820719	–2.412371	–2.090406
1	0	–0.729277	–3.073511	–1.181960	1	0	–0.630062	–3.457451	–0.899037
6	0	2.192522	–3.438269	1.516851	6	0	1.344336	–2.588947	2.427615
1	0	0.125592	–3.928415	1.142703	1	0	–0.520747	–3.323159	1.636588
6	0	0.234078	–2.385243	2.626987	6	0	–0.814789	–1.353402	2.415943

8	0	3.378153	–0.016476	0.268384	1	0	0.771930	–3.167692	–2.884306
6	0	4.384742	–0.770948	–0.423419	1	0	1.862834	–2.067769	–2.027606
6	0	3.970185	1.261083	0.562502	1	0	0.203922	–1.563928	–2.401299
6	0	5.073210	0.241426	–1.357462	1	0	1.290497	–4.906967	–1.208295
1	0	3.883982	–1.595152	–0.933991	1	0	1.001408	–4.691842	0.519602
1	0	5.090726	–1.185232	0.310632	1	0	2.340158	–3.830962	–0.267131
6	0	4.791163	1.617839	–0.689719	1	0	1.134906	–2.968616	3.437369
1	0	4.614507	1.157915	1.447763	1	0	1.877255	–1.633662	2.537930
1	0	3.151188	1.944796	0.786069	1	0	2.019681	–3.293247	1.937209
1	0	5.708531	2.153542	–0.428314	1	0	–0.946163	–1.677887	3.455312
1	0	4.211763	2.259478	–1.359931	1	0	–1.803476	–1.224600	1.973133
1	0	6.142454	0.031634	–1.454701	1	0	–0.328210	–0.369432	2.436717
1	0	4.635670	0.202037	–2.359255	8	0	3.319910	–0.205303	–0.348358
1	0	2.278715	–4.161215	2.339937	6	0	4.323365	–1.141058	0.118738
1	0	2.799742	–2.560658	1.776113	6	0	3.945584	1.035052	–0.754691
1	0	2.628203	–3.893019	0.625146	6	0	5.300136	1.040311	–0.051193
1	0	0.470191	–3.039233	3.474941	1	0	3.272304	1.844707	–0.469334
1	0	–0.843966	–2.209230	2.632299	1	0	4.061481	1.034753	–1.847305
1	0	0.731879	–1.421641	2.805326	1	0	5.190858	1.383926	0.984305
1	0	0.126030	–1.970588	–3.193450	1	0	6.030523	1.683935	–0.550452
1	0	1.480944	–1.265168	–2.295890	6	0	5.676619	–0.449483	–0.090921
1	0	–0.172643	–0.713669	–1.979811	1	0	4.221466	–2.073543	–0.444333
1	0	0.914027	–4.235562	–2.520284	1	0	4.124962	–1.349051	1.175758
1	0	1.123279	–4.708188	–0.832220	1	0	6.407803	–0.733325	0.671484
1	0	2.263446	–3.571988	–1.581171	1	0	6.093119	–0.708479	–1.071159

**Int1a'**, G = -1199.0376650 Hartree<br>0 imaginary frequencies					**Int1b'**, G = -1199.025870 Hartree<br>0 imaginary frequencies				
6	0	4.047843	–2.669081	0.615553	6	0	1.958166	3.669955	–0.185754
6	0	2.575295	–2.502178	1.022488	6	0	1.509440	2.790067	–1.361243
6	0	4.768262	–1.396557	0.146714	6	0	1.755261	3.073900	1.213353
1	0	4.598972	–3.098335	1.464307	1	0	3.020182	3.921767	–0.316060
6	0	4.145097	–0.738190	–1.102339	6	0	0.290347	2.744338	1.565358
1	0	5.814000	–1.659761	–0.070015	1	0	2.143535	3.792729	1.949096
1	0	4.791116	–0.660808	0.962459	1	0	2.363213	2.162377	1.320973
1	0	4.874018	–0.031009	–1.517422	1	0	0.230773	2.588728	2.649992
6	0	2.851479	0.008326	–0.860417	6	0	–0.307319	1.520044	0.894787
1	0	4.024446	–1.518425	–1.873797	1	0	–0.315977	3.645755	1.368518
1	0	2.875838	1.078473	–1.052177	1	0	–0.799650	0.816650	1.561948
6	0	1.695597	–0.557262	–0.438462	6	0	–0.488686	1.377510	–0.465501
6	0	1.631124	–2.044125	–0.107571	6	0	0.001258	2.475301	–1.409703
1	0	2.219126	–3.471764	1.399109	1	0	1.779452	3.306667	–2.292344
1	0	1.845706	–2.634128	–1.012544	1	0	–0.557831	3.399804	–1.197891
1	0	0.599258	–2.280272	0.178764	1	0	–0.271886	2.162211	–2.420442
7	0	0.460890	0.068944	–0.218645	7	0	–1.010770	0.327274	–1.194086
16	0	0.179306	1.580680	–0.865250	16	0	–1.637590	–1.070621	–0.598481
6	0	0.234923	2.756762	0.624183	6	0	–3.405170	–0.734707	0.062555
8	0	–1.359065	1.484040	–1.149741	8	0	–0.854807	–1.571482	0.676304
6	0	–0.237071	4.117364	0.097609	6	0	–3.965627	–2.140043	0.325368
6	0	1.693042	2.808900	1.090811	6	0	–4.155013	–0.041729	–1.078339
6	0	–0.690448	2.228497	1.717824	6	0	–3.418459	0.095741	1.344752
1	0	–0.202415	4.862140	0.903271	1	0	–5.010833	–2.065384	0.652227
1	0	–1.264050	4.057616	–0.274818	1	0	–3.397548	–2.650350	1.109577
1	0	0.403499	4.477228	–0.716798	1	0	–3.940458	–2.760632	–0.578083
1	0	–0.677295	2.910548	2.577468	1	0	–4.441223	0.132391	1.745321
1	0	–0.367597	1.238391	2.055060	1	0	–3.086016	1.121152	1.165469
1	0	–1.719766	2.159720	1.350834	1	0	–2.774886	–0.356041	2.106290
1	0	1.781549	3.488605	1.948078	1	0	–5.209688	0.087086	–0.802182
1	0	2.355900	3.183905	0.302045	1	0	–4.120830	–0.633287	–2.001009
1	0	2.051196	1.821088	1.395918	1	0	–3.729596	0.942513	–1.293893
1	0	2.495212	–1.795519	1.860089	1	0	2.075916	1.846461	–1.364242
1	0	4.101838	–3.418425	–0.189914	1	0	1.412052	4.624087	–0.240396
3	0	–1.426761	–0.303499	–0.609945	3	0	0.640317	–0.479826	0.661316
8	0	–3.041762	–1.292728	–0.465008	8	0	2.316559	–1.386530	0.644784
6	0	–3.401452	–2.241516	0.573174	6	0	3.551304	–0.968624	0.006282
6	0	–4.227014	–0.606367	–0.952618	6	0	2.202253	–2.836910	0.608315
6	0	–4.932320	–2.218199	0.649133	6	0	3.104799	–3.261846	–0.544891

1	0	−2.938622	−1.910647	1.510511	1	0	1.142168	−3.066845	0.481494
1	0	−2.992508	−3.219787	0.304171	1	0	2.553226	−3.240070	1.567328
6	0	−5.271687	−0.806946	0.141502	1	0	2.582376	−3.138394	−1.500532
1	0	−4.534350	−1.069583	−1.898645	1	0	3.428237	−4.303535	−0.461995
1	0	−3.950130	0.435030	−1.134422	6	0	4.263763	−2.258334	−0.423876
1	0	−6.294197	−0.719855	−0.236861	1	0	4.128658	−0.371840	0.719119
1	0	−5.136295	−0.065976	0.937811	1	0	3.289635	−0.337593	−0.850715
1	0	−5.361021	−2.975490	−0.016979	1	0	4.822847	−2.128182	−1.354832
1	0	−5.297695	−2.413020	1.661350	1	0	4.968097	−2.583621	0.350145
**TS2-1**, G = -1544.521779 Hartree<br>1 imaginary frequencies -133.18					**TS2-2**, G = -1544.523928 Hartree<br>1 imaginary frequencies -187.46				
6	0	−4.032395	−2.538864	0.238613	6	0	−1.960661	3.048153	−3.021745
6	0	−2.907458	−2.442419	1.281741	6	0	−0.451986	2.841753	−2.819661
6	0	−3.615920	−2.390769	−1.234889	6	0	−2.856235	2.755381	−1.806727
1	0	−4.543582	−3.503079	0.367539	1	0	−2.127927	4.084110	−3.347391
6	0	−3.017227	−1.008960	−1.584743	6	0	−2.838679	1.280358	−1.349812
1	0	−4.509934	−2.553325	−1.853625	1	0	−3.886337	3.027830	−2.076148
1	0	−2.902537	−3.180783	−1.507913	1	0	−2.575144	3.404206	−0.965370
1	0	−3.093668	−0.855738	−2.669199	1	0	−3.739037	1.080500	−0.758176
6	0	−1.566407	−0.807234	−1.187107	6	0	−1.639763	0.857675	−0.514868
1	0	−3.661789	−0.242338	−1.129173	1	0	−2.933854	0.649783	−2.249298
1	0	−0.846997	−0.962370	−1.991509	1	0	−1.819540	0.881667	0.557733
6	0	−1.109535	−1.102436	0.107745	6	0	−0.320189	1.161312	−0.915210
6	0	−2.105411	−1.120975	1.254774	6	0	−0.036428	1.418421	−2.384340
1	0	−3.355648	−2.554901	2.278201	1	0	0.056442	3.074452	−3.764950
1	0	−2.798080	−0.273524	1.187127	1	0	−0.559832	0.677942	−3.005472
1	0	−1.541172	−1.017606	2.186558	1	0	1.037957	1.287894	−2.544486
7	0	0.148483	−1.370203	0.506633	7	0	0.793101	1.155000	−0.169145
16	0	1.213325	−1.989070	−0.638640	16	0	0.625430	1.125472	1.526922
6	0	2.275519	−3.008189	0.539691	6	0	1.979947	2.364963	1.955954
8	0	2.219759	−0.908123	−1.134393	8	0	1.213843	−0.237251	1.973225
6	0	3.380935	−3.617119	−0.333767	6	0	2.033896	2.373087	3.490305
6	0	1.373314	−4.097193	1.129176	6	0	1.532129	3.722479	1.405499
6	0	2.856404	−2.103473	1.626708	6	0	3.309748	1.909596	1.356018
1	0	4.036277	−4.240924	0.286447	1	0	2.796659	3.086174	3.826253
1	0	3.986847	−2.837066	−0.802917	1	0	2.291498	1.383757	3.879699
1	0	2.964542	−4.251986	−1.125134	1	0	1.075076	2.677413	3.926796
1	0	3.488631	−2.697408	2.299123	1	0	4.101981	2.610033	1.649194
1	0	2.058353	−1.638621	2.211094	1	0	3.254136	1.877778	0.264721
1	0	3.474331	−1.315395	1.185071	1	0	3.577114	0.915203	1.726978
1	0	1.972816	−4.768863	1.756258	1	0	2.247830	4.495324	1.711752
1	0	0.906937	−4.703457	0.342832	1	0	0.546918	4.010343	1.792635
1	0	0.582848	−3.659134	1.743595	1	0	1.485967	3.708529	0.312999
1	0	−2.209434	−3.282617	1.162143	1	0	−0.070541	3.561762	−2.082247
1	0	−4.783064	−1.765571	0.462799	1	0	−2.292408	2.411122	−3.856061
3	0	1.756578	0.853905	−1.185441	3	0	1.231037	−1.298034	0.411180
8	0	3.177503	2.149261	−0.988244	8	0	2.686710	−2.417142	−0.212067
6	0	2.837080	3.480309	−0.518826	6	0	2.412640	−3.255958	−1.359476
6	0	4.421269	1.737962	−0.387884	6	0	4.080086	−2.020338	−0.211810
6	0	3.781999	3.778550	0.665078	6	0	3.467043	−2.853335	−2.385813
1	0	1.779905	3.454288	−0.242489	1	0	1.379540	−3.060839	−1.652640
1	0	2.967244	4.188950	−1.344115	1	0	2.517883	−4.309790	−1.066454
6	0	4.412202	2.408281	0.983226	6	0	4.695813	−2.609381	−1.492700
1	0	5.260824	2.088637	−1.005284	1	0	4.554009	−2.395277	0.701294
1	0	4.419171	0.645860	−0.368484	1	0	4.112777	−0.925750	−0.198691
1	0	5.410214	2.489002	1.424267	1	0	5.199308	−3.558234	−1.275218
1	0	3.777552	1.837601	1.670659	1	0	5.430724	−1.936443	−1.943941
1	0	4.556353	4.493492	0.366563	1	0	3.634450	−3.623317	−3.144998
1	0	3.249037	4.205431	1.519331	1	0	3.165589	−1.929458	−2.892940
8	0	0.106545	1.555722	−1.160692	8	0	−0.303875	−1.671887	−0.479912
6	0	−1.138925	1.409869	−1.321654	6	0	−1.493002	−1.274890	−0.702040
1	0	−1.536814	1.291346	−2.341786	1	0	−1.829739	−1.215692	−1.751883
6	0	−2.096939	2.029853	−0.365737	6	0	−2.591664	−1.643862	0.249755
6	0	−3.410084	2.325273	−0.760647	6	0	−3.912432	−1.771998	−0.202730
6	0	−1.677993	2.394253	0.924051	6	0	−2.306288	−1.913376	1.597274
1	0	−3.733148	2.079076	−1.769374	1	0	−4.138530	−1.586421	−1.250355

6	0	−2.559493	3.017788	1.802473	6	0	−3.324949	−2.284726	2.472077
1	0	−0.656416	2.175401	1.218734	1	0	−1.282334	−1.821179	1.947747
1	0	−2.226171	3.290629	2.800696	1	0	−3.093057	−2.486532	3.514937
6	0	−3.871434	3.298081	1.404207	6	0	−4.640819	−2.404514	2.014073
1	0	−4.556984	3.788627	2.090240	1	0	−5.432558	−2.700180	2.697666
6	0	−4.293272	2.953375	0.119169	6	0	−4.931561	−2.149676	0.672643
1	0	−5.306578	3.180518	−0.201889	1	0	−5.950016	−2.251663	0.306078

**TS2-3**, G = -1544.505826 Hartree<br>1 imaginary frequencies -75.84					**TS2-4**, G = -1544.502604 Hartree<br>1 imaginary frequencies -159.51				
6	0	−4.648081	0.138244	−1.778281	6	0	3.594237	2.380544	−2.065388
6	0	−4.258819	0.587397	−0.364346	6	0	2.245295	2.207397	−2.772236
6	0	−3.602567	−0.718847	−2.497857	6	0	3.856830	1.377387	−0.939285
1	0	−5.589722	−0.426275	−1.724302	1	0	4.400592	2.294055	−2.807154
6	0	−2.246766	−0.033015	−2.743630	6	0	2.872787	1.420939	0.244579
1	0	−4.013574	−1.023458	−3.470994	1	0	4.866744	1.553875	−0.543105
1	0	−3.442267	−1.643620	−1.929399	1	0	3.872275	0.366282	−1.363715
1	0	−1.675372	−0.672968	−3.430392	1	0	3.279466	0.771328	1.028644
6	0	−1.347470	0.247691	−1.549660	6	0	1.427124	0.994126	0.022240
1	0	−2.437596	0.894516	−3.314283	1	0	2.903171	2.440024	0.670513
1	0	−0.296035	0.233197	−1.804485	1	0	0.960337	0.568479	0.903806
6	0	−1.679877	1.134105	−0.509171	6	0	0.537068	1.677314	−0.823120
6	0	−3.115610	1.613770	−0.291861	6	0	1.018336	2.606071	−1.937343
1	0	−5.139845	1.053874	0.097768	1	0	2.243156	2.837252	−3.672203
1	0	−3.310150	2.404577	−1.035509	1	0	1.219726	3.587939	−1.476549
1	0	−3.135147	2.105620	0.685108	1	0	0.160771	2.757002	−2.599446
7	0	−0.860563	1.702070	0.383806	7	0	−0.807678	1.622035	−0.822359
16	0	0.771208	1.570377	0.578413	16	0	−1.864412	1.116544	0.337879
6	0	1.178634	3.421072	0.717677	6	0	−1.970751	2.593477	1.548790
8	0	1.636004	1.159455	−0.662983	8	0	−1.413359	−0.046571	1.292886
6	0	2.695665	3.487908	0.928537	6	0	−3.100682	2.214452	2.515137
6	0	0.425981	3.970390	1.932908	6	0	−2.341674	3.828177	0.723101
6	0	0.760502	4.112451	−0.579306	6	0	−0.650321	2.774197	2.295596
1	0	2.999860	4.533070	1.063051	1	0	−3.268732	3.035777	3.223397
1	0	3.232121	3.080777	0.067166	1	0	−2.848998	1.315226	3.084380
1	0	3.001916	2.934661	1.825351	1	0	−4.042679	2.036908	1.981901
1	0	1.030944	5.175244	−0.534915	1	0	−0.763550	3.563029	3.051654
1	0	−0.320678	4.036893	−0.727359	1	0	0.158044	3.067535	1.619988
1	0	1.264841	3.662799	−1.440000	1	0	−0.361791	1.850099	2.804425
1	0	0.679312	5.029741	2.069109	1	0	−2.494052	4.683745	1.393787
1	0	0.703677	3.439892	2.851810	1	0	−3.270708	3.672664	0.161920
1	0	−0.653976	3.880321	1.797738	1	0	−1.554560	4.081619	0.008492
1	0	−4.015718	−0.284725	0.254608	1	0	2.136410	1.172951	−3.126935
1	0	−4.862623	1.030206	−2.387702	1	0	3.656600	3.404855	−1.666188
3	0	1.887623	−0.625630	−1.008185	3	0	−1.201717	−1.581716	0.280093
8	0	3.661093	−1.332420	−1.222244	8	0	−2.699979	−2.667605	−0.233153
6	0	3.888551	−2.756068	−1.030392	6	0	−2.698618	−3.143295	−1.609030
6	0	4.773176	−0.584057	−0.684058	6	0	−4.022949	−2.195440	0.099295
6	0	5.326595	−1.473595	0.425115	6	0	−4.557430	−1.632583	−1.213922
1	0	4.379338	0.378746	−0.350206	1	0	−3.908206	−1.459325	0.898327
1	0	5.515434	−0.415250	−1.476547	1	0	−4.629938	−3.036967	0.462438
1	0	4.710130	−1.376735	1.326316	1	0	−4.133654	−0.636846	−1.383874
1	0	6.360356	−1.230290	0.687656	1	0	−5.648537	−1.552153	−1.228616
6	0	5.173981	−2.875871	−0.191619	6	0	−4.015611	−2.645393	−2.242068
1	0	3.977230	−3.231036	−2.012771	1	0	−2.626304	−4.236259	−1.599905
1	0	3.005308	−3.156516	−0.524485	1	0	−1.803489	−2.731053	−2.081130
1	0	5.102392	−3.667059	0.559947	1	0	−3.852141	−2.196176	−3.225417
1	0	6.032265	−3.101722	−0.834183	1	0	−4.718680	−3.476001	−2.367608
8	0	0.582314	−1.822018	−0.645474	8	0	0.154667	−1.485893	−0.922833
6	0	−0.676208	−1.915456	−0.614510	6	0	1.374038	−1.172538	−0.833484
6	0	−1.412633	−2.023364	0.664163	6	0	2.249883	−1.763938	0.223550
1	0	−1.204146	−2.310228	−1.494458	1	0	1.904367	−0.876799	−1.748649
6	0	−0.858895	−1.541552	1.861417	6	0	1.775222	−2.027983	1.519118
6	0	−1.535411	−1.708381	3.067520	6	0	2.591645	−2.649562	2.460262
1	0	0.101447	−1.037962	1.833546	1	0	0.775232	−1.707431	1.800244
1	0	−1.103213	−1.320096	3.986188	1	0	2.214185	−2.835812	3.462475
6	0	−2.764227	−2.373022	3.100956	6	0	3.895173	−3.028606	2.122210

1	0	−3.288477	−2.504364	4.043914	1	0	4.529720	−3.516282	2.857385
6	0	−3.314522	−2.873927	1.917411	6	0	4.375882	−2.775491	0.837340
1	0	−4.262471	−3.405332	1.938716	1	0	5.385173	−3.071860	0.563690
6	0	−2.643930	−2.697927	0.709293	6	0	3.558889	−2.145356	−0.102723
1	0	−3.067514	−3.097943	−0.208933	1	0	3.932663	−1.964305	−1.107688
**Int2-1**, G = -1544.528746 Hartree<br>0 imaginary frequencies					**Int2-2**, G = -1544.531875 Hartree<br>0 imaginary frequencies				
6	0	−3.039754	3.620145	0.360992	6	0	2.021571	−3.728072	2.143773
6	0	−2.804164	2.569831	1.454473	6	0	2.428904	−2.323690	2.609415
6	0	−2.839335	3.137706	−1.085296	6	0	1.480844	−3.828609	0.706495
1	0	−4.059580	4.013126	0.471821	1	0	2.890274	−4.391836	2.250083
6	0	−1.385191	2.749750	−1.423098	6	0	0.114019	−3.143996	0.495822
1	0	−3.149431	3.950374	−1.755421	1	0	1.374201	−4.893966	0.463992
1	0	−3.514212	2.295627	−1.300254	1	0	2.219402	−3.430523	−0.005229
1	0	−1.224350	2.869083	−2.502113	1	0	−0.406042	−3.627635	−0.337081
6	0	−0.970138	1.306199	−1.082050	6	0	0.130650	−1.629746	0.186437
1	0	−0.708397	3.467173	−0.941768	1	0	−0.510525	−3.335958	1.379735
1	0	−1.451260	0.638453	−1.803692	1	0	0.329773	−1.491481	−0.881020
6	0	−1.335349	0.850306	0.300915	6	0	1.153153	−0.863305	0.976539
6	0	−1.420356	1.877387	1.416249	6	0	1.345587	−1.233898	2.435698
1	0	−2.911838	3.054390	2.433453	1	0	2.701627	−2.369094	3.671652
1	0	−0.628642	2.626211	1.305108	1	0	0.395118	−1.575157	2.864427
1	0	−1.258925	1.351449	2.361470	1	0	1.656857	−0.332728	2.971622
7	0	−1.595828	−0.359585	0.694170	7	0	1.917120	0.108379	0.584341
16	0	−1.665517	−1.647294	−0.473162	16	0	1.834120	0.634131	−1.069846
6	0	−3.179040	−2.504537	0.248099	6	0	3.652925	1.077047	−1.246855
8	0	−0.525946	−2.623478	−0.120285	8	0	1.129398	2.002142	−1.053797
6	0	−3.360897	−3.758851	−0.619964	6	0	3.772948	1.659292	−2.663177
6	0	−4.364862	−1.546987	0.087616	6	0	4.452196	−0.223593	−1.111778
6	0	−2.925133	−2.874010	1.710372	6	0	4.038512	2.110390	−0.187240
1	0	−4.238703	−4.317231	−0.273144	1	0	4.815940	1.939124	−2.853080
1	0	−2.487524	−4.413723	−0.552416	1	0	3.150952	2.552097	−2.775022
1	0	−3.521852	−3.502888	−1.674219	1	0	3.476294	0.931667	−3.428113
1	0	−3.784948	−3.436251	2.096399	1	0	5.080860	2.415553	−0.343350
1	0	−2.783723	−1.980744	2.323166	1	0	3.937852	1.698075	0.819235
1	0	−2.033003	−3.500373	1.799833	1	0	3.403078	2.997240	−0.265618
1	0	−5.285743	−2.052548	0.402571	1	0	5.512628	−0.017741	−1.301056
1	0	−4.498245	−1.236959	−0.956370	1	0	4.127089	−0.978208	−1.838716
1	0	−4.240574	−0.652611	0.704633	1	0	4.362027	−0.644621	−0.106487
1	0	−3.583958	1.797311	1.408244	1	0	3.330326	−1.997109	2.073821
1	0	−2.364627	4.470696	0.536070	1	0	1.253560	−4.121448	2.826153
3	0	1.062032	−1.918416	−0.827211	3	0	−0.619761	1.903643	−0.358644
8	0	2.822969	−2.722010	−0.746614	8	0	−1.765260	3.446041	−0.066394
6	0	3.863200	−1.783630	−1.124145	6	0	−3.030884	3.044634	0.522205
6	0	3.317968	−3.484691	0.364546	6	0	−1.314836	4.688448	0.523666
6	0	4.682151	−1.507832	0.159633	6	0	−3.051215	3.732082	1.883690
1	0	3.348323	−0.906955	−1.521495	1	0	−3.020133	1.952637	0.555200
1	0	4.477250	−2.243054	−1.908830	1	0	−3.848739	3.397023	−0.121390
6	0	4.068772	−2.460185	1.216708	6	0	−2.371253	5.076025	1.571191
1	0	3.987418	−4.276047	−0.004009	1	0	−1.202832	5.435933	−0.268745
1	0	2.457549	−3.946471	0.855203	1	0	−0.330925	4.509439	0.973480
1	0	4.820345	−2.922427	1.863774	1	0	−3.096892	5.776486	1.141931
1	0	3.359558	−1.920613	1.853270	1	0	−1.926206	5.551764	2.450178
1	0	5.742393	−1.725125	−0.003900	1	0	−4.062905	3.845914	2.284681
1	0	4.597951	−0.462770	0.468443	1	0	−2.458674	3.154678	2.603003
8	0	0.876565	−0.284986	−1.357040	8	0	−1.257960	0.389702	0.182608
6	0	0.603676	1.030194	−1.386526	6	0	−1.303439	−0.932155	0.437487
1	0	0.700705	1.471613	−2.404032	1	0	−1.539295	−1.163891	1.502532
6	0	1.525384	1.859553	−0.475382	6	0	−2.357662	−1.667419	−0.405431
6	0	2.003109	3.116802	−0.866005	6	0	−3.222768	−2.613827	0.154873
6	0	1.950960	1.346756	0.758597	6	0	−2.491399	−1.369426	−1.768376
1	0	1.711941	3.518225	−1.834861	1	0	−3.148140	−2.840659	1.216992
6	0	2.802336	2.078975	1.586875	6	0	−3.447716	−2.013792	−2.551812
1	0	1.604369	0.361288	1.057585	1	0	−1.847299	−0.608368	−2.200777
1	0	3.111175	1.667406	2.545449	1	0	−3.535379	−1.770388	−3.608351
6	0	3.261837	3.338152	1.188300	6	0	−4.300916	−2.963744	−1.981877

1	0	3.931168	3.906108	1.829566	1	0	−5.051682	−3.461425	−2.590726
6	0	2.861463	3.853381	−0.044509	6	0	−4.188081	−3.258983	−0.623202
1	0	3.223818	4.824605	−0.373370	1	0	−4.854938	−3.986137	−0.165473
**Int2-3**, G = -1544.514952 Hartree<br>0 imaginary frequencies					**Int2-4**, G = -1544.518465 Hartree<br>0 imaginary frequencies				
6	0	−4.540685	0.972831	−1.219631	6	0	−2.484133	−4.088236	−1.069722
6	0	−3.920453	1.183491	0.166540	6	0	−1.264622	−3.697136	−1.912813
6	0	−3.737011	0.062940	−2.154006	6	0	−2.973753	−3.012384	−0.093736
1	0	−5.546581	0.548937	−1.097129	1	0	−3.311207	−4.360403	−1.739562
6	0	−2.297546	0.515426	−2.448102	6	0	−1.919650	−2.473132	0.891751
1	0	−4.268573	−0.000562	−3.113813	1	0	−3.799468	−3.434436	0.495209
1	0	−3.728068	−0.952355	−1.745464	1	0	−3.407166	−2.176690	−0.654705
1	0	−1.932544	−0.077444	−3.297568	1	0	−2.453442	−1.919253	1.671428
6	0	−1.208121	0.382129	−1.355510	6	0	−0.828268	−1.515575	0.348016
1	0	−2.317899	1.556015	−2.807028	1	0	−1.440441	−3.318768	1.408174
1	0	−0.266327	0.594667	−1.867312	1	0	−0.357866	−1.044996	1.216558
6	0	−1.300811	1.380936	−0.230328	6	0	0.284750	−2.207947	−0.406893
6	0	−2.628700	2.021664	0.169941	6	0	0.039253	−3.542071	−1.103188
1	0	−4.645219	1.725313	0.788514	1	0	−1.091517	−4.484897	−2.657417
1	0	−2.771742	2.872906	−0.516308	1	0	0.062292	−4.328270	−0.331747
1	0	−2.469610	2.461331	1.159172	1	0	0.906520	−3.719181	−1.745408
7	0	−0.315285	1.853827	0.463942	7	0	1.503195	−1.805452	−0.531251
16	0	1.338244	1.501601	0.410239	16	0	2.248321	−0.372614	−0.037446
6	0	1.928137	3.277918	0.693537	6	0	3.802526	−1.194343	0.673761
8	0	1.957953	1.175452	−0.970038	8	0	1.693271	0.376629	1.201475
6	0	3.460669	3.183666	0.675110	6	0	4.664060	−0.025045	1.171817
6	0	1.424460	3.731102	2.067803	6	0	4.507709	−1.946755	−0.459809
6	0	1.413994	4.170353	−0.437037	6	0	3.408752	−2.119256	1.826670
1	0	3.884028	4.175444	0.873011	1	0	5.604775	−0.416730	1.576604
1	0	3.825923	2.837956	−0.295266	1	0	4.154534	0.536424	1.958972
1	0	3.831975	2.503452	1.451691	1	0	4.915332	0.664785	0.356569
1	0	1.841504	5.175236	−0.331504	1	0	4.314016	−2.515004	2.304087
1	0	0.324111	4.258292	−0.405284	1	0	2.814862	−2.965855	1.469488
1	0	1.709745	3.772382	−1.412436	1	0	2.832947	−1.575932	2.581957
1	0	1.828471	4.727570	2.285867	1	0	5.459943	−2.345768	−0.087867
1	0	1.759602	3.054084	2.862570	1	0	4.730765	−1.284198	−1.304477
1	0	0.334079	3.781256	2.095276	1	0	3.899626	−2.775808	−0.827076
1	0	−3.751790	0.221554	0.660613	1	0	−1.467706	−2.781034	−2.482143
1	0	−4.679952	1.954886	−1.698354	1	0	−2.235636	−5.001237	−0.506743
3	0	1.897726	−0.691985	−1.217874	3	0	0.417500	1.568597	0.455261
8	0	3.389053	−1.923251	−1.305184	8	0	0.546083	3.475511	0.190421
6	0	2.925532	−3.284116	−1.037729	6	0	−0.337075	3.880296	−0.902031
6	0	4.578047	−1.667299	−0.534401	6	0	1.813188	4.139742	0.040670
6	0	4.413979	−2.527251	0.715904	6	0	1.986277	4.285496	−1.468840
1	0	4.619212	−0.590700	−0.346328	1	0	2.568590	3.518337	0.529579
1	0	5.464468	−1.960436	−1.115389	1	0	1.779168	5.118286	0.541583
1	0	3.731540	−2.036309	1.418876	1	0	2.313036	3.331524	−1.897744
1	0	5.361852	−2.716263	1.229088	1	0	2.714326	5.055610	−1.741703
6	0	3.772469	−3.803475	0.140321	6	0	0.552163	4.620512	−1.922078
1	0	3.062902	−3.878557	−1.947322	1	0	−1.124181	4.519995	−0.489362
1	0	1.859858	−3.198368	−0.807237	1	0	−0.775927	2.956887	−1.289466
1	0	3.162899	−4.335216	0.876019	1	0	0.351846	4.296368	−2.946981
1	0	4.548479	−4.491898	−0.212838	1	0	0.380811	5.701680	−1.873790
8	0	0.389432	−1.340581	−0.620180	8	0	−0.391021	0.607931	−0.808902
6	0	−0.929380	−1.155917	−0.868928	6	0	−1.381172	−0.285155	−0.522121
6	0	−1.782543	−1.642681	0.313749	6	0	−2.540046	0.411597	0.215105
1	0	−1.283508	−1.729362	−1.755693	1	0	−1.819985	−0.723821	−1.442196
6	0	−1.467387	−1.264265	1.627887	6	0	−2.396348	0.888486	1.528244
6	0	−2.205229	−1.742542	2.710038	6	0	−3.409476	1.620904	2.147450
1	0	−0.627758	−0.598576	1.797512	1	0	−1.484618	0.673486	2.085393
1	0	−1.949305	−1.426463	3.718966	1	0	−3.276655	1.971017	3.168559
6	0	−3.261052	−2.636328	2.506392	6	0	−4.595544	1.897708	1.461295
1	0	−3.831839	−3.013856	3.351182	1	0	−5.385925	2.468938	1.941361
6	0	−3.560308	−3.053991	1.209998	6	0	−4.754523	1.431514	0.156197
1	0	−4.361054	−3.769455	1.037327	1	0	−5.671724	1.641562	−0.389234
6	0	−2.821609	−2.562903	0.129660	6	0	−3.735973	0.695331	−0.455151

1	0	−3.047184	−2.915005	−0.875241	1	0	−3.863154	0.343703	−1.477309

XI. References for supporting information

1. Dawood, R. S.; Georgiou, .I; Wilkie, R. P.; Lewis, W.; Stockman, R.A. *Chem. Eur. J.*, **2017**, *23*, 11153-11158.
2. Li, J.; Jiang, S.; Procopiou, G.; Stockman, R. A.; Yang, G. *Eur. J. Org. Chem*., **2016**, *2016,* 3500–3504.
3. Sirvent, J. A.; Foubelo, F; Yus, M. *Chem. Commun*., **2012**, *48*, 2543-2545.
4. Liu, G.; Cogan, D. A.; Owens, T. D.; Tang, T. P.; Ellman, J. A. *J. Org. Chem*., **1999**, *64*, 1278-1284.
5. (a) Stiles, M.; Winkler, R. R.; Chang, V.; Traynor, L. *J. Am. Chem. Soc.*, **1964**, *86*, 3337-3342; (b) House, H. O.; Crumrine, D. S.; Teranishi, A. Y.; Olmstead, H. D. *J. Am. Chem. Soc.*, **1973**, *95*, 3310-3324.
6. Gaussian 16, Revision C.01, M. J. Frisch, G. W. Trucks, H. B. Schlegel, G. E. Scuseria, M. A. Robb, J. R. Cheeseman, G. Scalmani, V. Barone, G. A. Petersson, H. Nakatsuji, X. Li, M. Caricato, A. V. Marenich, J. Bloino, B. G. Janesko, R. Gomperts, B. Mennucci, H. P. Hratchian, J. V. Ortiz, A. F. Izmaylov, J. L. Sonnenberg, D. Williams-Young, F. Ding, F. Lipparini, F. Egidi, J. Goings, B. Peng, A. Petrone, T. Henderson, D. Ranasinghe, V. G. Zakrzewski, J. Gao, N. Rega, G. Zheng, W. Liang, M. Hada, M. Ehara, K. Toyota, R. Fukuda, J. Hasegawa, M. Ishida, T. Nakajima, Y. Honda, O. Kitao, H. Nakai, T. Vreven, K. Throssell, J. A., Jr. Montgomery, J. E. Peralta, F. Ogliaro, M. J. Bearpark, J. J. Heyd, E. N. Brothers, K. N. Kudin, V. N. Staroverov, T. A. Keith, R. Kobayashi, J. Normand, K. Raghavachari, A. P. Rendell, J. C. Burant, S. S. Iyengar, J. Tomasi, M. Cossi, J. M. Millam, M. Klene, C. Adamo, R. Cammi, J. W. Ochterski, R. L. Martin, K. Morokuma, O. Farkas, J. B. Foresman and D. J. Fox, Gaussian, Inc., Wallingford CT, 2016.
7. Spartan'16. Wavefunction, Inc. Irvine, CA.
8. Pracht, P.; Bohle, F; Grimme, S. *Phys. Chem. Chem. Phys.*, **2020**, *22*, 7169.
9. (a) Head-Gordon, M.; People, J. A.; Frisch, M. J. *Chem. Phys. Lett*., **1988**, *153*, 503–506; (b) Becke, A. D. *J. Chem. Phys*., **1993**, *98*, 5648–5652; (c) Lee, C.; Yang, W.; Parr, R. G. *Phys. Rev. Condens. Matter Mater. Phys*., 1988, **37**, 785–789; (d) Vosko, S. H.; Wilk, L.; Nusair, M.; *Can. J. Phys*., **1980**, *58*, 1200–1211; (e) Stephens, P. J.; Devlin, F. .J.; Chabalowski , C. F.; Frisch, M. J.; *J. Phys. Chem*., **1994**, *98*, 11623–11627.
10. Grimme, S.; Antony, J.; Ehrlich, S.; Krieg, H.; *J. Chem. Phys*., **2010**, *132*, 154104.
11. Zhao, Y.; Truhlar, D. G.; *Theor. Chem. Acc.*, **2008**, *120*, 215.
12. R. S. Paton, J. Rodríguez-Guerra and J. I. Funes, GoodVibes: version 3.0.0; Zenodo, 2019; DOI: 10.5281/zenodo.3346166.
13. The PyMOL Molecular Graphics System, Version 2. Schrödinger, LLC.
14. GaussView, Version 6, Roy. Dennington, Todd A. Keith and John M. Millam, Semichem Inc., Shawnee Mission, KS, 2016.

XII. ^{1}H NMR and ^{13}C NMR spectra of novel compounds

(*R*)-((1*R*,2*R*,3*R*)-2-(((*S*)-*Tert*-butylsulfinyl)amino)-3-((*S*)-hydroxy(phenyl)methyl)cycloheptyl)(phenyl)methyl benzoate, 2a

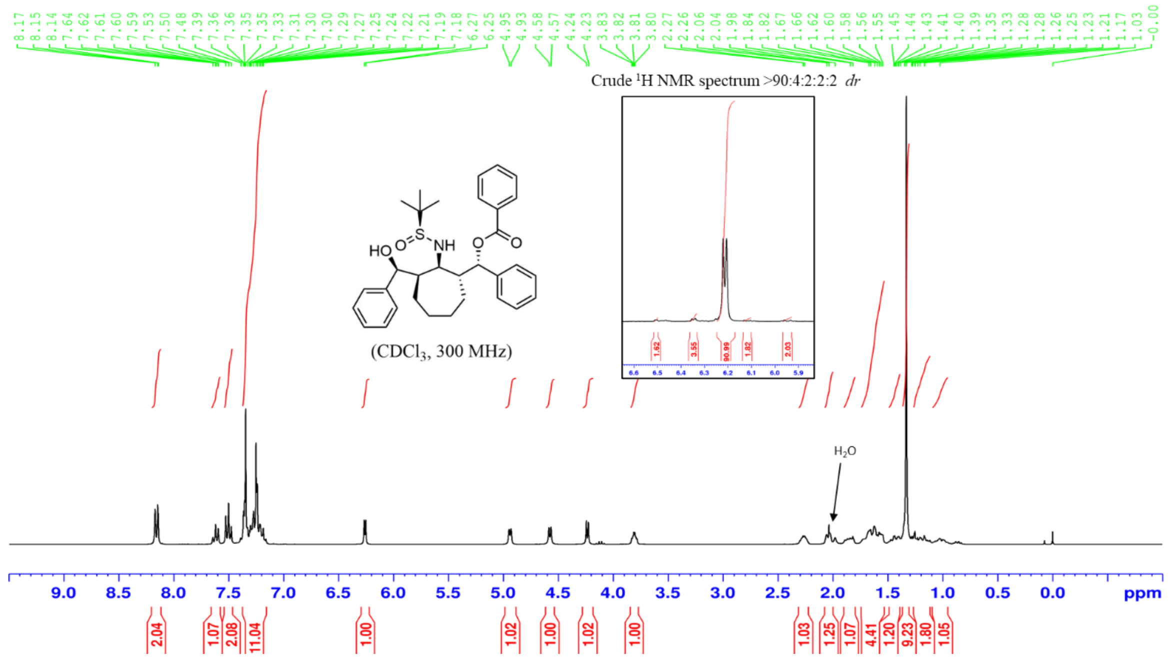

(*R*)-((1*R*,2*R*,3*R*)-2-(((*S*)-*Tert*-butylsulfinyl)amino)-3-((*S*)-hydroxy(phenyl)methyl)cycloheptyl)(phenyl)methyl benzoate, 2a

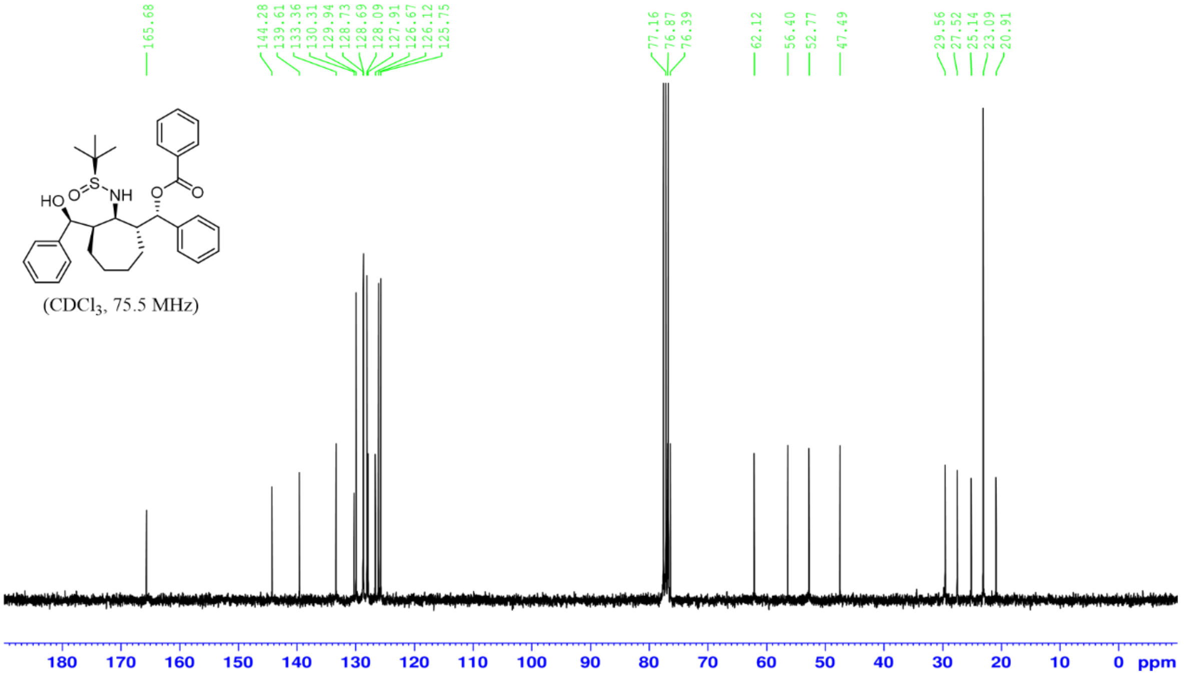

(*R*)-((1*R*,2*R*,3*R*)-2-(((*S*)-*Tert*-butylsulfinyl)amino)-3-((*S*)-hydroxy(*p*-tolyl)methyl)cycloheptyl)(*p*-tolyl)methyl 4-methylbenzoate, 2b

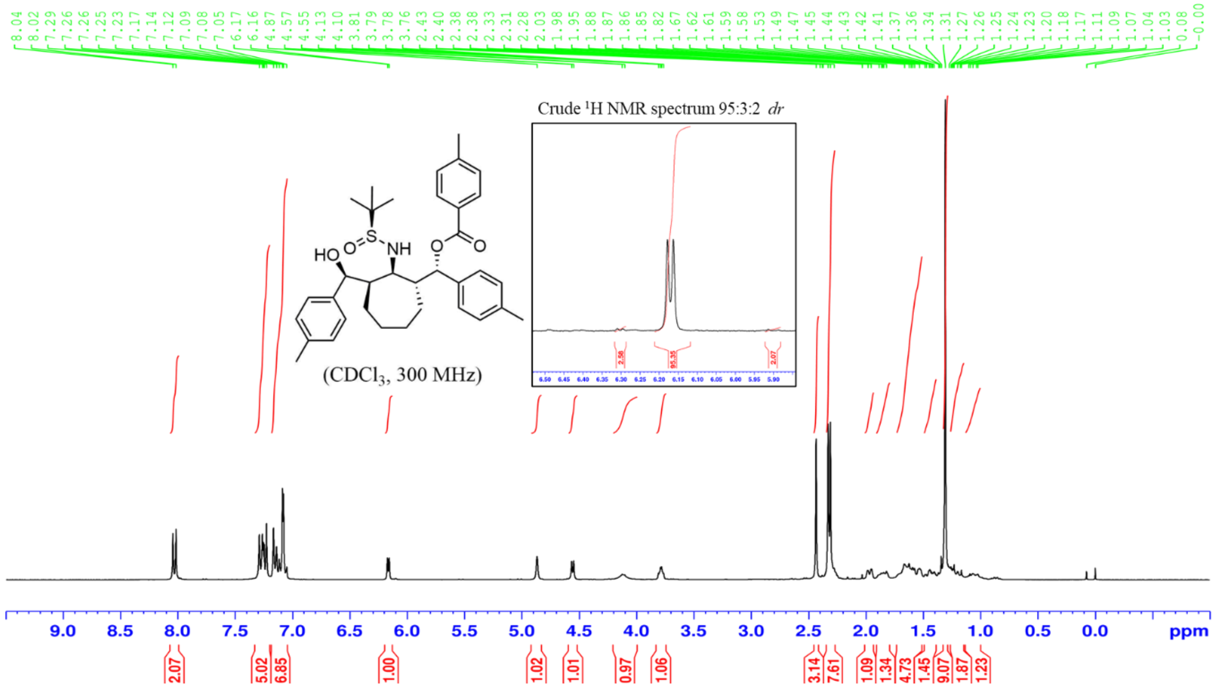

(*R*)-((1*R*,2*R*,3*R*)-2-(((*S*)-*Tert*-butylsulfinyl)amino)-3-((*S*)-hydroxy(*p*-tolyl)methyl)cycloheptyl)(*p*-tolyl)methyl 4-methylbenzoate, 2b

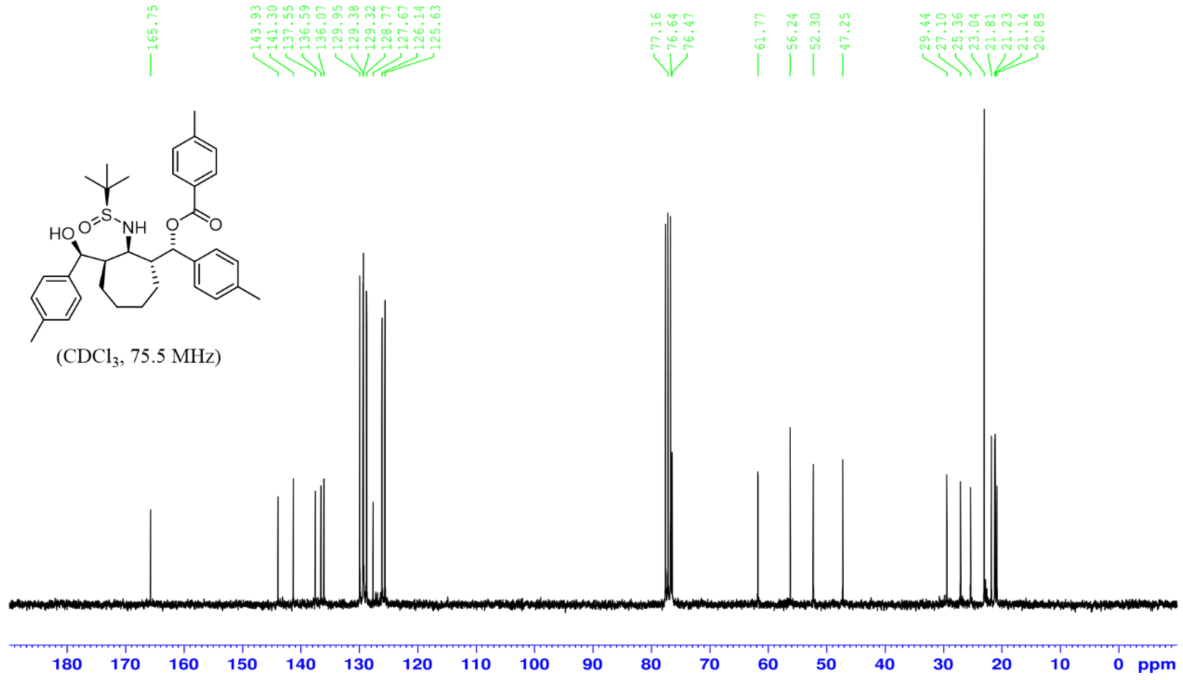

(*R*)-((1*R*,2*R*,3*R*)-2-(((*S*)-*Tert*-butylsulfinyl)amino)-3-((*S*)-hydroxy(m-tolyl)methyl)cycloheptyl)(m-tolyl)methyl 3-methylbenzoate, 2c

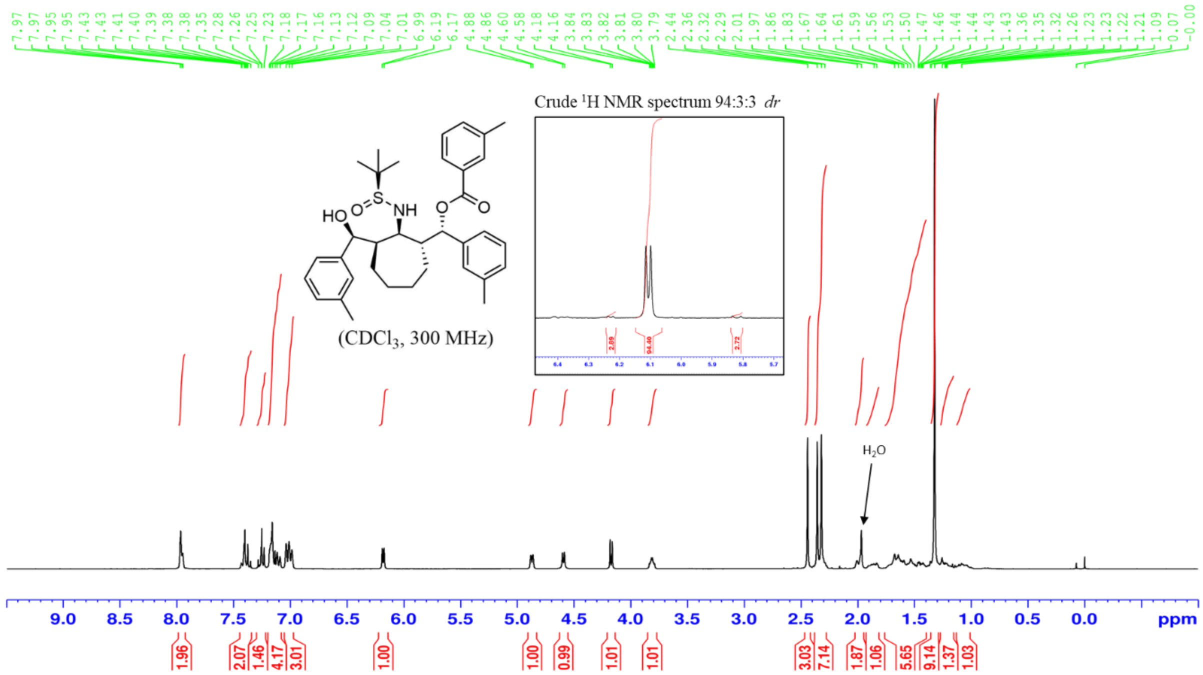

(*R*)-((1*R*,2*R*,3*R*)-2-(((*S*)-*Tert*-butylsulfinyl)amino)-3-((*S*)-hydroxy(m-tolyl)methyl)cycloheptyl)(m-tolyl)methyl 3-methylbenzoate, 2c

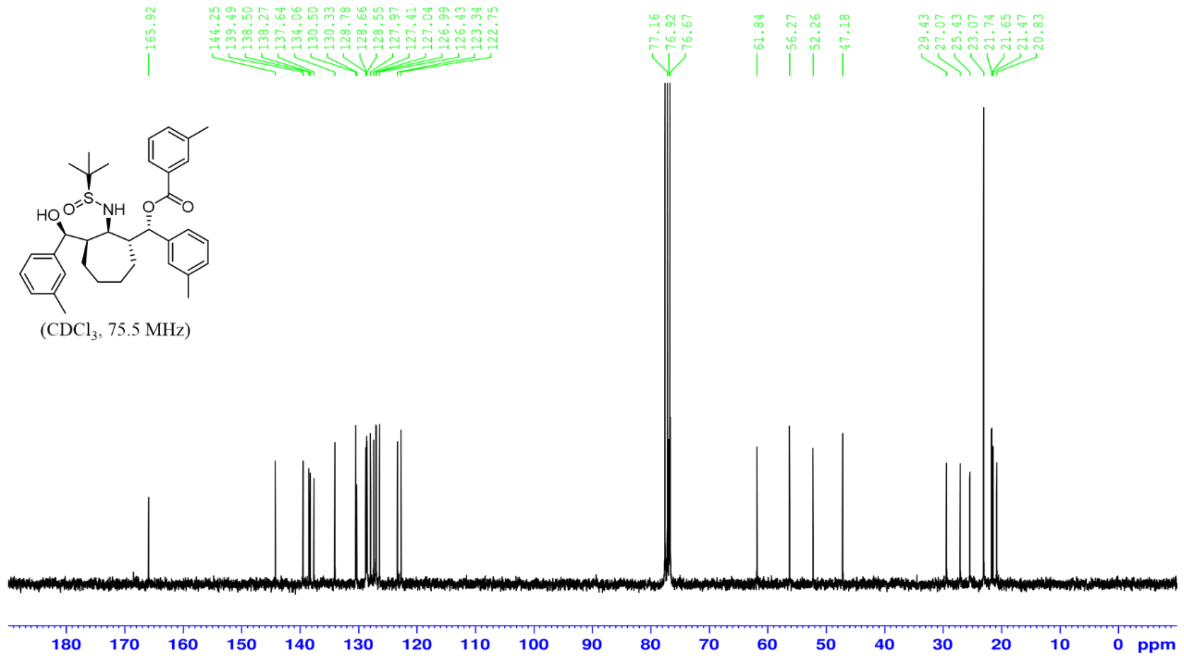

(*R*)-((1*R*,2*R*,3*R*)-2-(((*S*)-*Tert*-butylsulfinyl)amino)-3-((*S*)-hydroxy(4-isopropylphenyl)methyl)cycloheptyl)(4-isopropylphenyl)methyl 4-isopropylbenzoate, 2d

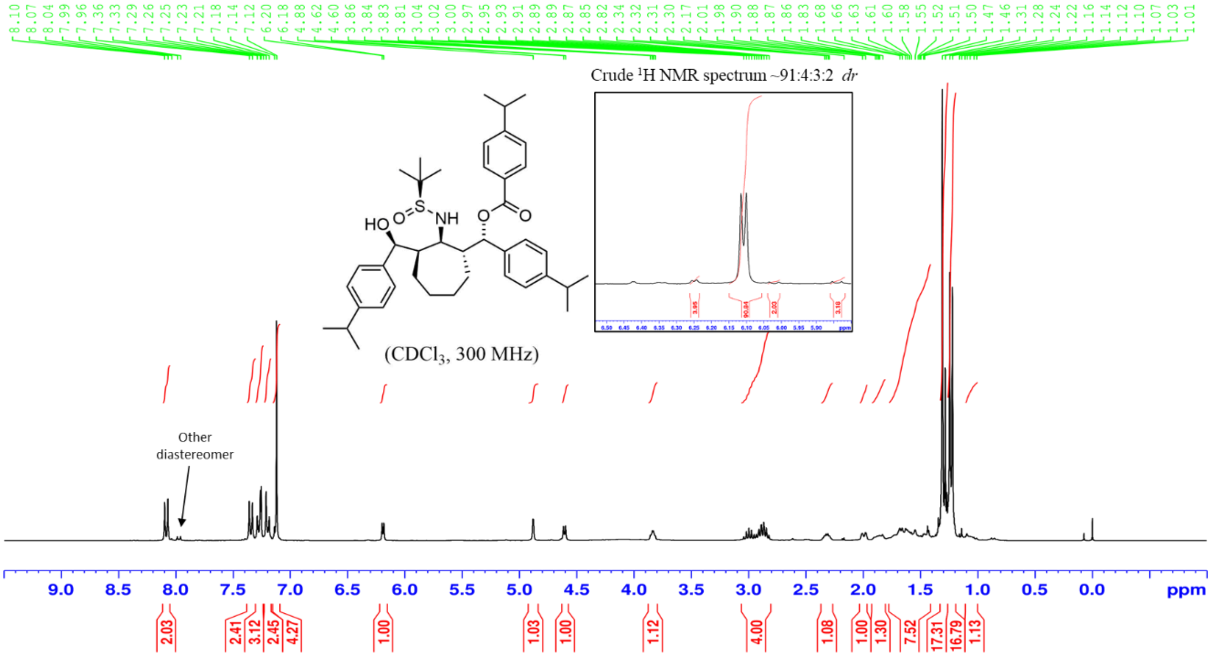

(*R*)-((1*R*,2*R*,3*R*)-2-(((*S*)-*Tert*-butylsulfinyl)amino)-3-((*S*)-hydroxy(4-isopropylphenyl)methyl)cycloheptyl)(4-isopropylphenyl)methyl 4-isopropylbenzoate, 2d

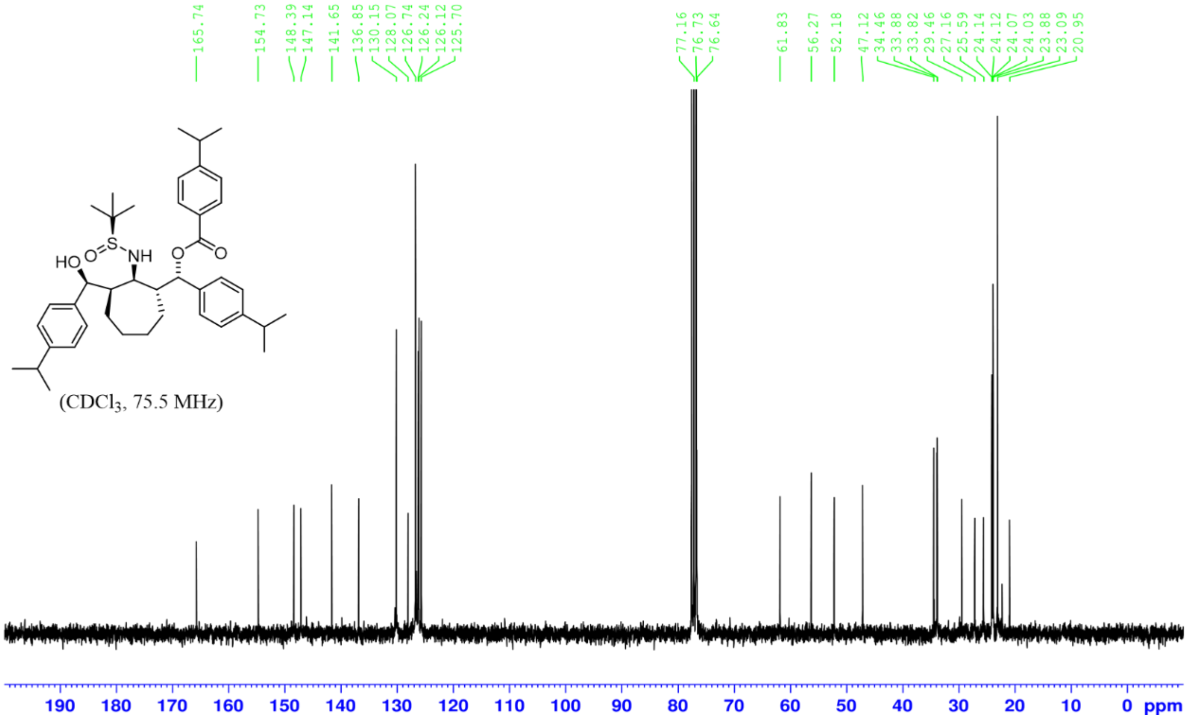

(*R*)-(4-(*Tert*-butyl)phenyl)((1*R*,2*R*,3*R*)-3-((*S*)-(4-(*tert*-butyl)phenyl)(hydroxy)methyl)-2-(((*S*)-*tert*-butylsulfinyl)amino)cycloheptyl)methyl 4-(*tert*-butyl)benzoate, 2e

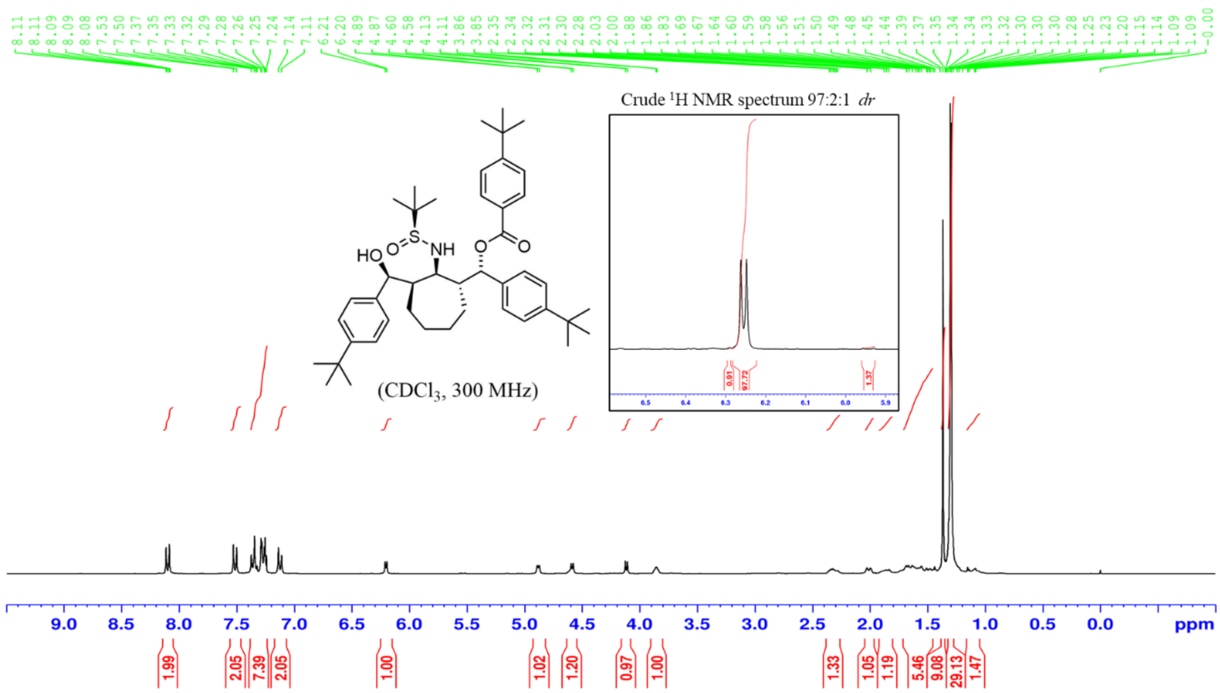

(*R*)-(4-(*Tert*-butyl)phenyl)((1*R*,2*R*,3*R*)-3-((*S*)-(4-(*tert*-butyl)phenyl)(hydroxy)methyl)-2-(((*S*)-*tert*-butylsulfinyl)amino)cycloheptyl)methyl 4-(*tert*-butyl)benzoate, 2e

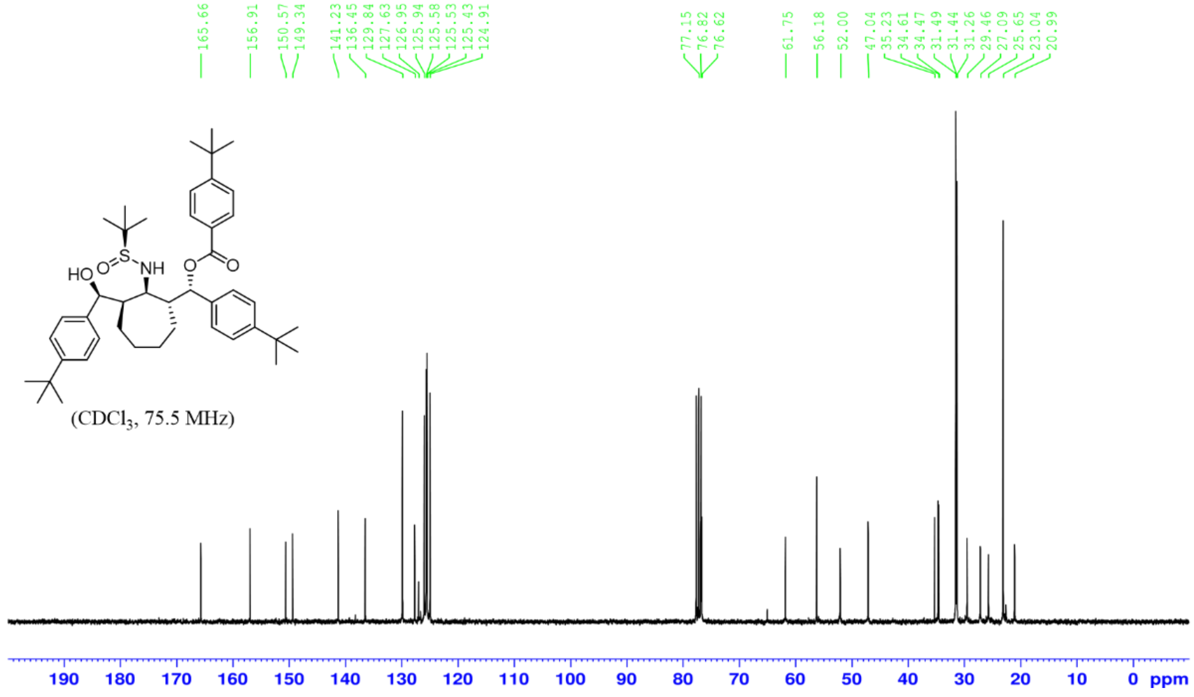

(*R*)-((1*R*,2*R*,3*R*)-2-(((*S*)-*Tert*-butylsulfinyl)amino)-3-((*S*)-hydroxy(4-(methylthio)phenyl)methyl)cycloheptyl)(4-(methylthio)phenyl)methyl 4-(methylthio)benzoate, 2f

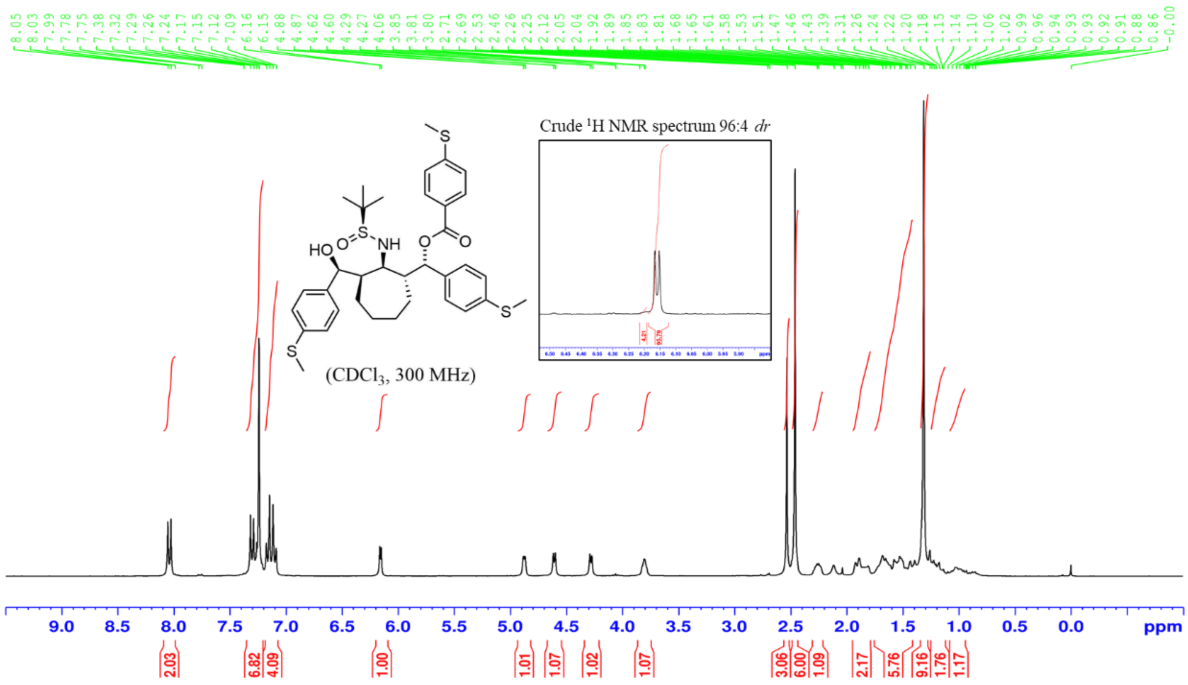

(*R*)-((1*R*,2*R*,3*R*)-2-(((*S*)-*Tert*-butylsulfinyl)amino)-3-((*S*)-hydroxy(4-(methylthio)phenyl)methyl)cycloheptyl)(4-(methylthio)phenyl)methyl 4-(methylthio)benzoate, 2f

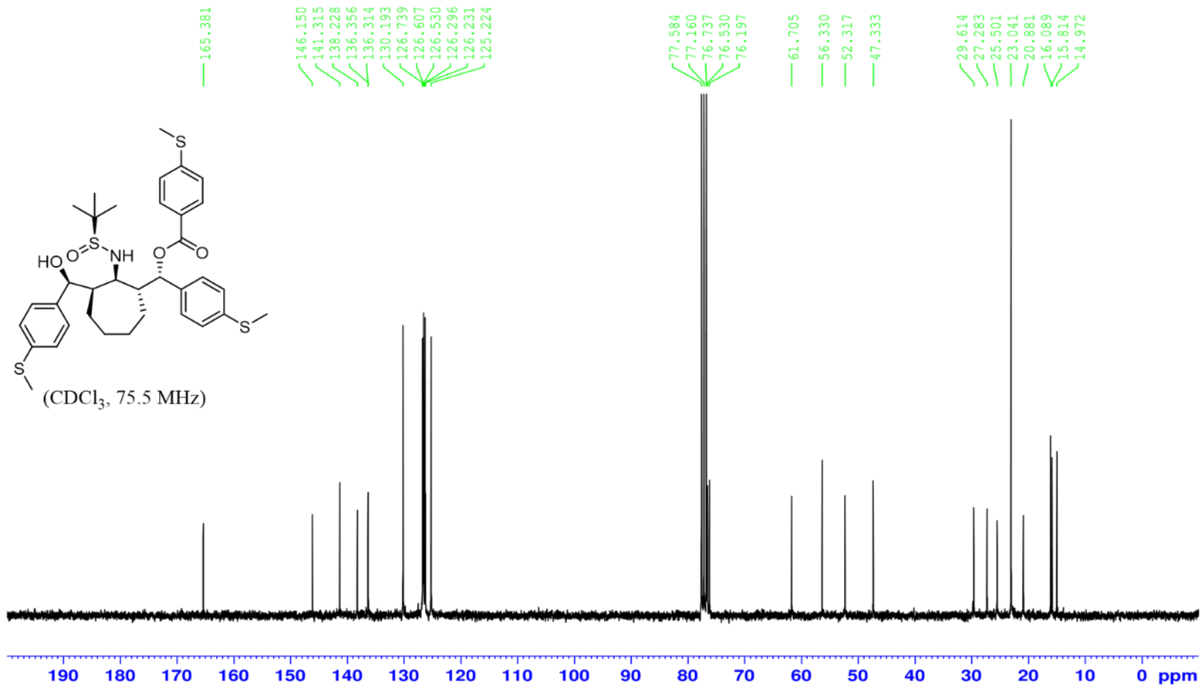

(*S*)-((1*R*,2*R*,3*R*)-2-(((*S*)-*Tert*-butylsulfinyl)amino)-3-((*S*)-hydroxy(4-methoxyphenyl)methyl)cycloheptyl)(4-methoxyphenyl)methyl 4-methoxybenzoate, 2g

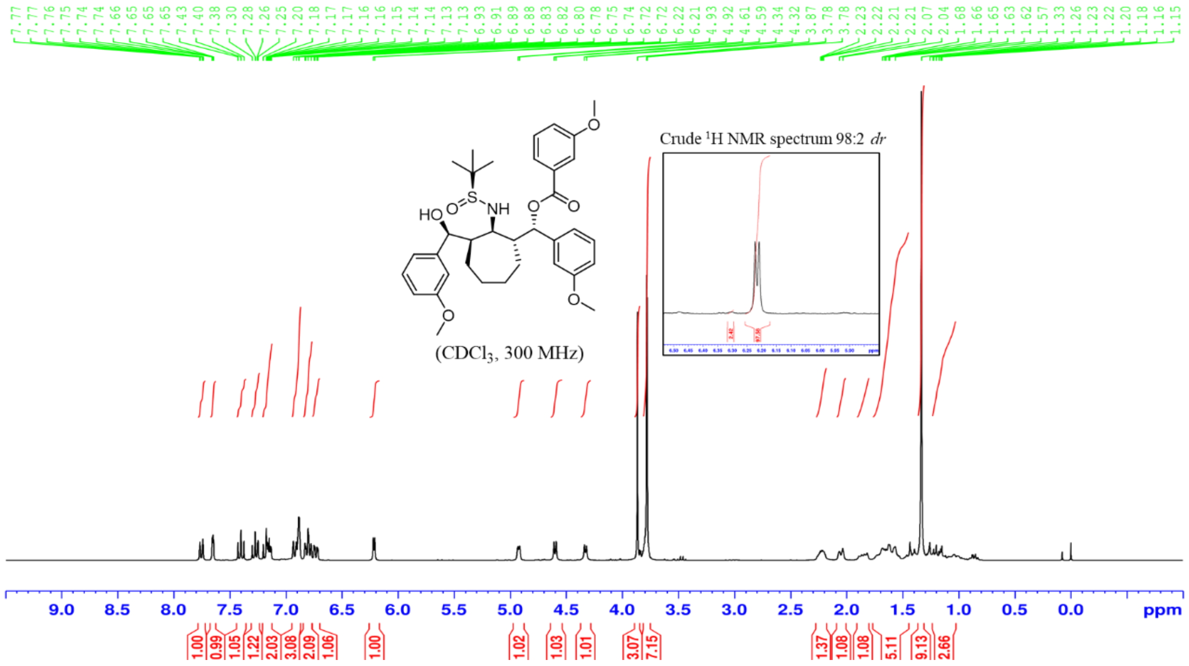

(*S*)-((1*R*,2*R*,3*R*)-2-(((*S*)-*Tert*-butylsulfinyl)amino)-3-((*S*)-hydroxy(4-methoxyphenyl)methyl)cycloheptyl)(4-methoxyphenyl)methyl 4-methoxybenzoate, 2g

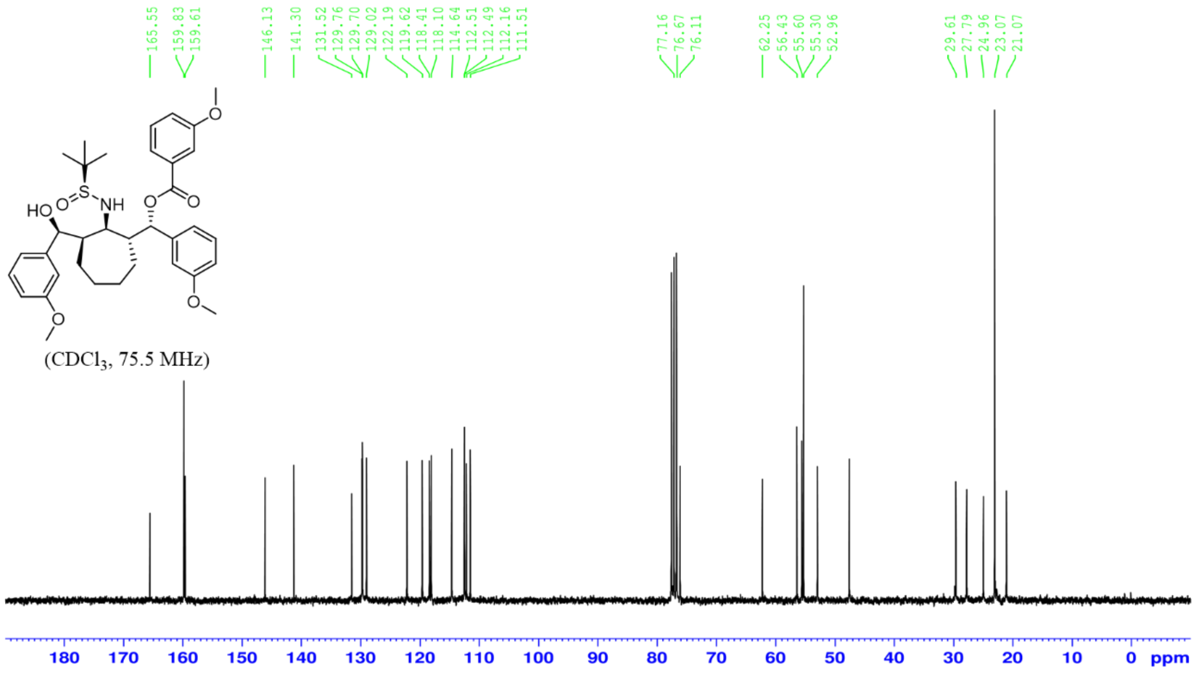

(*R*)-((1*R*,2*R*,3*R*)-2-(((*S*)-*Tert*-butylsulfinyl)amino)-3-((*S*)-(4-chlorophenyl)(hydroxy)methyl)cycloheptyl)(4-chlorophenyl)methyl 4-chlorobenzoate, 2h

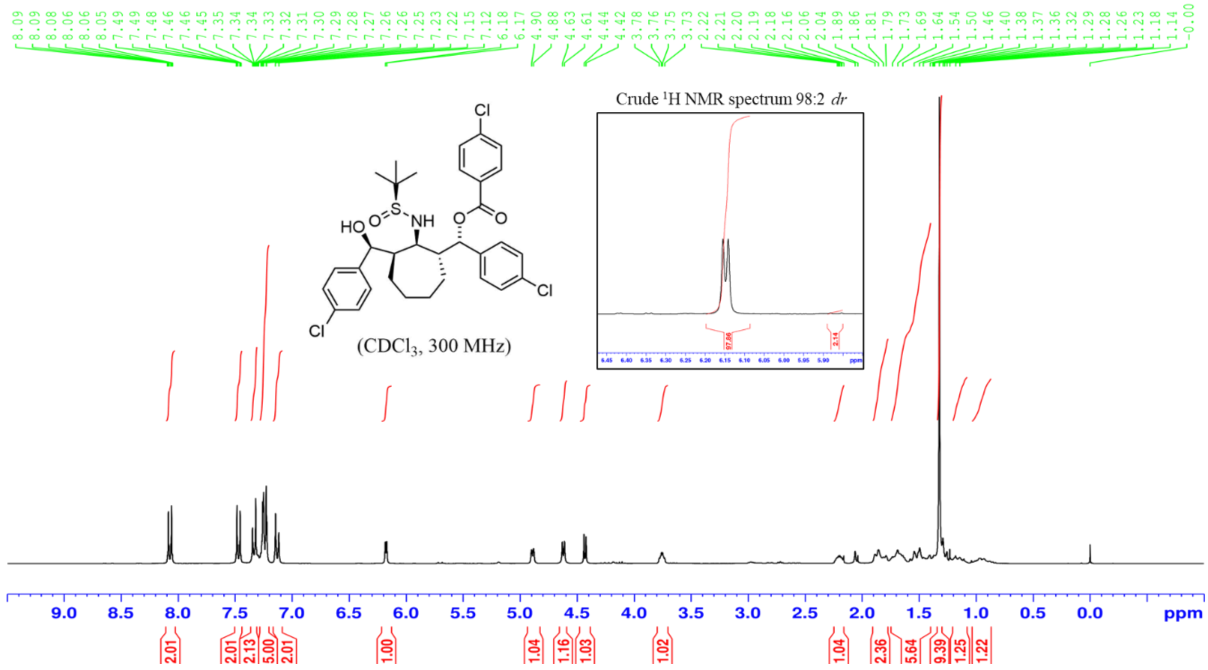

(*R*)-((1*R*,2*R*,3*R*)-2-(((*S*)-*Tert*-butylsulfinyl)amino)-3-((*S*)-(4-chlorophenyl)(hydroxy)methyl)cycloheptyl)(4-chlorophenyl)methyl 4-chlorobenzoate, 2h

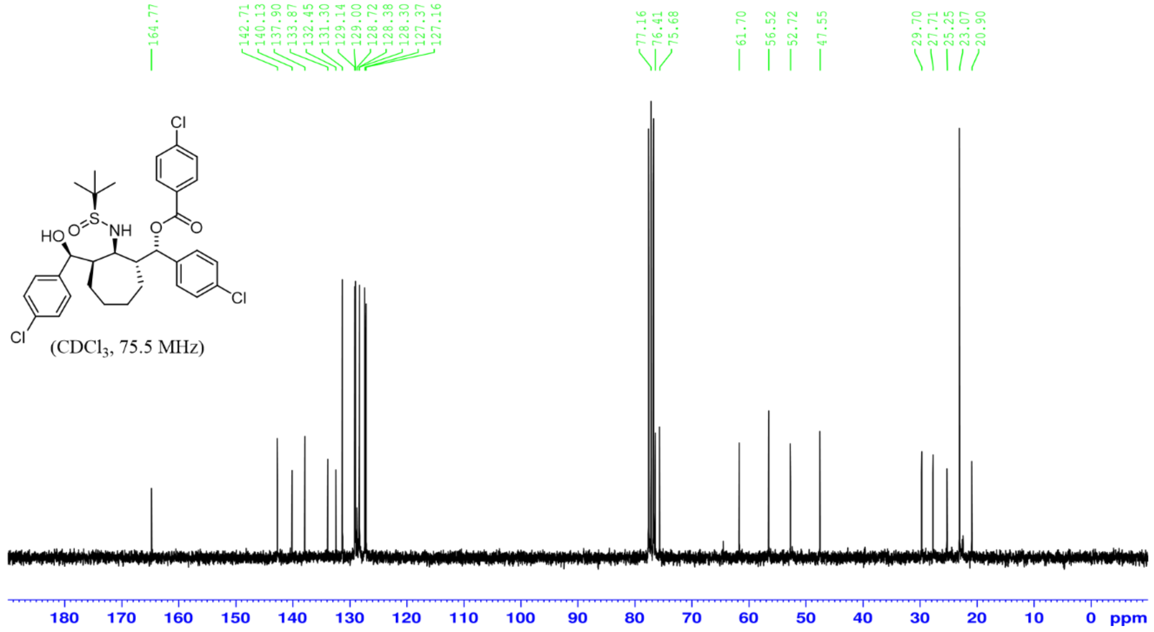

(*R*)-((1*R*,2*R*,3*R*)-2-(((*S*)-*Tert*-butylsulfinyl)amino)-3-((*S*)-(3-fluorophenyl)(hydroxy)methyl)cycloheptyl)(3-fluorophenyl)methyl 3-fluorobenzoate, 2i

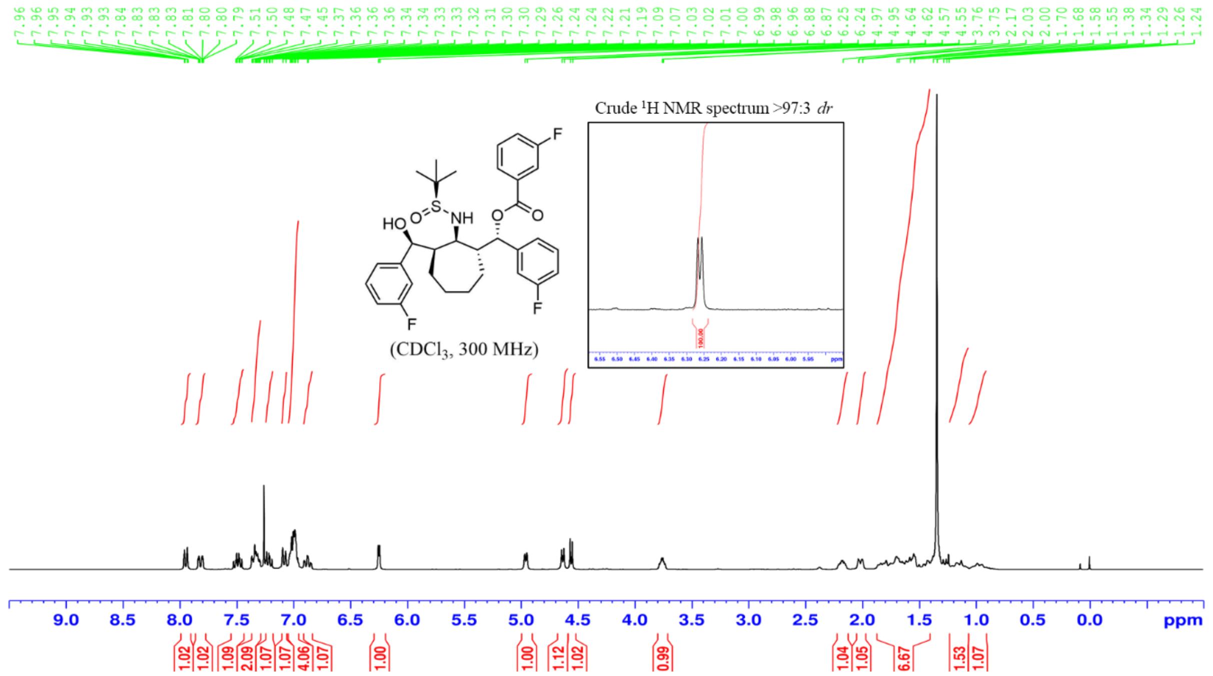

(*R*)-((1*R*,2*R*,3*R*)-2-(((*S*)-*Tert*-butylsulfinyl)amino)-3-((*S*)-(3-fluorophenyl)(hydroxy)methyl)cycloheptyl)(3-fluorophenyl)methyl 3-fluorobenzoate, 2i

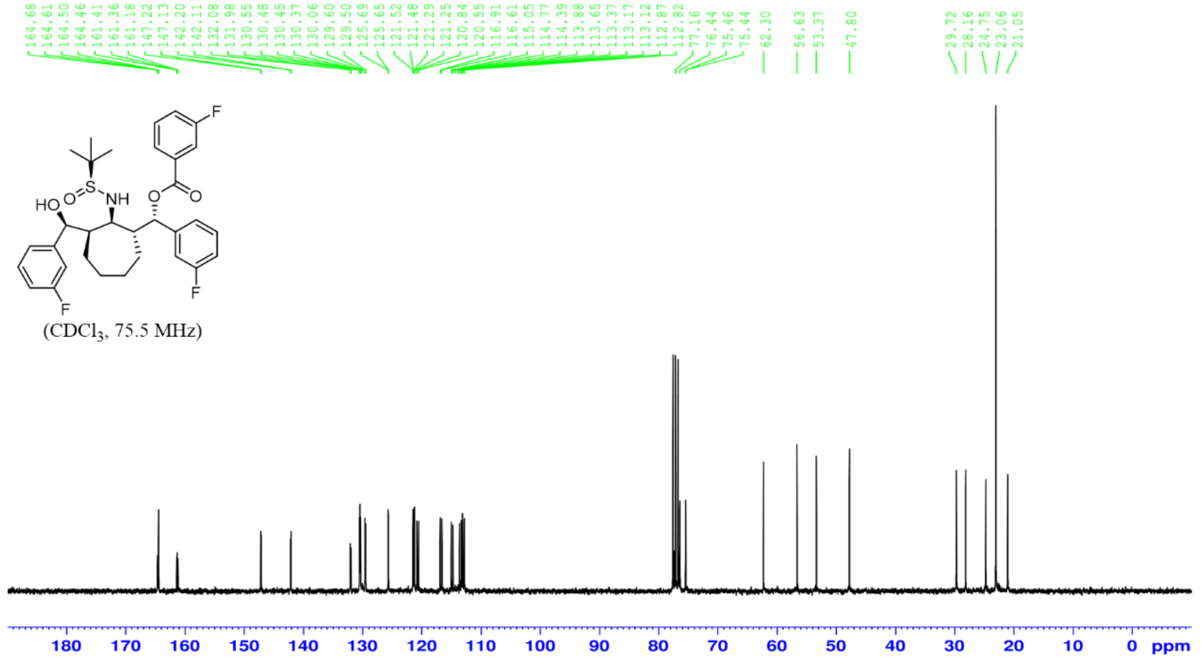

(*R*)-((1*R*,2*R*,3*R*)-2-(((*S*)-*Tert*-butylsulfinyl)amino)-3-((*S*)-hydroxy(4-(trifluoromethoxy)phenyl)methyl)cycloheptyl)(4-(trifluoromethoxy)phenyl)methyl 4-(trifluoromethoxy)benzoate, 2j

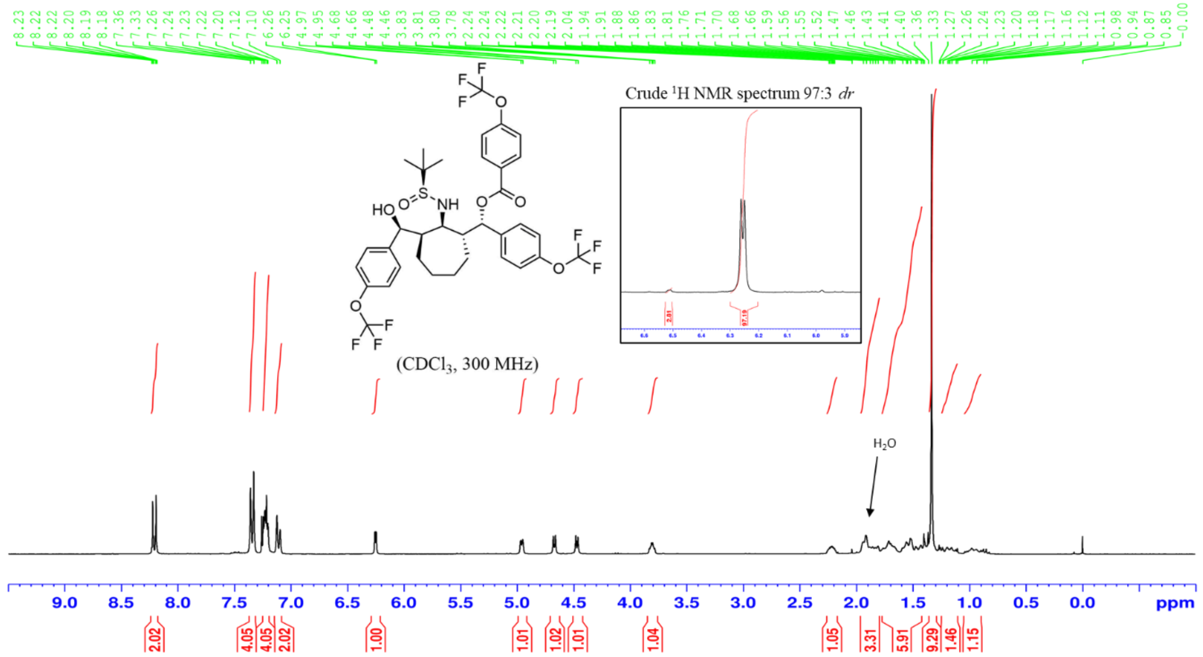

(*R*)-((1*R*,2*R*,3*R*)-2-(((*S*)-*Tert*-butylsulfinyl)amino)-3-((*S*)-hydroxy(4-(trifluoromethoxy)phenyl)methyl)cycloheptyl)(4-(trifluoromethoxy)phenyl)methyl 4-(trifluoromethoxy)benzoate, 2j

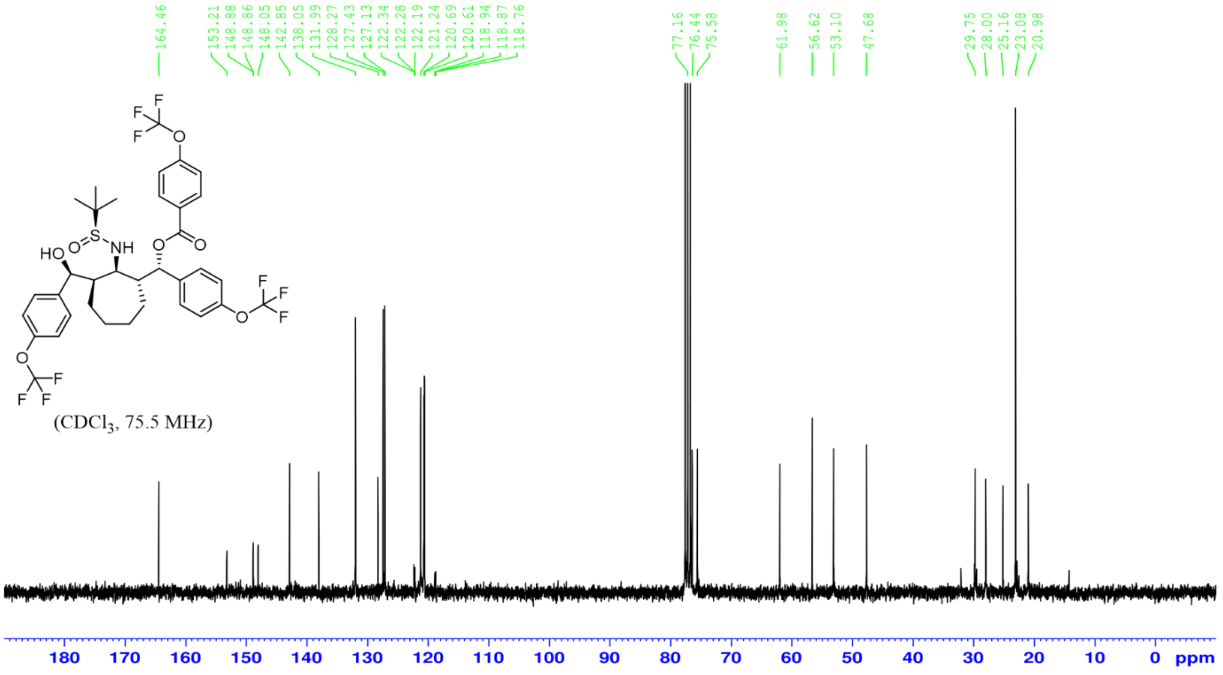

(*R*)-((1*R*,2*R*,3*R*)-2-(((*S*)-*Tert*-butylsulfinyl)amino)-3-((*S*)-(4-fluorophenyl)(hydroxy)methyl)cycloheptyl)(4-fluorophenyl)methyl 4-fluorobenzoate, 2k

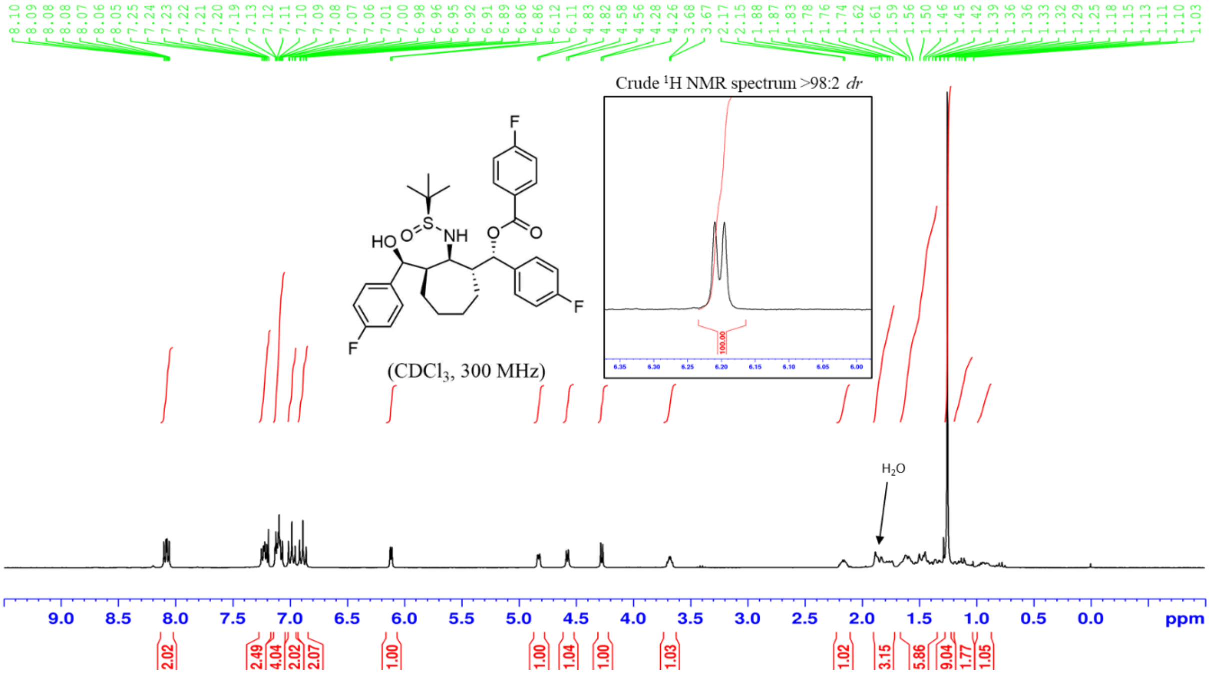

(*R*)-((1*R*,2*R*,3*R*)-2-(((*S*)-*Tert*-butylsulfinyl)amino)-3-((*S*)-(4-fluorophenyl)(hydroxy)methyl)cycloheptyl)(4-fluorophenyl)methyl 4-fluorobenzoate, 2k

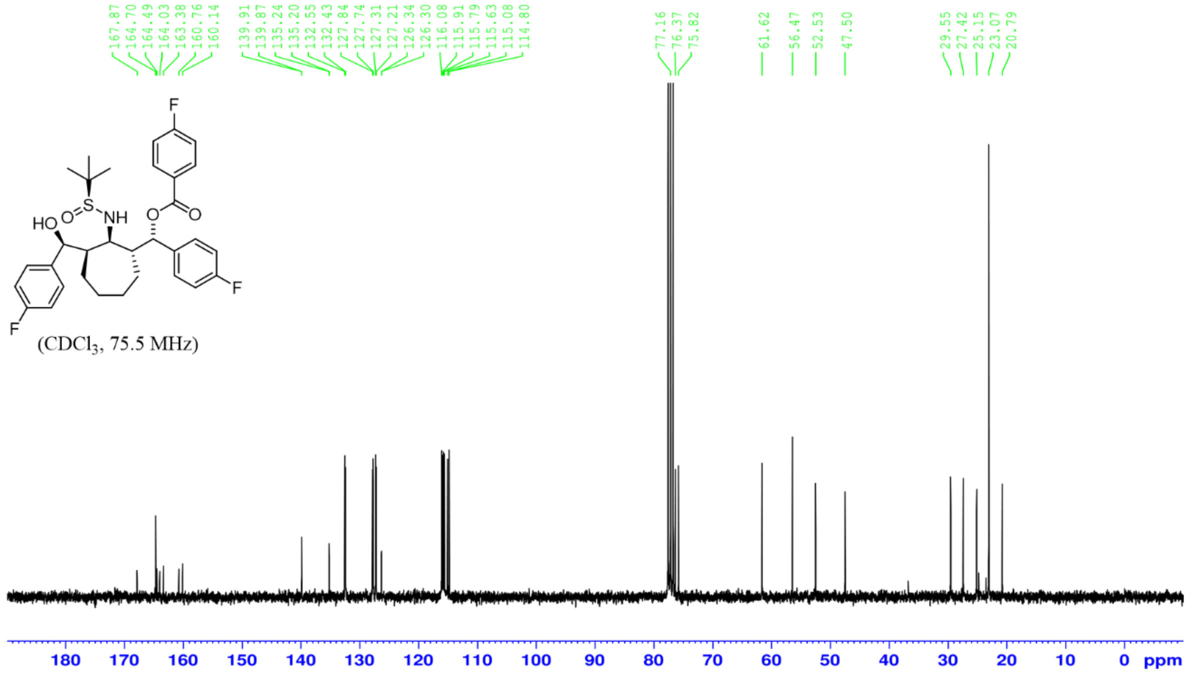

(*S*)-((1*R*,2*R*,3*R*)-2-(((*S*)-*Tert*-butylsulfinyl)amino)-3-((*S*)-hydroxy(4-(trifluoromethyl)phenyl)methyl)cycloheptyl)(4-(trifluoromethyl)phenyl)methyl 4-(trifluoromethyl)benzoate, 2l

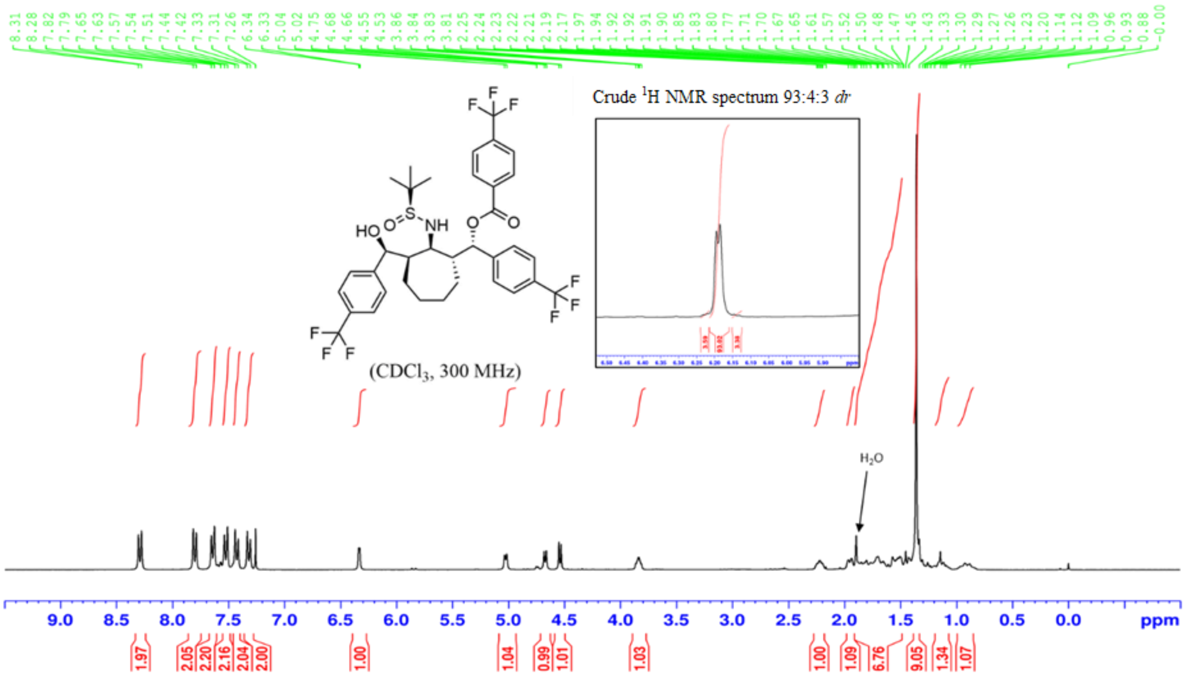

(*S*)-((1*R*,2*R*,3*R*)-2-(((*S*)-*Tert*-butylsulfinyl)amino)-3-((*S*)-hydroxy(4-(trifluoromethyl)phenyl)methyl)cycloheptyl)(4-(trifluoromethyl)phenyl)methyl 4-(trifluoromethyl)benzoate, 2l

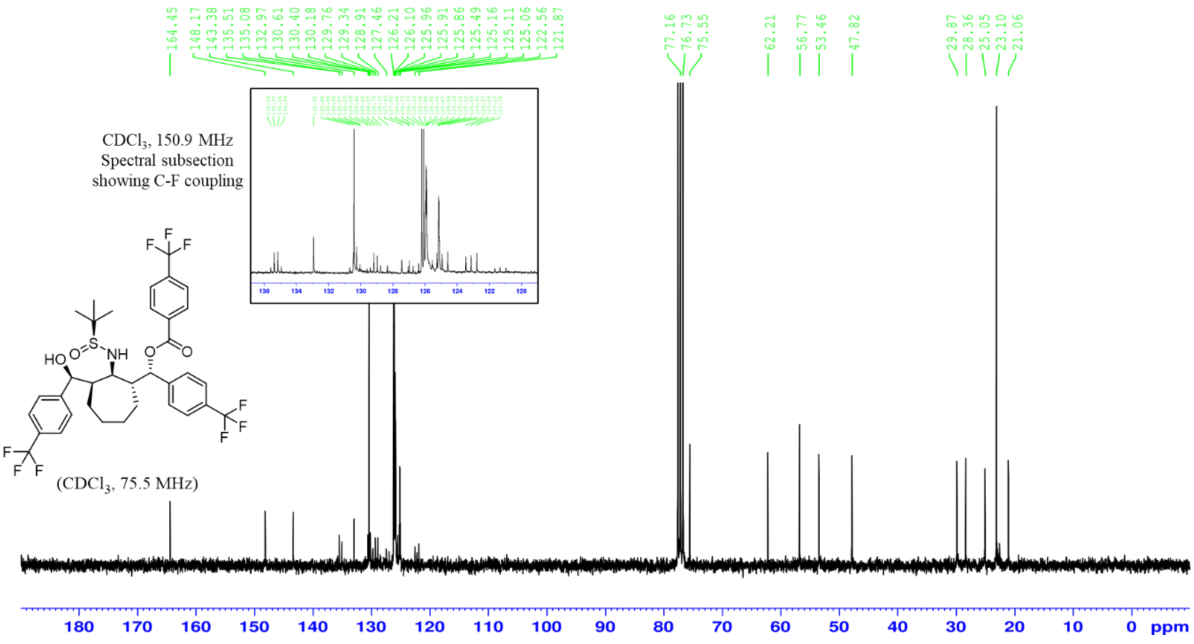

(*S*)-((1*R*,2*R*,3*R*)-2-(((*S*)-*Tert*-butylsulfinyl)amino)-3-((*S*)-hydroxy(4-(methoxycarbonyl)phenyl)methyl)cycloheptyl)(4-(methoxycarbonyl)phenyl)methyl methyl terephthalate, 2m

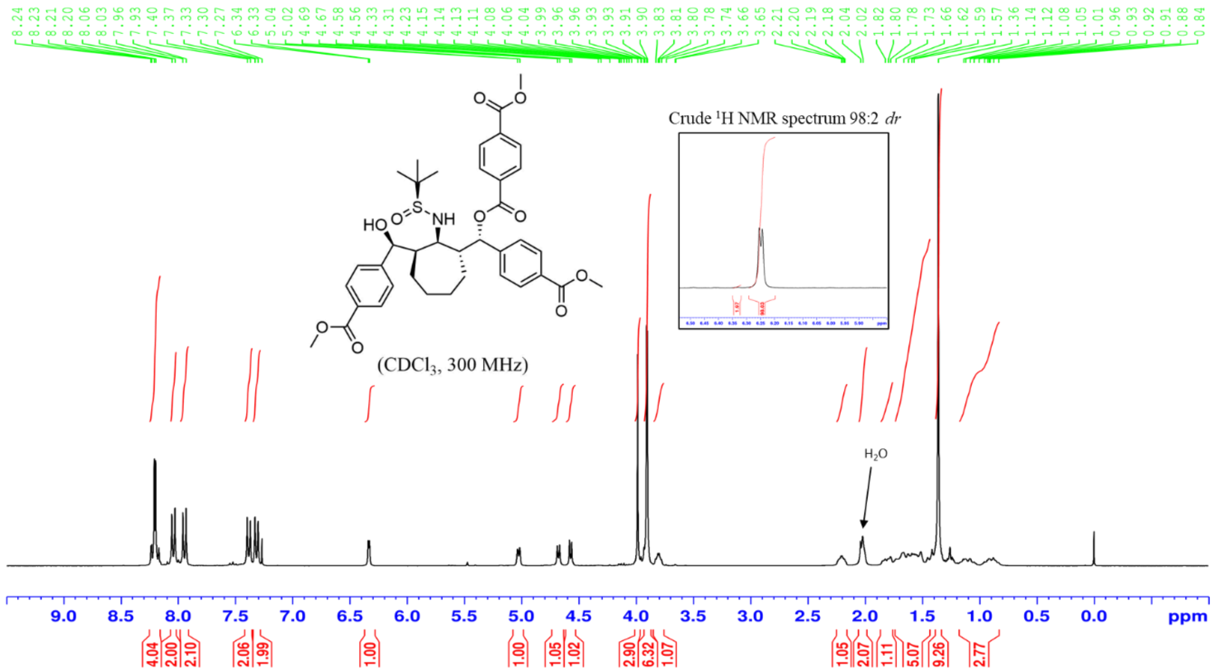

(*S*)-((1*R*,2*R*,3*R*)-2-(((*S*)-*Tert*-butylsulfinyl)amino)-3-((*S*)-hydroxy(4-(methoxycarbonyl)phenyl)methyl)cycloheptyl)(4-(methoxycarbonyl)phenyl)methyl methyl terephthalate, 2m

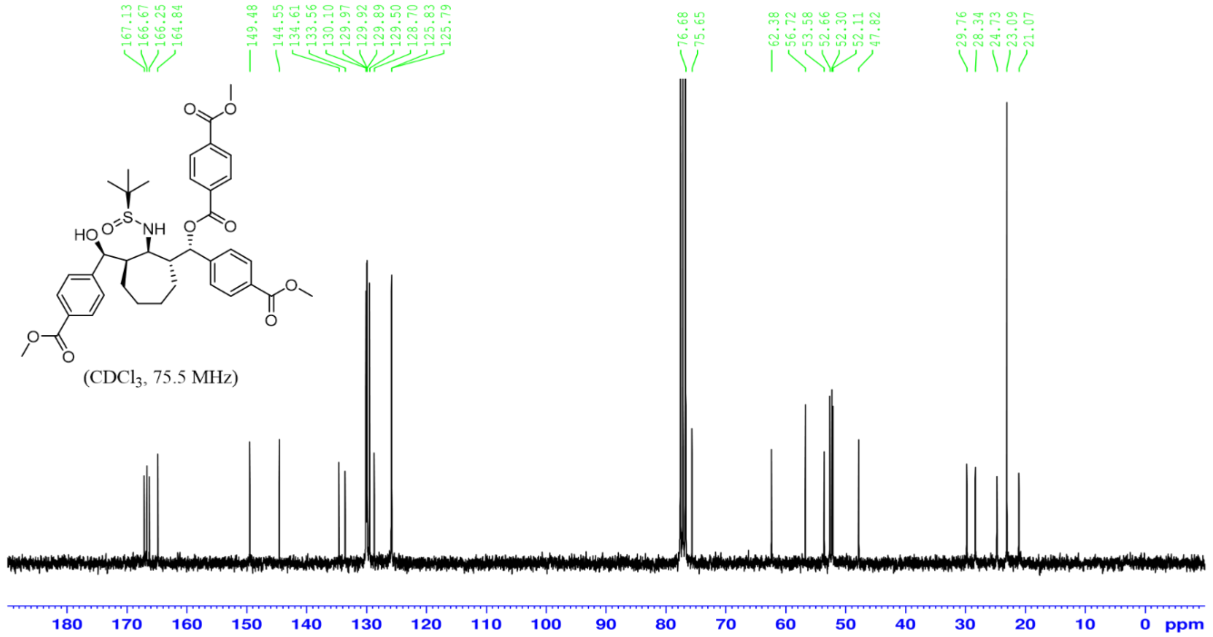

(*R*)-((1*R*,2*R*,3*R*)-2-(((*S*)-*Tert*-butylsulfinyl)amino)-3-((*S*)-hydroxy(3-(methoxycarbonyl)phenyl)methyl)cycloheptyl)(3-(methoxycarbonyl)phenyl)methyl methyl isophthalate, 2n

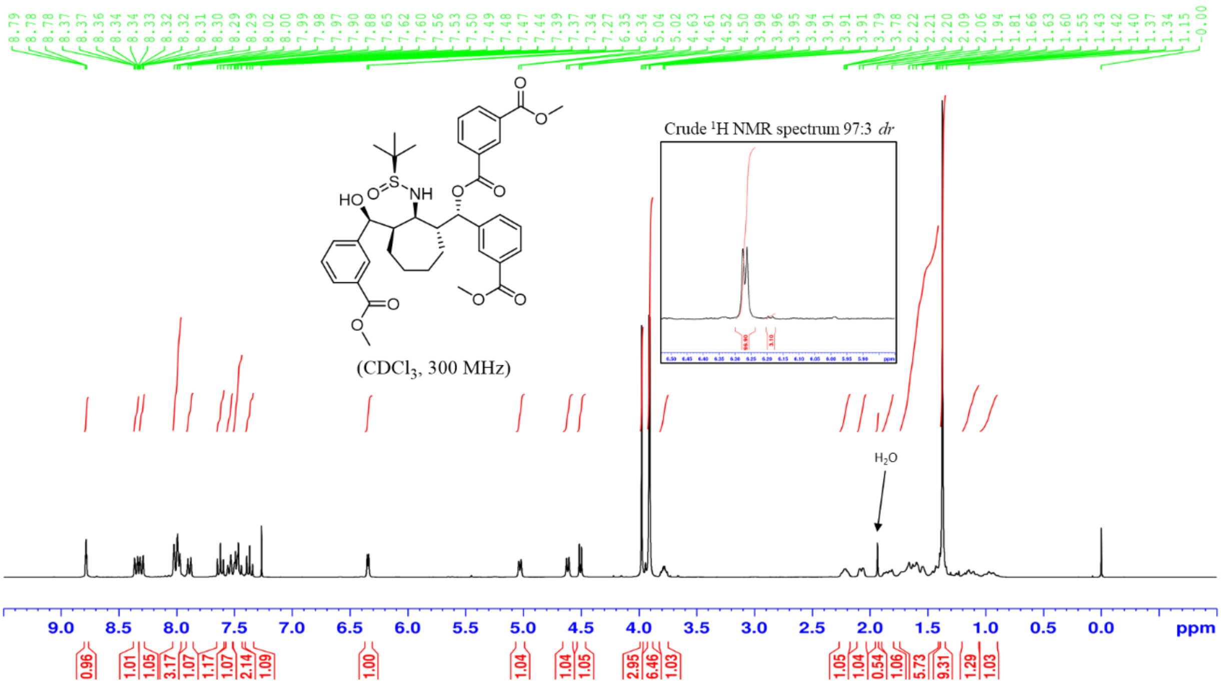

(*R*)-((1*R*,2*R*,3*R*)-2-(((*S*)-*Tert*-butylsulfinyl)amino)-3-((*S*)-hydroxy(3-(methoxycarbonyl)phenyl)methyl)cycloheptyl)(3-(methoxycarbonyl)phenyl)methyl methyl isophthalate, 2n

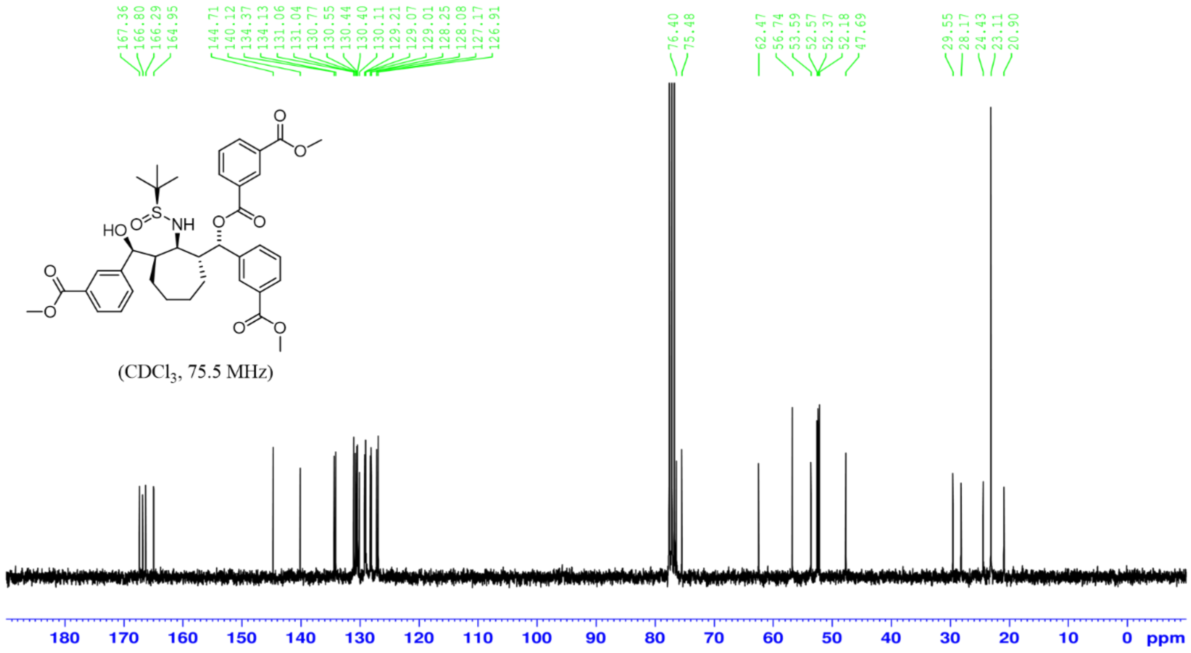

(*R*)-((1*R*,2*R*,3*R*)-2-(((*S*)-*Tert*-butylsulfinyl)amino)-3-((*S*)-(4-cyanophenyl)(hydroxy)methyl)cycloheptyl)(4-cyanophenyl)methyl 4-cyanobenzoate, 2o

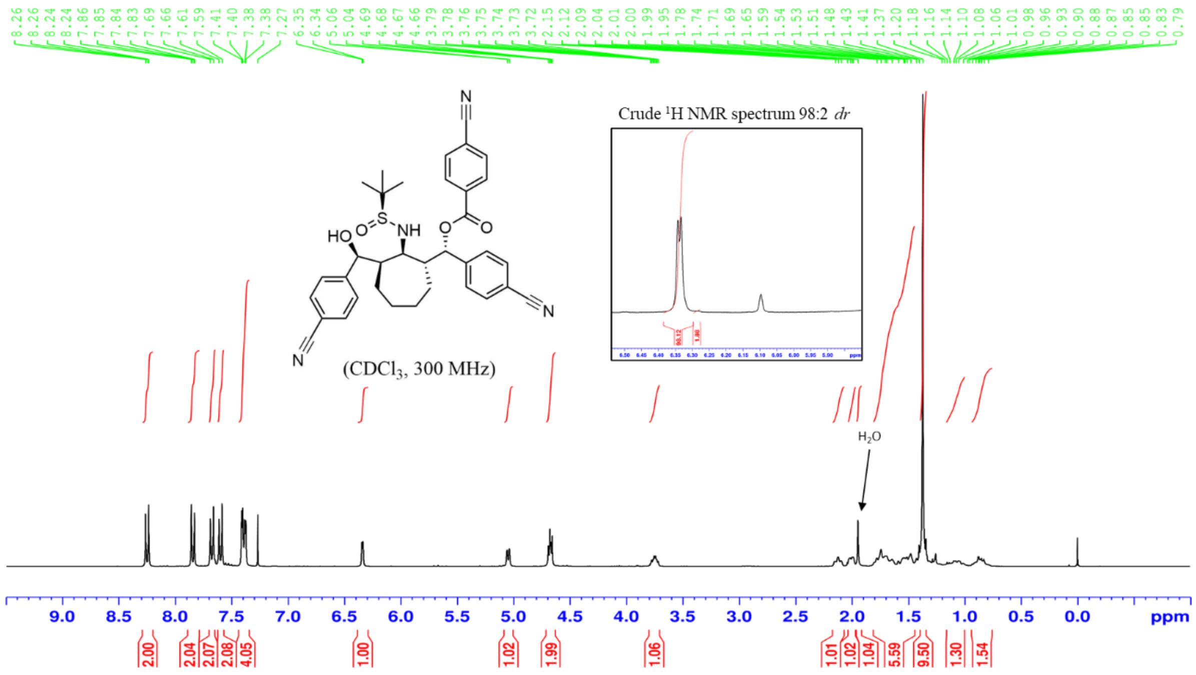

(*R*)-((1*R*,2*R*,3*R*)-2-(((*S*)-*Tert*-butylsulfinyl)amino)-3-((*S*)-(4-cyanophenyl)(hydroxy)methyl)cycloheptyl)(4-cyanophenyl)methyl 4-cyanobenzoate, 2o

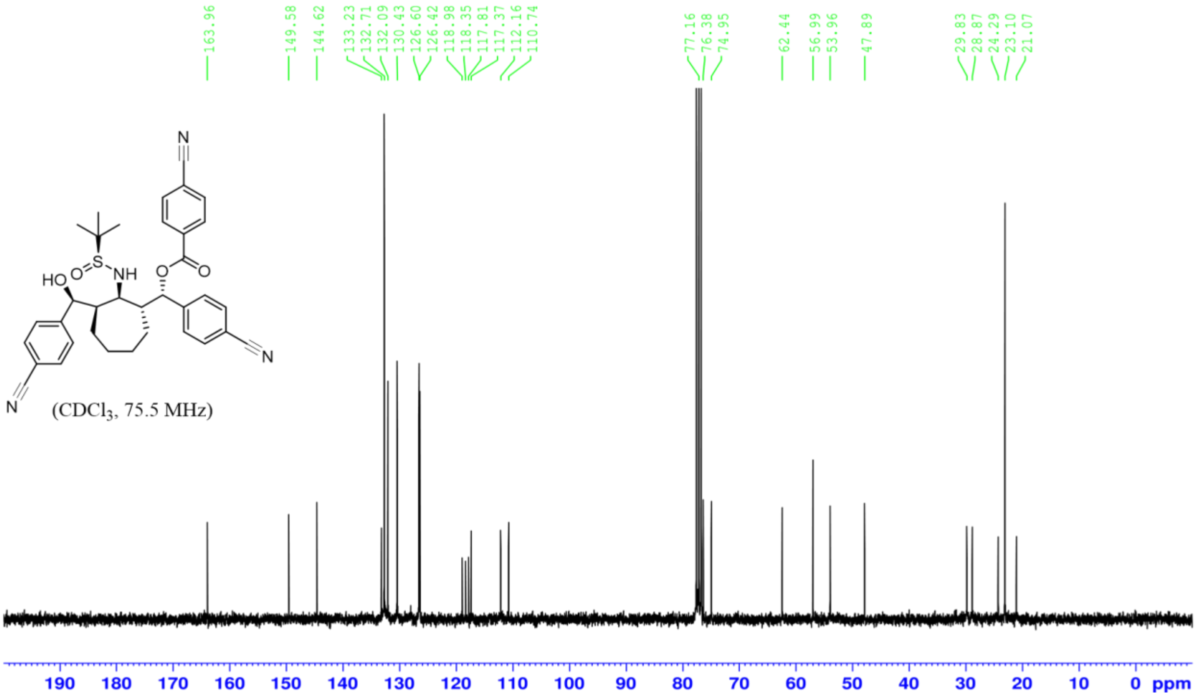

(*R*)-((1*R*,2*R*,3*R*)-2-(((*S*)-*Tert*-butylsulfinyl)amino)-3-((*S*)-hydroxy(pyridin-4-yl)methyl)cycloheptyl)(pyridin-4-yl)methyl isonicotinate, 2p

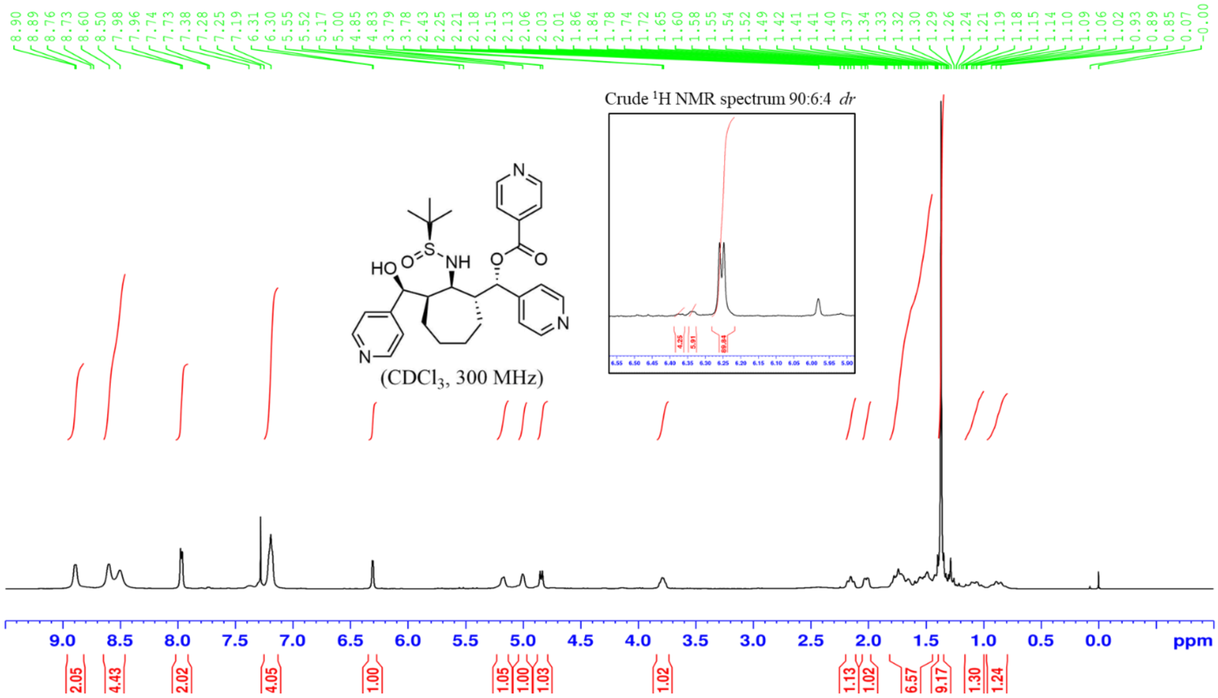

(*R*)-((1*R*,2*R*,3*R*)-2-(((*S*)-*Tert*-butylsulfinyl)amino)-3-((*S*)-hydroxy(pyridin-4-yl)methyl)cycloheptyl)(pyridin-4-yl)methyl isonicotinate, 2p

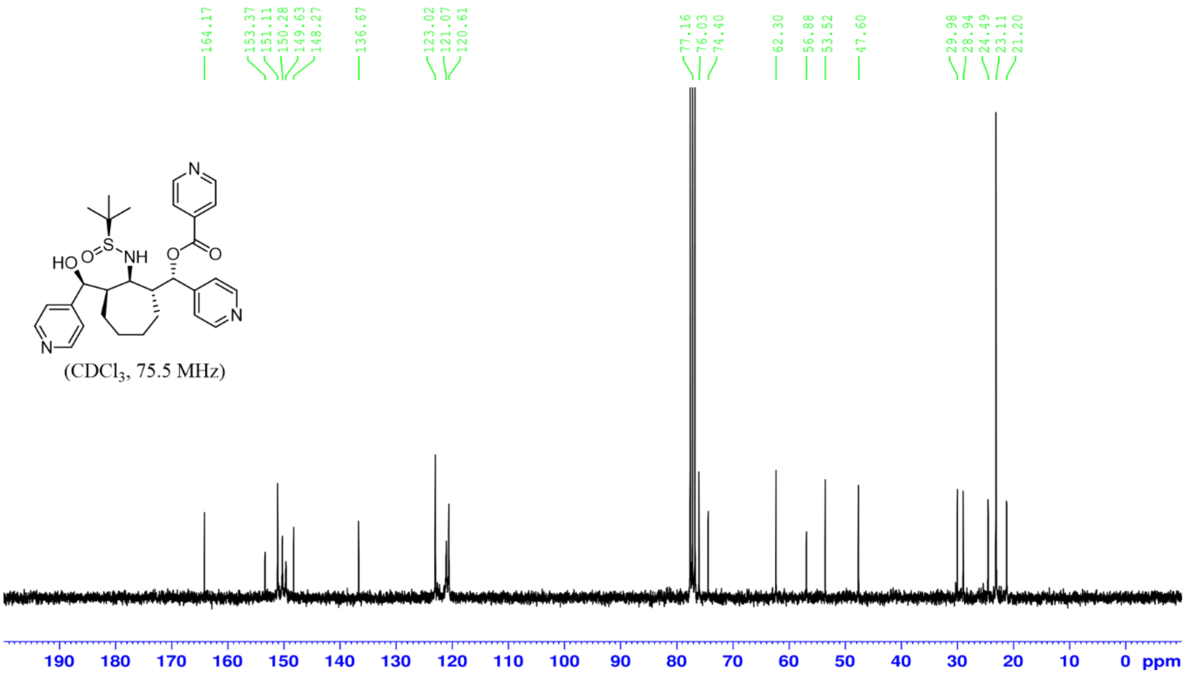

(*S*)-*N*-((2*R*,7*R*)-2,7-Bis((*S*)-furan-2-yl(hydroxy)methyl)cycloheptyl)-2-methylpropane-2-sulfinamide, 2s

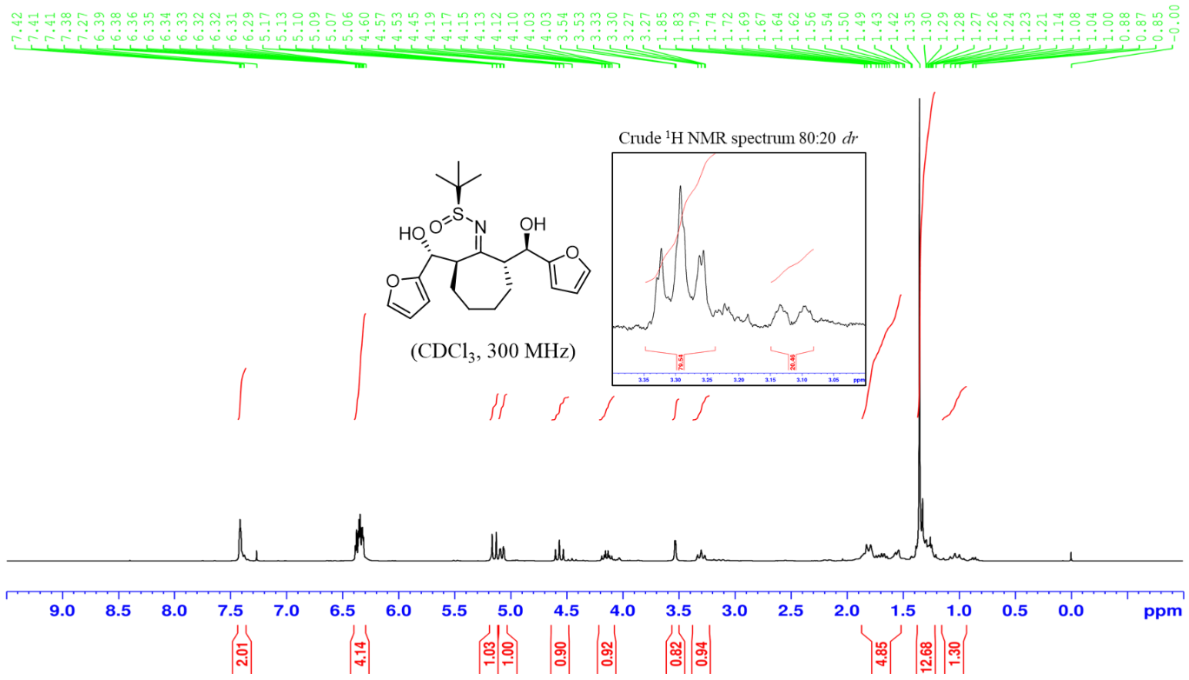

(*S*)-*N*-((2*R*,7*R*)-2,7-Bis((*S*)-furan-2-yl(hydroxy)methyl)cycloheptyl)-2-methylpropane-2-sulfinamide, 2s

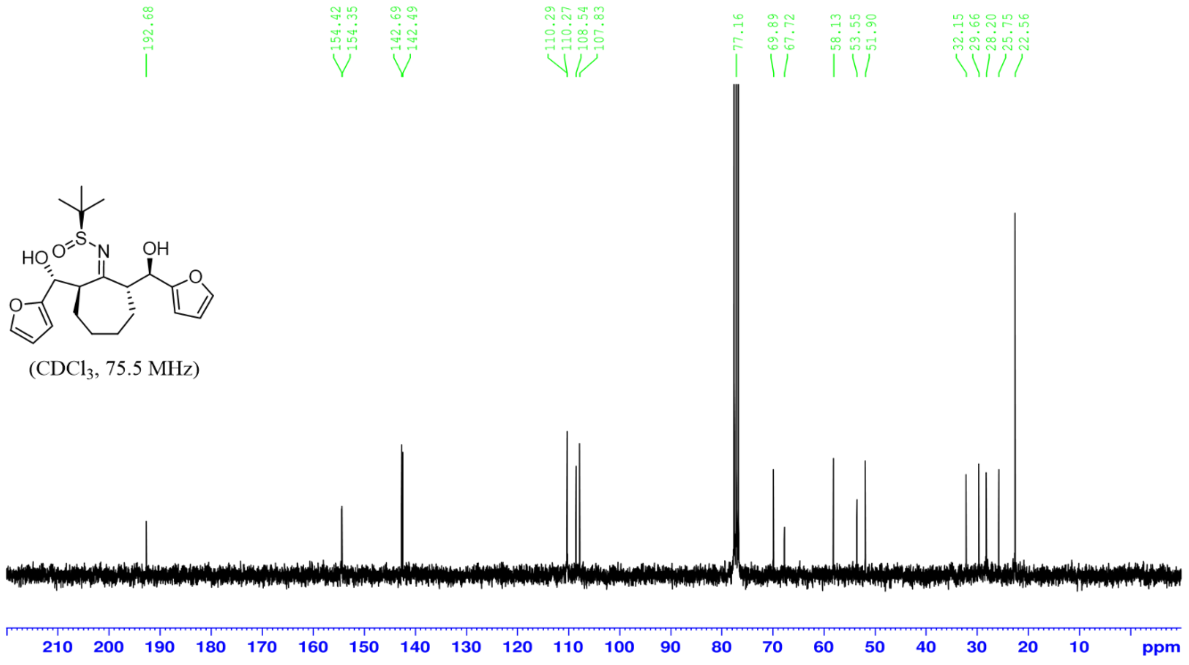

(*R*)-((1*R*,2*R*,3*R*)-2-(((*S*)-*Tert*-butylsulfinyl)amino)-3-((*S*)-hydroxy(phenyl)methyl)cyclopentyl)(phenyl)methyl benzoate, 3a

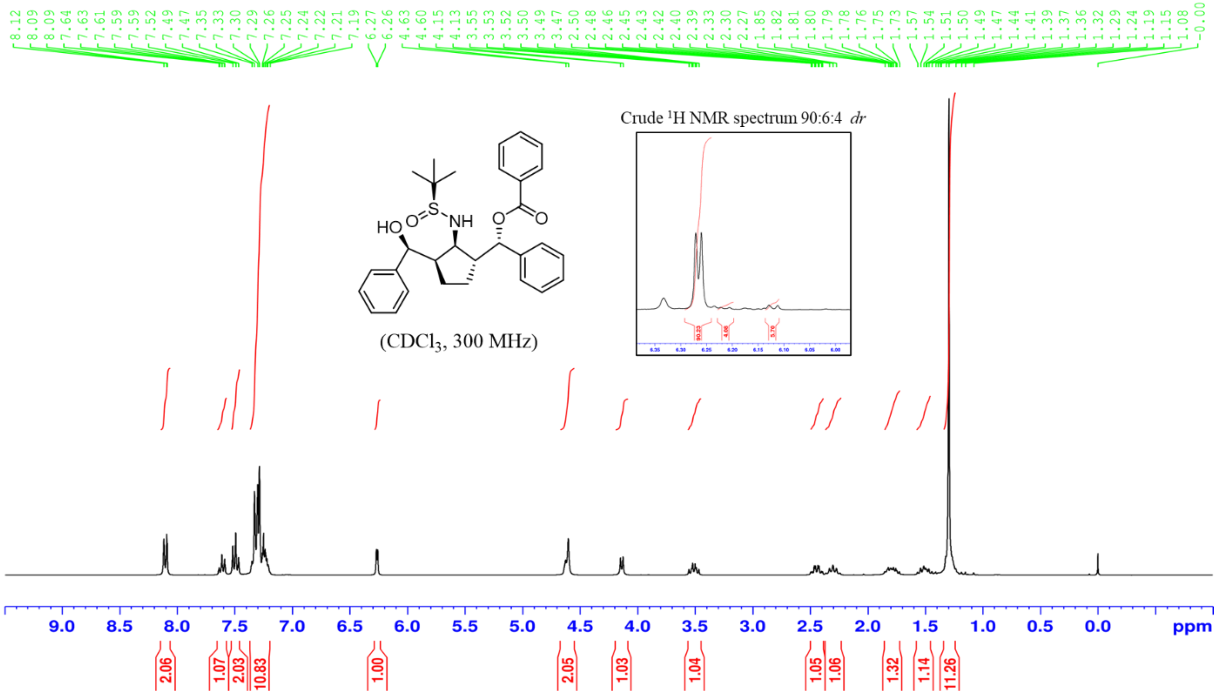

(*R*)-((1*R*,2*R*,3*R*)-2-(((*S*)-*Tert*-butylsulfinyl)amino)-3-((*S*)-hydroxy(phenyl)methyl)cyclopentyl)(phenyl)methyl benzoate, 3a

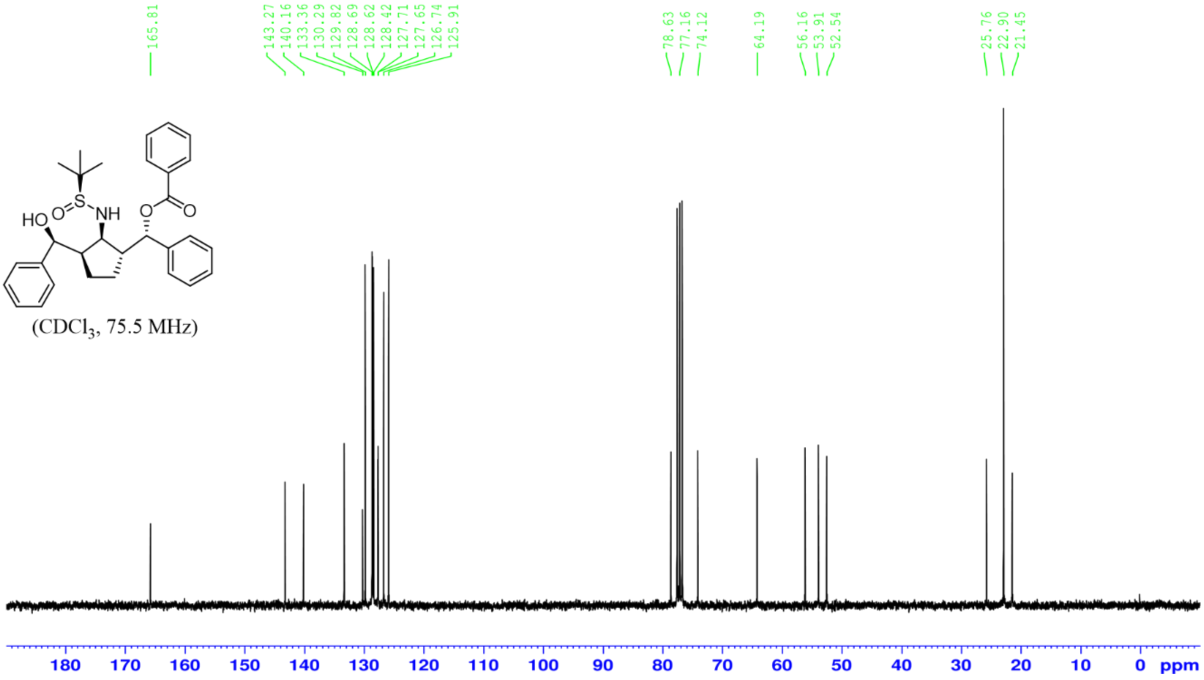

(*R*)-((1*R*,2*R*,3*R*)-2-(((*S*)-*Tert*-butylsulfinyl)amino)-3-((*S*)-(4-ethylphenyl)(hydroxy)methyl)cyclopentyl)(4-ethylphenyl)methyl 4-ethylbenzoate, 3b

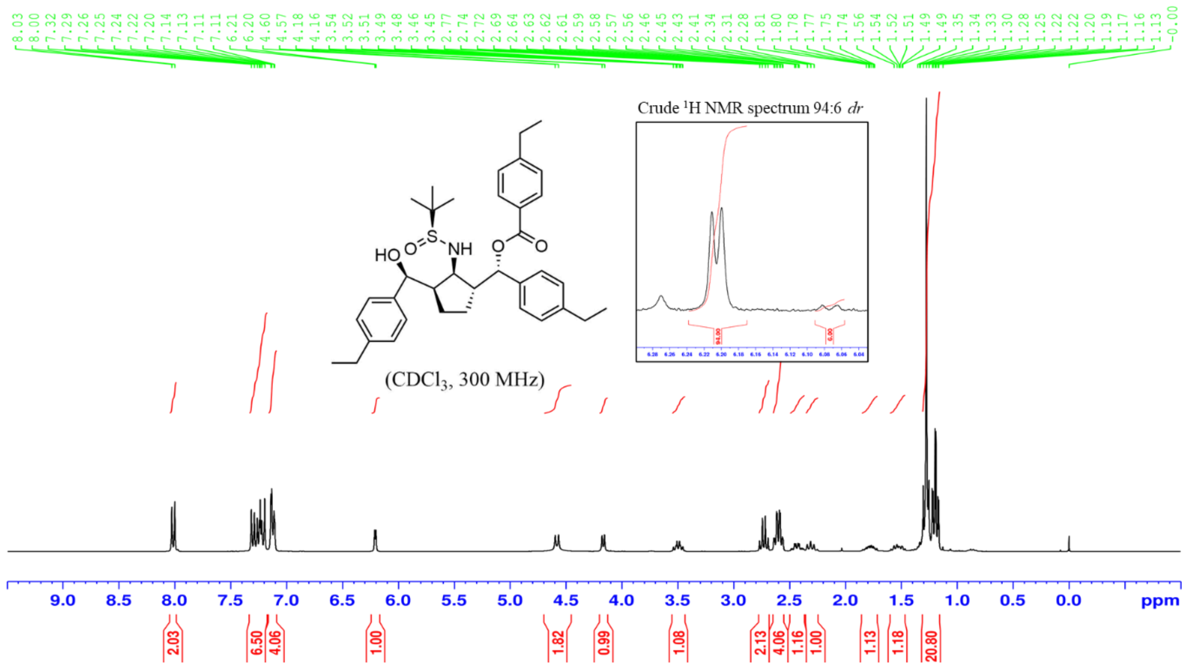

(*R*)-((1*R*,2*R*,3*R*)-2-(((*S*)-*Tert*-butylsulfinyl)amino)-3-((*S*)-(4-ethylphenyl)(hydroxy)methyl)cyclopentyl)(4-ethylphenyl)methyl 4-ethylbenzoate, 3b

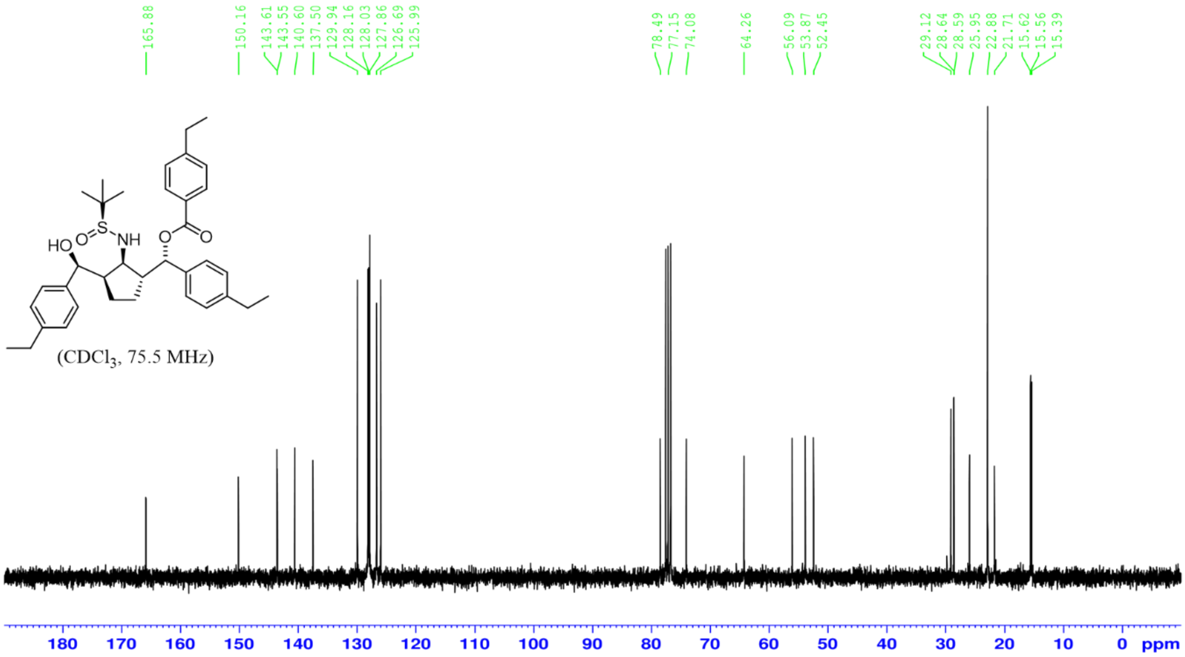

(*R*)-((1*R*,2*R*,3*R*)-2-(((*S*)-*Tert*-butylsulfinyl)amino)-3-((*S*)-hydroxy(4-isopropylphenyl)methyl)cyclopentyl)(4-isopropylphenyl)methyl 4-isopropylbenzoate, 3c

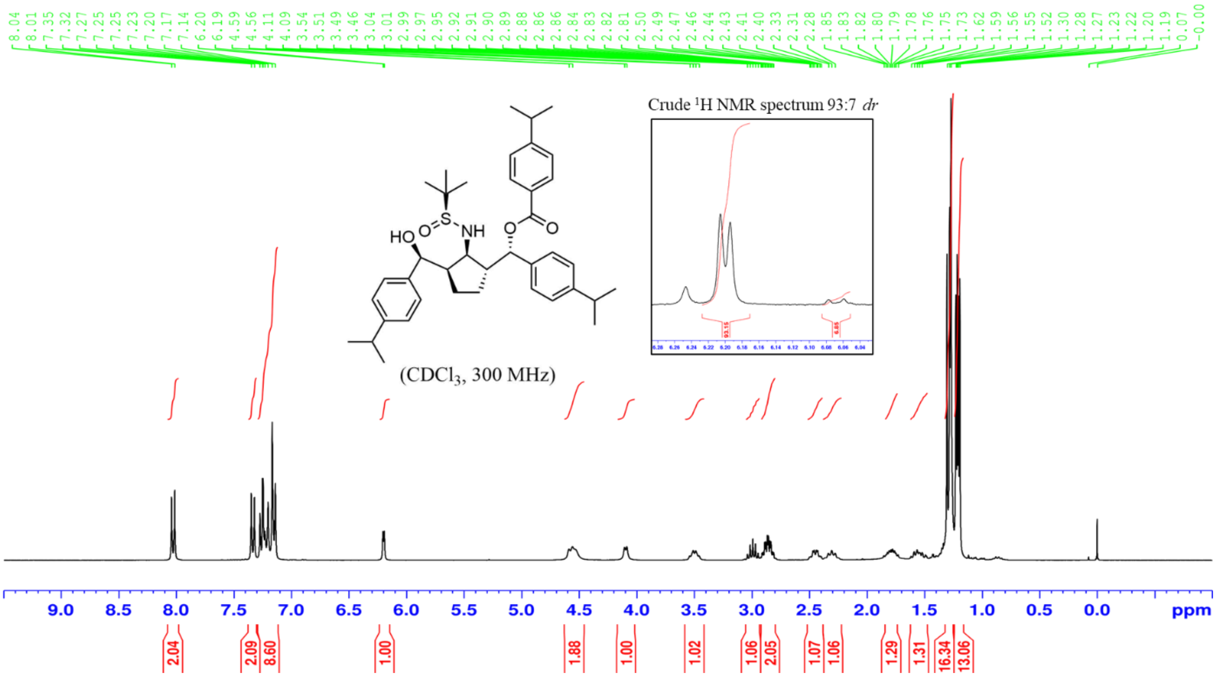

(*R*)-((1*R*,2*R*,3*R*)-2-(((*S*)-*Tert*-butylsulfinyl)amino)-3-((*S*)-hydroxy(4-isopropylphenyl)methyl)cyclopentyl)(4-isopropylphenyl)methyl 4-isopropylbenzoate, 3c

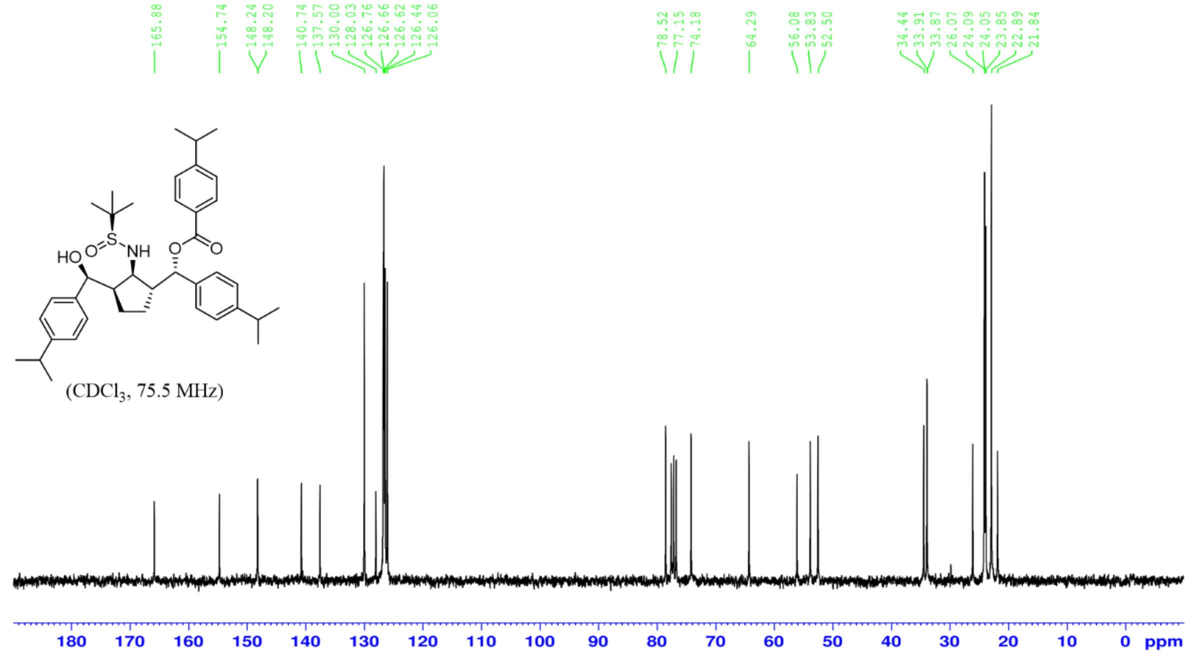

3-(((*S*)-*Tert*-butylsulfinyl)amino)-5-hydroxy-4-methyl-1,5-diphenylpentyl benzoate, 4a

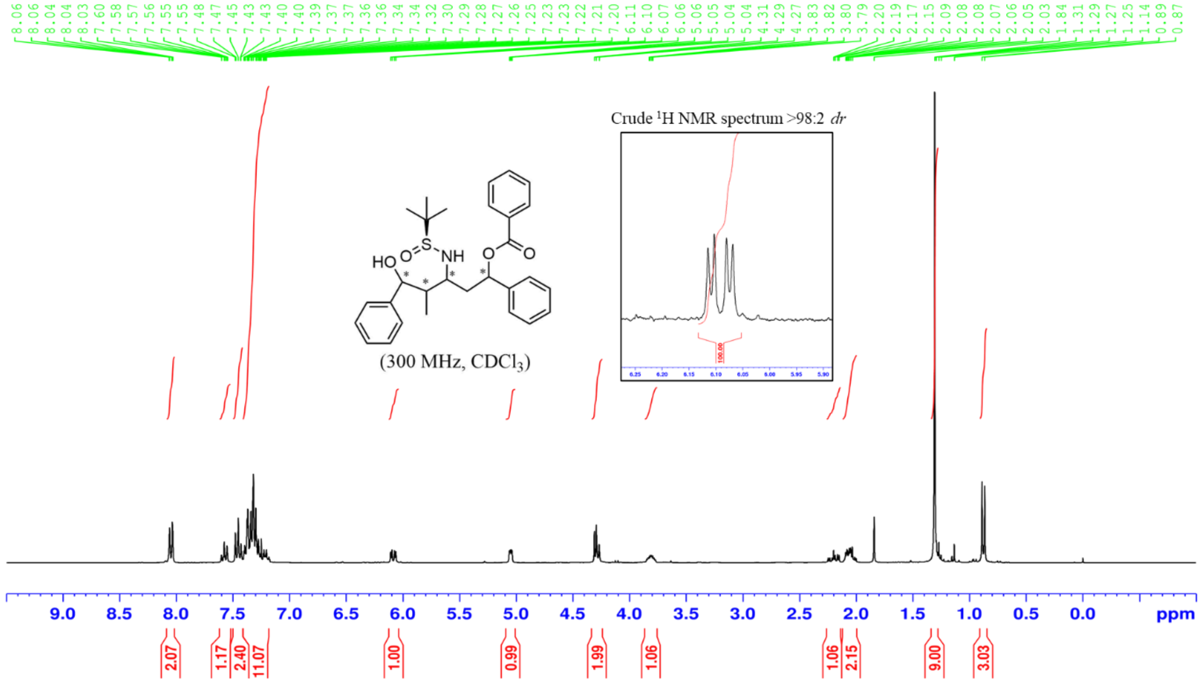

3-(((*S*)-*Tert*-butylsulfinyl)amino)-5-hydroxy-4-methyl-1,5-diphenylpentyl benzoate, 4a

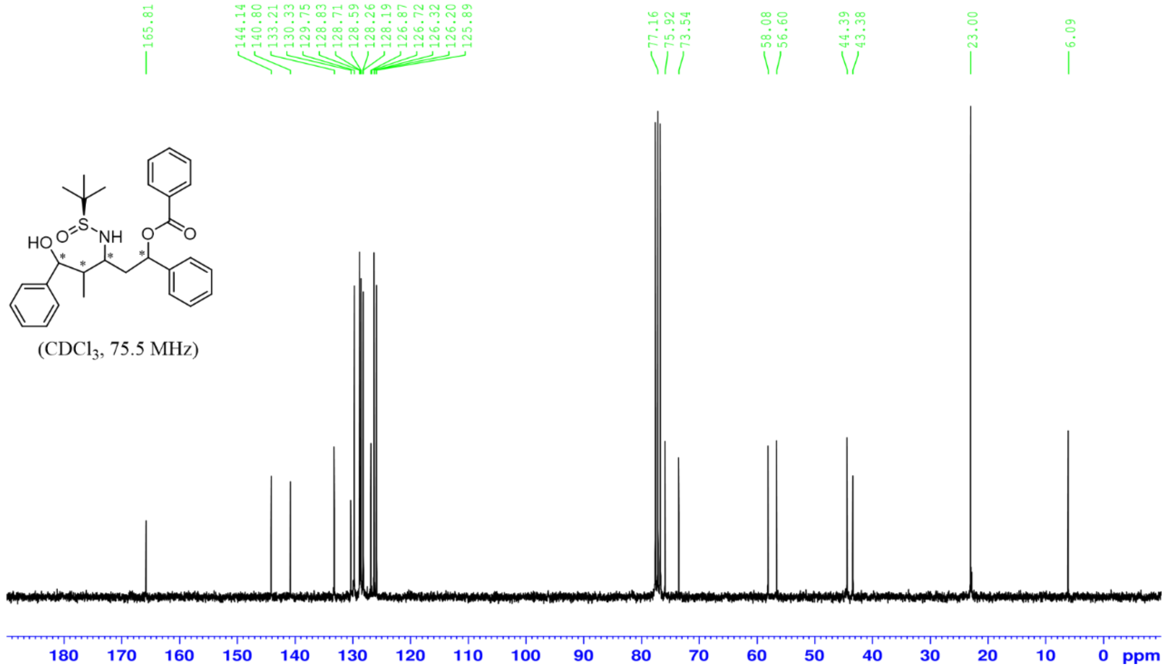

3-(((*S*)-*Tert*-butylsulfinyl)amino)-5-hydroxy-4-methyl-1,5-di-*p*-tolylpentyl 4-methylbenzoate, 4b

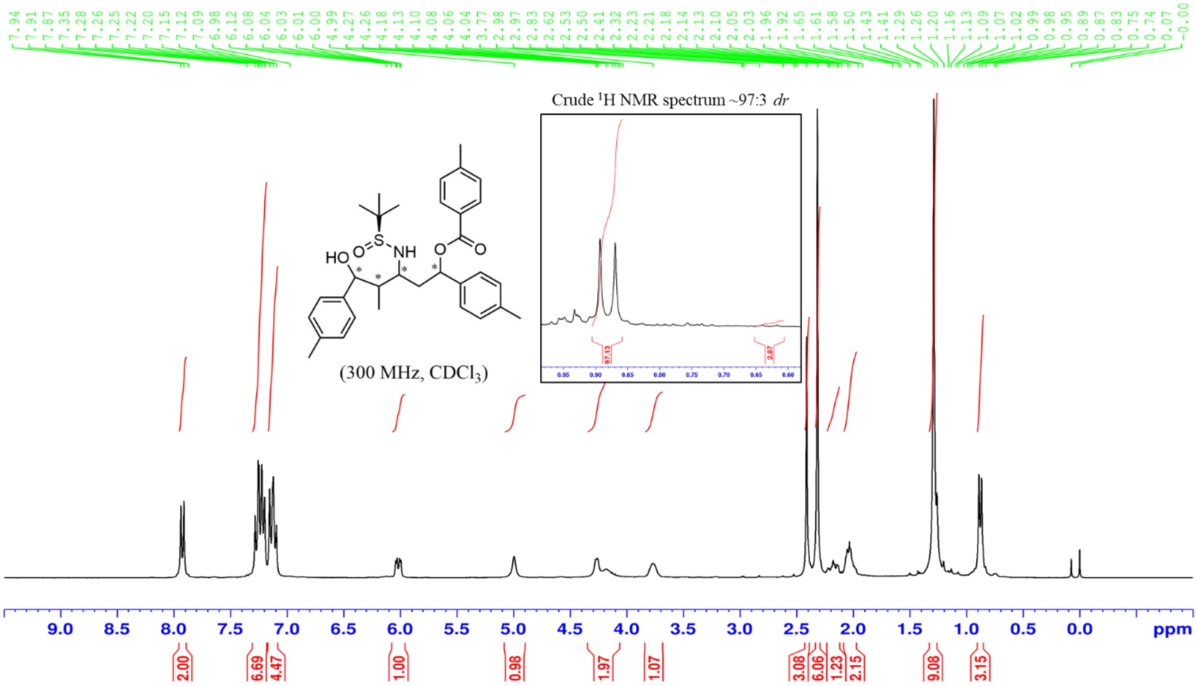

3-(((*S*)-*Tert*-butylsulfinyl)amino)-5-hydroxy-4-methyl-1,5-di-*p*-tolylpentyl 4-methylbenzoate, 4b

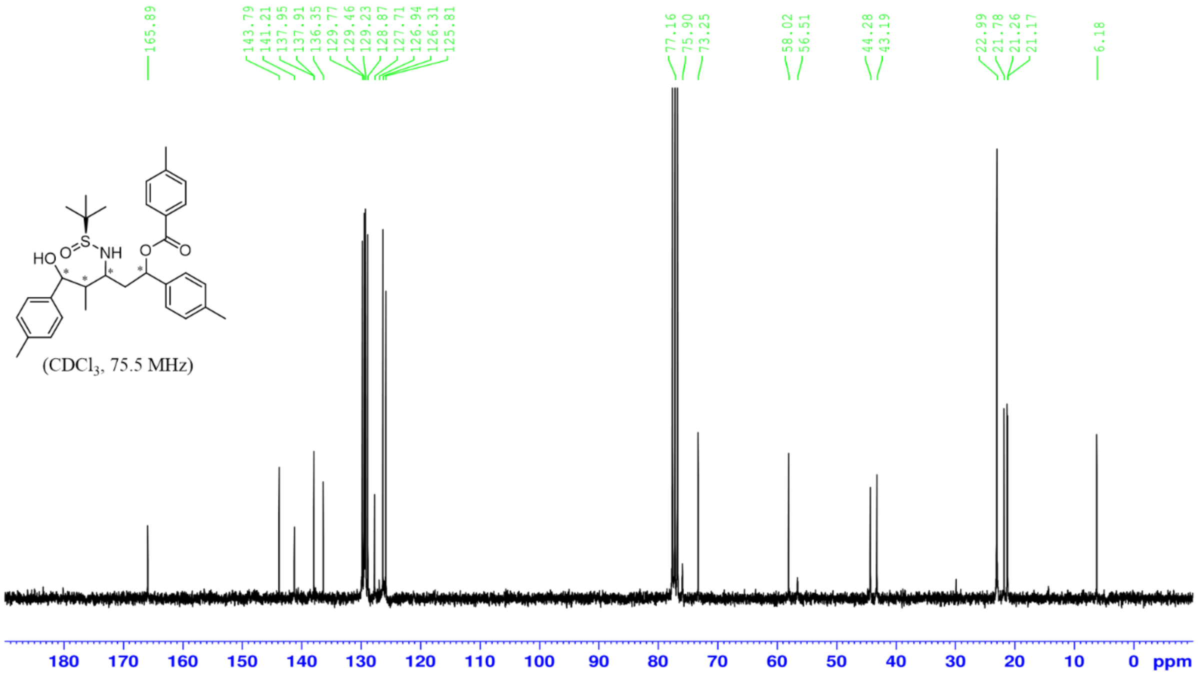

3-(((*S*)-*Tert*-butylsulfinyl)amino)-5-hydroxy-4-methyl-1,5-di-*m*-tolylpentyl 3-methylbenzoate, 4c

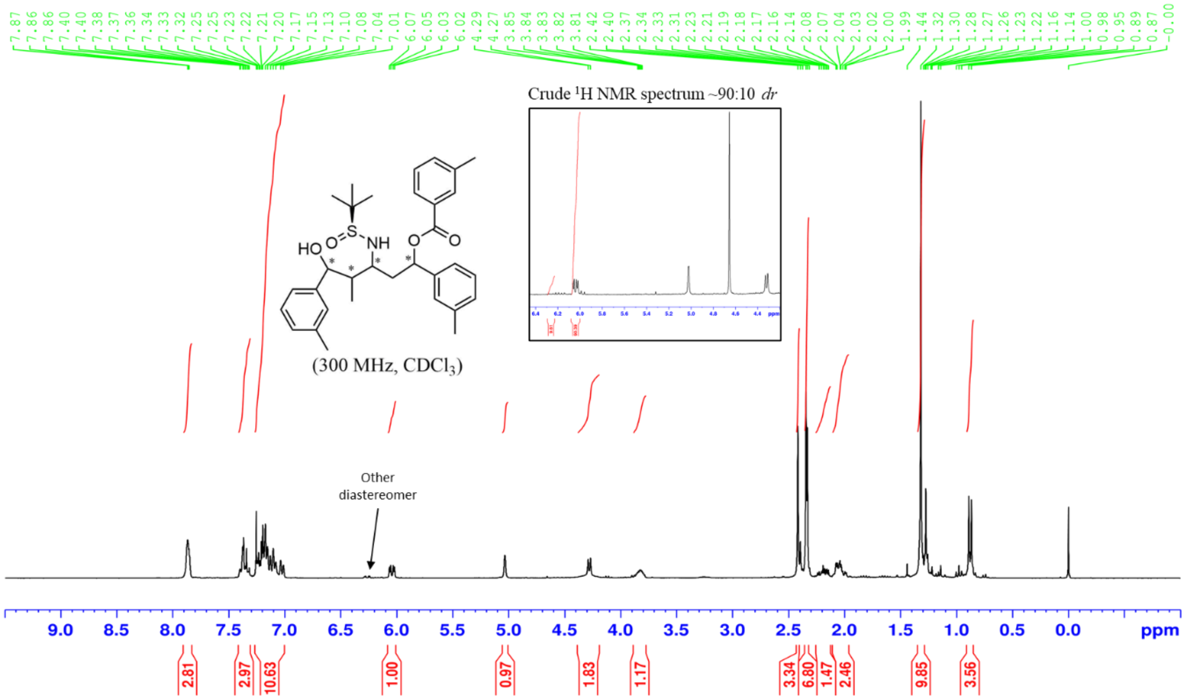

3-(((*S*)-*Tert*-butylsulfinyl)amino)-5-hydroxy-4-methyl-1,5-di-*m*-tolylpentyl 3-methylbenzoate, 4c

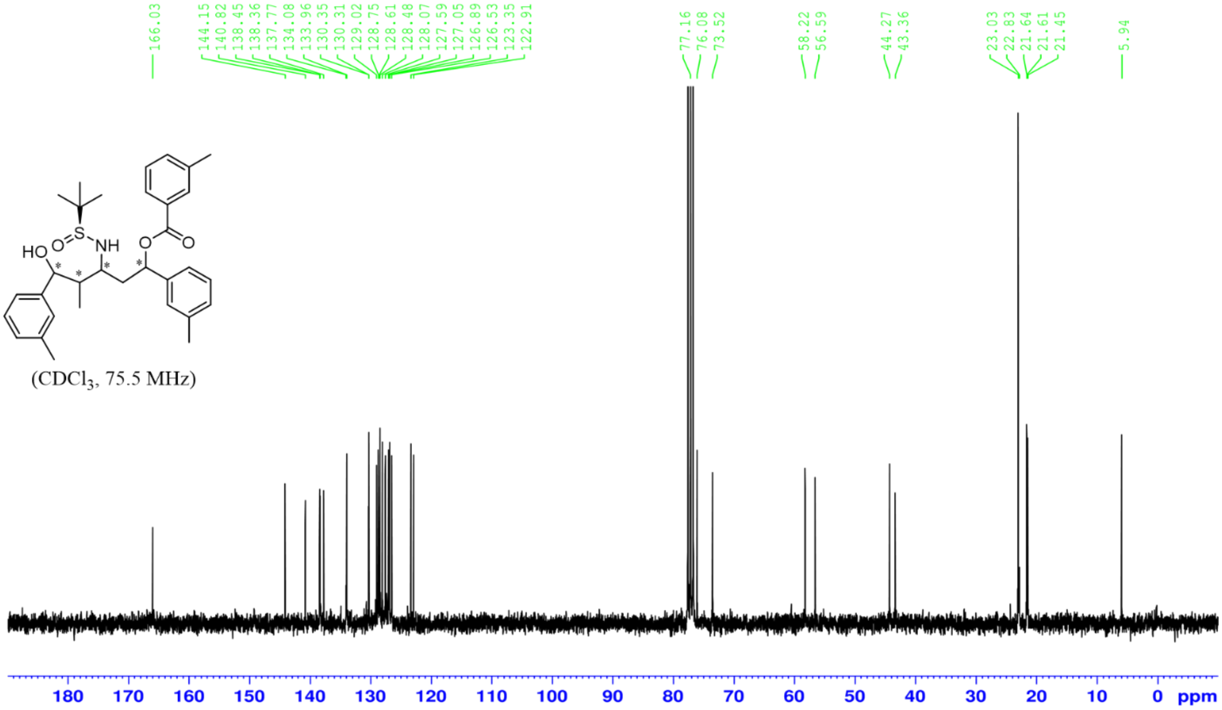

3-(((*S*)-*Tert*-butylsulfinyl)amino)-1,5-bis(3-fluorophenyl)-5-hydroxy-4-methylpentyl 3-fluorobenzoate, 4d

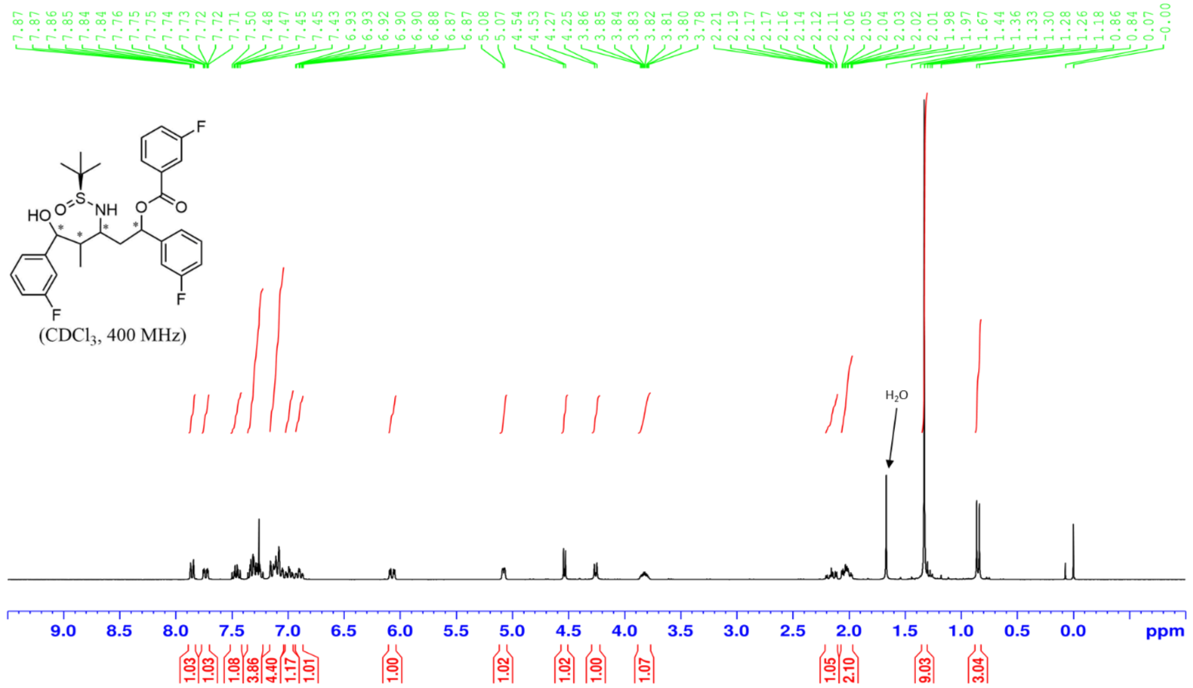

3-(((*S*)-*Tert*-butylsulfinyl)amino)-1,5-bis(3-fluorophenyl)-5-hydroxy-4-methylpentyl 3-fluorobenzoate, 4d

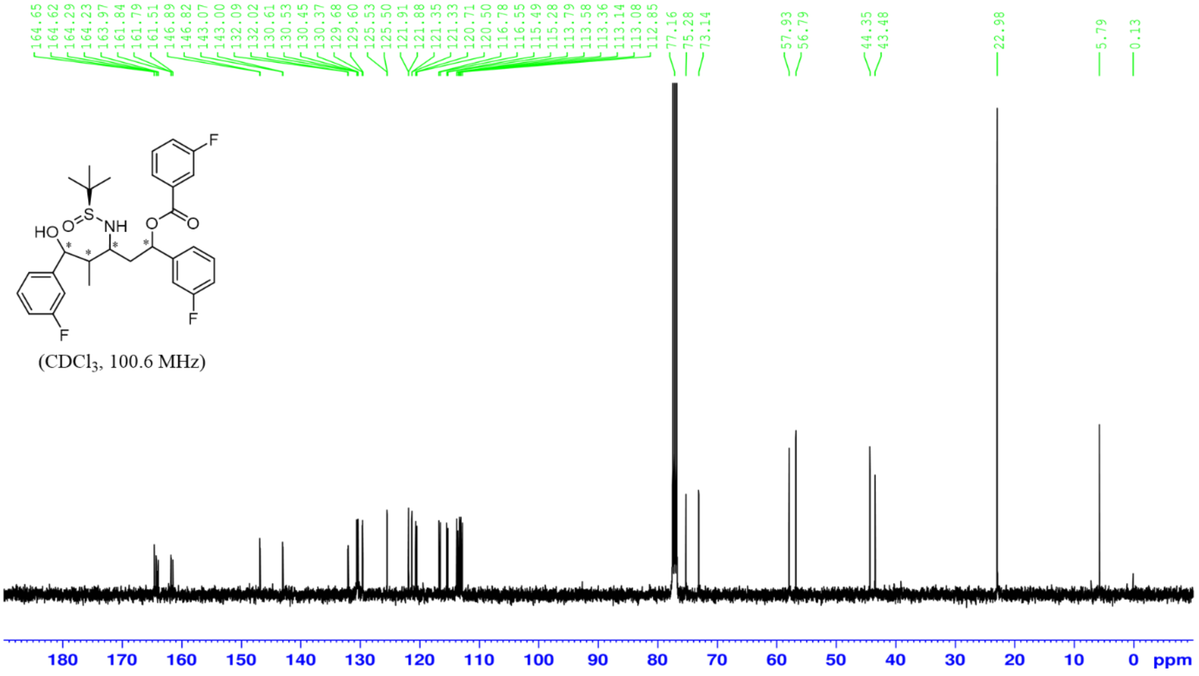

(*R*)-((1*R*,2*R*,3*R*)-2-Amino-3-((*S*)-(3-fluorophenyl)(hydroxy)methyl)cycloheptyl)(3-fluorophenyl)methyl 3-fluorobenzoate, 2q

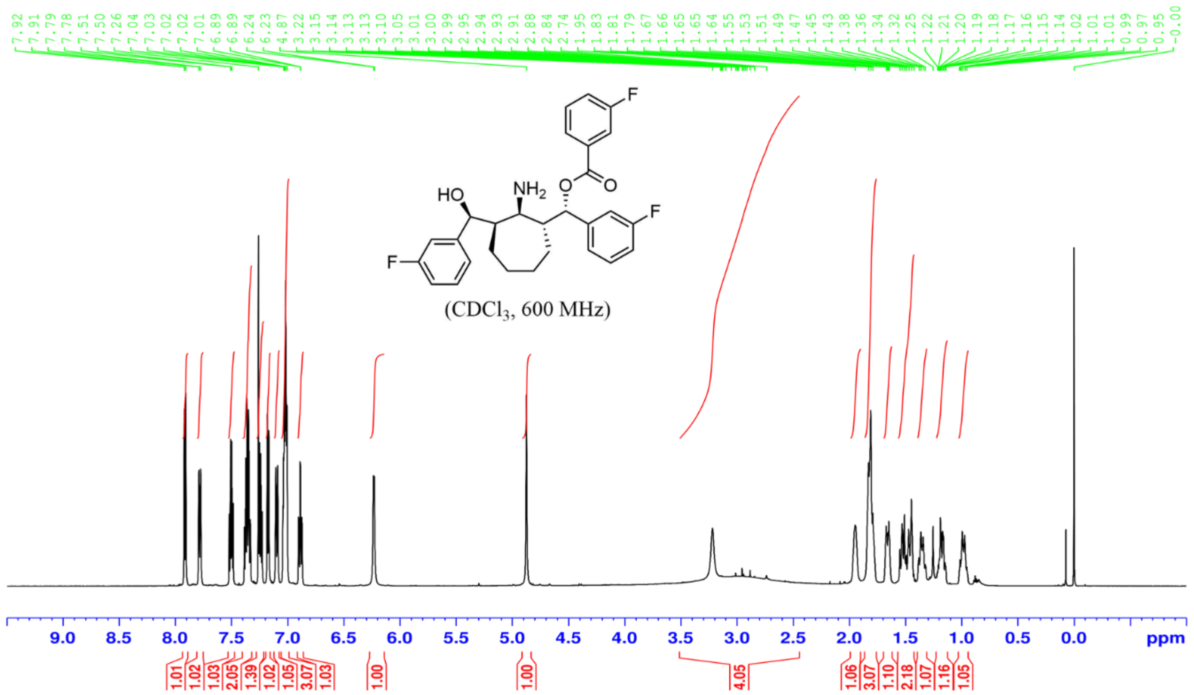

(*R*)-((1*R*,2*R*,3*R*)-2-Amino-3-((*S*)-(3-fluorophenyl)(hydroxy)methyl)cycloheptyl)(3-fluorophenyl)methyl 3-fluorobenzoate, 2q

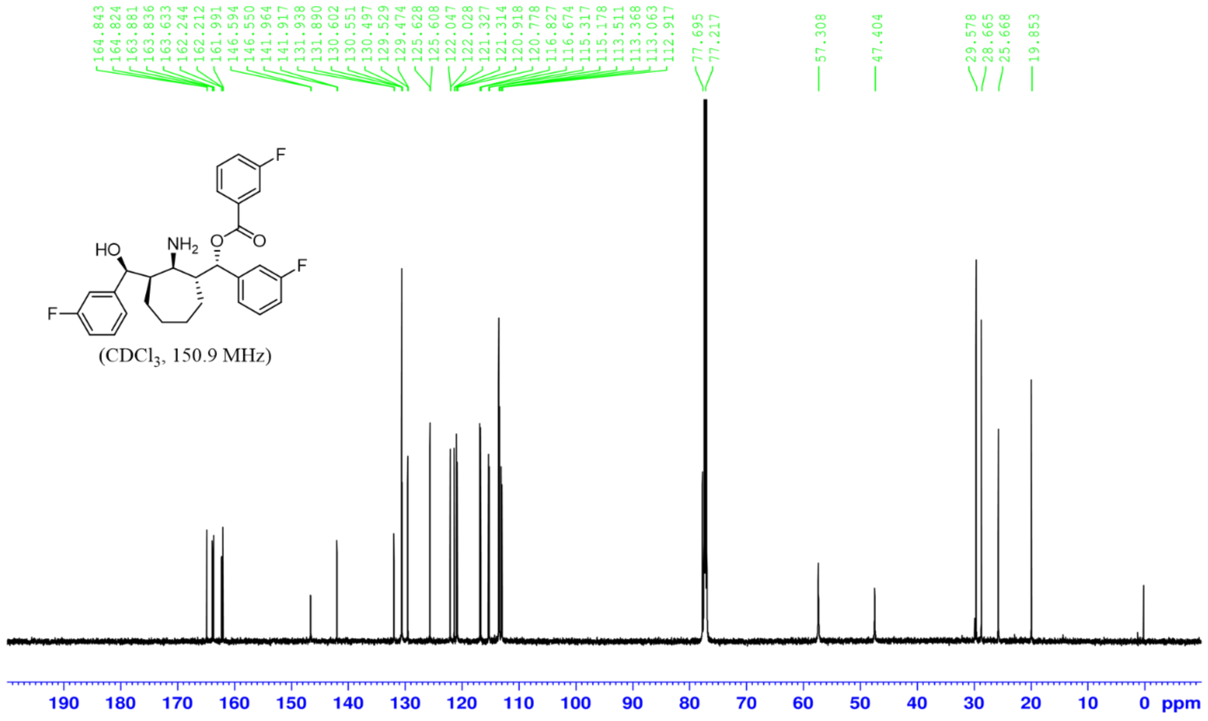

XIII. Crystallographic data for compounds 2j, 2k and 2s

Crystallographic structure for (*R*)-((1*R*,2*R*,3*R*)-2-(((*S*)-*Tert*-butylsulfinyl)amino)-3-((*S*)-hydroxy(4-(trifluoromethoxy)phenyl)methyl)cycloheptyl)(4-(trifluoromethoxy)phenyl)methyl 4-(trifluoromethoxy)benzoate, 2j

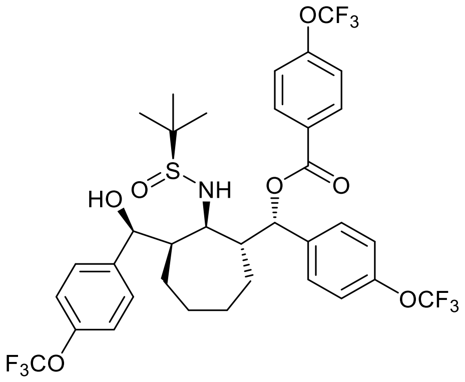

NOTE: The crystal structure has been lodged at the Cambridge Crystallographic Data Centre and assigned the deposition number CCDC 1905742.

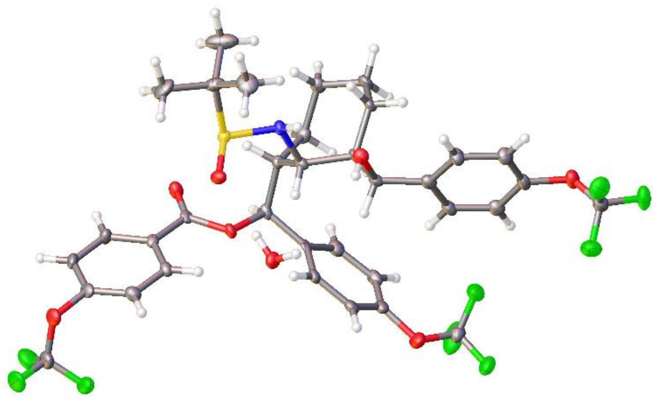

Fig. SI-9. Thermal ellipsoids drawn at the 50% probability level, second molecule omitted for clarity.

Experimental. Single clear yellow block-shaped crystals of compound **2j** recrystallised from a mixture of DCM and hexane by slow evaporation. A suitable crystal 0.23 × 0.09 × 0.07 mm^3 was selected and mounted on a MITIGEN holder silicon oil on an Rigaku AFC12 FRE-HF diffractometer. The crystal was kept at a steady T = 100(2) K during data collection. The structure was solved with the ShelXT (Sheldrick, 2015) structure solution program using the Intrinsic Phasing solution method and by using Olex2 (Dolomanov et al., 2009) as the graphical interface. The model was refined with version 2016/6 of ShelXL (Sheldrick, 2015) using Least Squares minimisation.

Crystal Data. $C_{35}H_{37}F_9NO_{7.5}S$, M_r = 794.71, D_{calc} = 1.450 g cm^{-3}, orthorhombic, $P2_12_12_1$ (No. 19), a =

17.5557(5) Å, b = 19.9884(8) Å, c = 20.7472(6) Å, $\alpha = \beta = \gamma = 90^{\circ}$, V = 7280.4(4) Å^3, T = 100(2) K, Z = 8, Z' = 2, λ = 0.71073 Å, μ(MoK$_{\alpha}$) = 0.185, 86253 reflections measured, 18143 unique (R_{int} = 0.1231) which were used in all calculations. GooF = 1.111. The final wR_2 was 0.1288 (all data) and R_1 was 0.0880 (I > 2(I)). The Flack parameter was refined to 0.05(6).

Crystallographic structure for (*R*)-((1*R*,2*R*,3*R*)-2-(((*S*)-*Tert*-butylsulfinyl)amino)-3-((*S*)-(4-fluorophenyl)(hydroxy)methyl)cycloheptyl)(4-fluorophenyl)methyl 4-fluorobenzoate, 2k

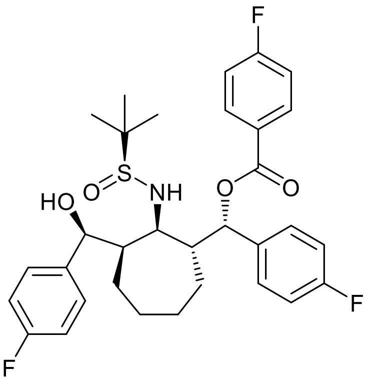

NOTE: The crystal structure has been lodged at the Cambridge Crystallographic Data Centre and assigned the deposition number CCDC 1877296.

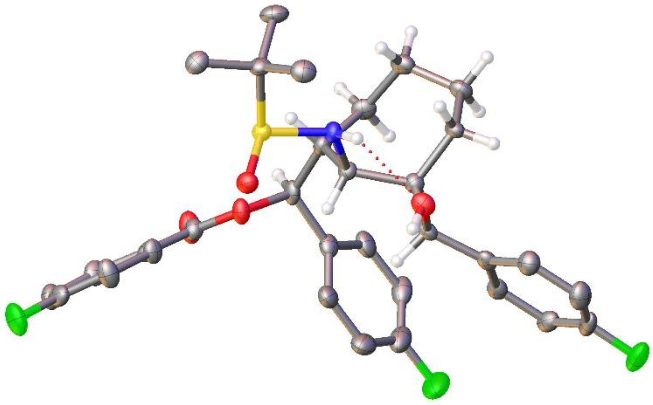

Fig. SI-10. Thermal ellipsoids drawn at the 50% probability level. Selected hydrogens, equivalent molecules & water omitted for clarity.

Experimental. Single clear colourless irregular-shaped crystals of **2k** recrystallised from a mixture of Et_2O and hexane by slow evaporation. A suitable crystal 0.24 × 0.15 × 0.02 mm^3 was selected and mounted on a MITIGEN holder silicon oil on a Rigaku AFC12 FRE-VHF diffractometer. The crystal was kept at a steady T = 100(2) K during data collection. The structure was solved with the ShelXT (Sheldrick, 2015) structure solution program using the dual solution method and by using Olex2 (Dolomanov et al., 2009) as the graphical interface. The model was refined with version 2016/6 of ShelXL (Sheldrick, 2015) using Least Squares minimisation.

Crystal Data. $C_{32}H_{36.5}F_3NO_{4.25}S$, M_r = 592.18, D_{calc} = 1.186 g cm^{-3} orthorhombic, $P2_12_12$ (No. 18), a = 41.6676(17) Å, b = 22.1967(10) Å, c = 14.3476(4) Å, $\alpha = \beta = \gamma = 90^{\circ}$, V = 13269.9(9) Å^3, T = 100(2) K, Z = 16, Z' = 4, λ = 0.71073 Å, $\mu(\text{MoK}_{\alpha})$ = 0.149, 104314 reflections measured, 33638 unique (R_{int} = 0.0794) which were used in all calculations. GooF = 1.025. The final wR_2 was 0.1310 (all data) and R_1 was 0.0630 (I > 2(I)). The Flack parameter was refined to -0.02(3).

Crystallographic structure for (*S*)-*N*-((2*R*,7*R*)-2,7-Bis((*S*)-furan-2-yl(hydroxy)methyl)cycloheptyl)-2-methylpropane-2-sulfinamide, 2s

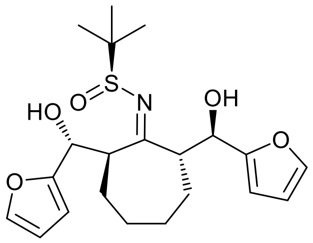

NOTE: The crystal structure has been lodged at the Cambridge Crystallographic Data Centre and assigned the deposition number CCDC 1897008.

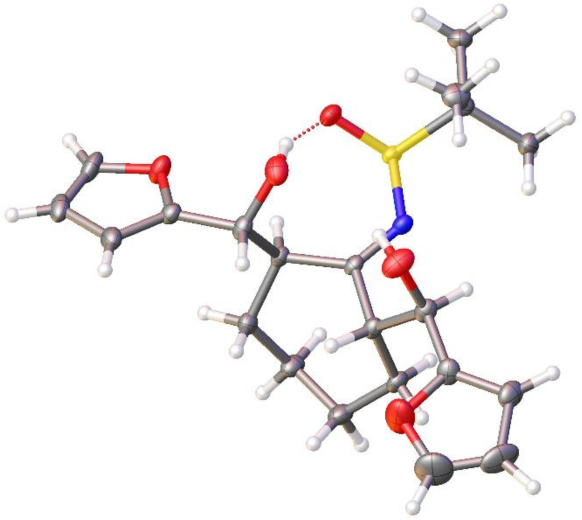

Fig. SI-11. Thermal ellipsoids drawn at the 50% probability level.

Experimental. Single clear colourless prism-shaped crystals of **2s** were recrystallised from a mixture of DCM and hexane by slow evaporation. A suitable crystal 0.39 × 0.29 × 0.10 mm^3 was selected and mounted on a MITIGEN holder silicon oil on an Rigaku AFC12 FRE-HF diffractometer. The crystal was kept at a steady T = 100(2) K during data collection. The structure was solved with the ShelXT (Sheldrick, 2015) structure solution program using the Intrinsic Phasing solution method and by using Olex2 (Dolomanov et al., 2009) as the graphical interface. The model was refined with version 2016/6

of ShelXL (Sheldrick, 2015) using Least Squares minimisation.

Crystal Data. $C_{21}H_{29}NO_5S$, M_r = 407.51, D_{calc} = 1.287 g cm^{-3} monoclinic, *C*2 (No. 5), a = 20.0761(4) Å, b = 8.9229(2) Å, c = 12.1819(3) Å, β = 105.531(2)°, $\alpha = \gamma$ = 90°, *V* = 2102.55(8) Å^3, *T* = 100(2) K, *Z* = 4, *Z'* = 1, λ = 0.71073 Å, μ(MoK$_\alpha$) = 0.185, 24997 reflections measured, 5564 unique (R_{int} = 0.0526) which were used in all calculations. GooF = 1.033. The final wR_2 was 0.0882 (all data) and R_1 was 0.0370 (I > 2(I)). The Flack parameter was refined to 0.07(3).
